# Suppression of alpha-carbon racemization in peptide synthesis based on a thiol-labile amino protecting group

**DOI:** 10.1038/s41467-023-41115-x

**Published:** 2023-09-01

**Authors:** Yifei Zhou, Hongjun Li, Yi Huang, Jiahui Li, Guiyu Deng, Gong Chen, Zhen Xi, Chuanzheng Zhou

**Affiliations:** State Key Laboratory of Elemento-Organic Chemistry, Frontiers Science Center for New Organic Matter, Department of Chemical Biology, College of Chemistry, Nankai University, Tianjin, 300071 China

**Keywords:** Peptides, Solid-phase synthesis

## Abstract

In conventional solid-phase peptide synthesis (SPPS), α-amino groups are protected with alkoxycarbonyl groups (e.g., 9-fluorenylmethoxycarbonyl [Fmoc]). However, during SPPS, inherent side reactions of the protected amino acids (e.g., α-C racemization and aspartimide formation) generate by-products that are hard to remove. Herein, we report a thiol-labile amino protecting group for SPPS, the 2,4-dinitro-6-phenyl-benzene sulfenyl (DNPBS) group, which is attached to the α-amino group via a S–N bond and can be quantitatively removed in minutes under nearly neutral conditions (1 M *p*-toluenethiol/pyridine). The use of DNPBS greatly suppresses the main side reactions observed during conventional SPPS. Although DNPBS SPPS is not as efficient as Fmoc SPPS, especially for synthesis of long peptides, DNPBS and Fmoc are orthogonal protecting groups; and thus DNPBS SPPS and Fmoc SPPS can be combined to synthesize peptides that are otherwise difficult to obtain.

## Abstract (web summary)

During solid-phase peptide synthesis (SPPS) inherent side reactions of the protected amino acids such as α-C racemization generate by-products that are hard to remove. Here, the authors report thiol-labile amino protecting group for SPPS, DNPBS group, which suppresses the main side reactions observed during conventional SPPS.

## Introduction

**Fig. 1:**
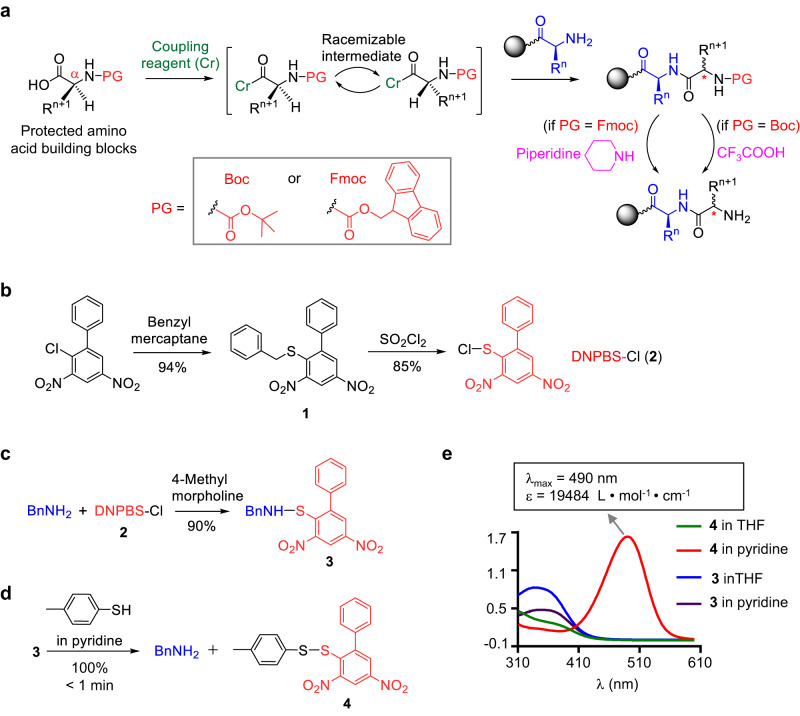
α-Amino protecting groups used for solid-phase peptide synthesis (SPPS). **a** Boc SPPS and Fmoc SPPS. **b** Synthesis of DNPBS-Cl (**2**). **c** Protection of benzylamine (BnNH_2_) by DNPBS to afford **3**. **d** Removal of DNPBS from **3**. **e** UV absorption spectra of **3** and **4** in THF and pyridine. DNPBS, 2,4-dinitro-6-phenyl-benzene sulfenyl.

Solid-phase peptide synthesis (SPPS), which was developed by Nobel laureate R. Bruce Merrifield in the early 1960s^1,2^, is the most commonly used method for preparing peptides^3–5^. Protection of the α-amino group of the amino acids is key to the success of SPPS. The 9-fluorenylmethoxycarbonyl (Fmoc) and *tert*-butyloxycarbonyl (Boc) groups are widely used for this purpose, and SPPS processes that use these groups are referred to as Fmoc SPPS and Boc SPPS, respectively (Fig. 1a)^6–8^. The former is simpler and more reliable than the latter and thus is used more often.

Both Fmoc- and Boc-protected amino acids have a carbamate moiety attached to the α-C. Activation of the protected amino acid by the coupling reagent generates a racemizable intermediate during peptide bond formation and thus leads to α-C racemic products that are hard to remove by routine purification methods (Fig. 1a)^9,10^. In addition, Boc and Fmoc are removed by treatment with a strong acid (trifluoroacetic acid [TFA]) or base (piperidine), respectively, and these harsh deprotection conditions lead to undesirable side reactions, such as aspartimide and piperidide formation^10–12^.

Here, we report a thiol-labile amino protecting group, the 2,4-dinitro-6-phenyl-benzene sulfenyl (DNPBS) group, for SPPS. The use of DNPBS greatly suppresses the main side reactions observed in conventional SPPS.

## Results

### Design, synthesis, and testing of the DNPBS α-amino protecting group

Thiols such as glutathione and cysteine are biocompatible soft nucleophiles that show striking chemical reactivity under physiological conditions. Temporary protection of bioactive molecules with thiol-liable protecting groups has been widely used in biomedical research and therapeutic applications^13^. Therefore, we reasoned that SPPS based on a thiol-liable α-amino protecting group might be superior to conventional SPPS. 2,4-Dinitrophenylsulfonyl^13^, dithiasuccinoyl^14^, and *O*-nitrophenylsulfenyl^15^ protecting groups have been tried for this purpose, but they suffer from either difficult synthesis or inefficient removal. However, introduction and removal of the 2,4-dinitrobenzenesulfenyl group, which was developed for the protection of the 5′-OH of nucleosides, is convenient and efficient^16^. Therefore, we synthesized a series of 2,4-dinitrobenzenesulfenyl derivatives and eventually found that DNPBS was an ideal thiol-liable α-amino protecting group for SPPS.

The protecting reagent, DNPBS-Cl (**2**), was synthesized by treatment of 1-chloro-2-phenyl-4,6-dinitrobenzene with benzyl mercaptane in the presence of Et_3_N to give **1** (94% yield), which was then treated with SO_2_Cl_2_ to afford **2** (85% yield, Fig. 1b). The amino group of benzylamine could be protected by treatment with **2** in the presence of 4-methylmorpholine to afford **3** in 90% yield (Fig. 1c). Incubation of **3** with 1 M *p*-toluenethiol in pyridine at room temperature resulted in quantitative removal of the DNPBS moiety in less than 1 min (Fig. 1d). During the deprotection step, the color of the reaction mixture changed from light yellow to dark red. Analysis of the products revealed that the released DNPBS moiety was transformed to disulfane **4**, which has a λ_max_ of 490 nm in pyridine and thus was responsible for the red color (Fig. 1e). In contrast, a pyridine solution of protected benzylamine (**3**) had a λ_max_ of 350 nm and did not absorb at 490 nm. Furthermore, neither **3** nor **4** absorbed at a wavelength of >420 nm in THF, a less polar solvent. The color change that occurs during the deprotection reaction had the advantage of allowing us to monitor the reaction visually or by UV–vis spectrometry.

### Preparation of DNPBS-protected amino acids

**Fig. 2:**
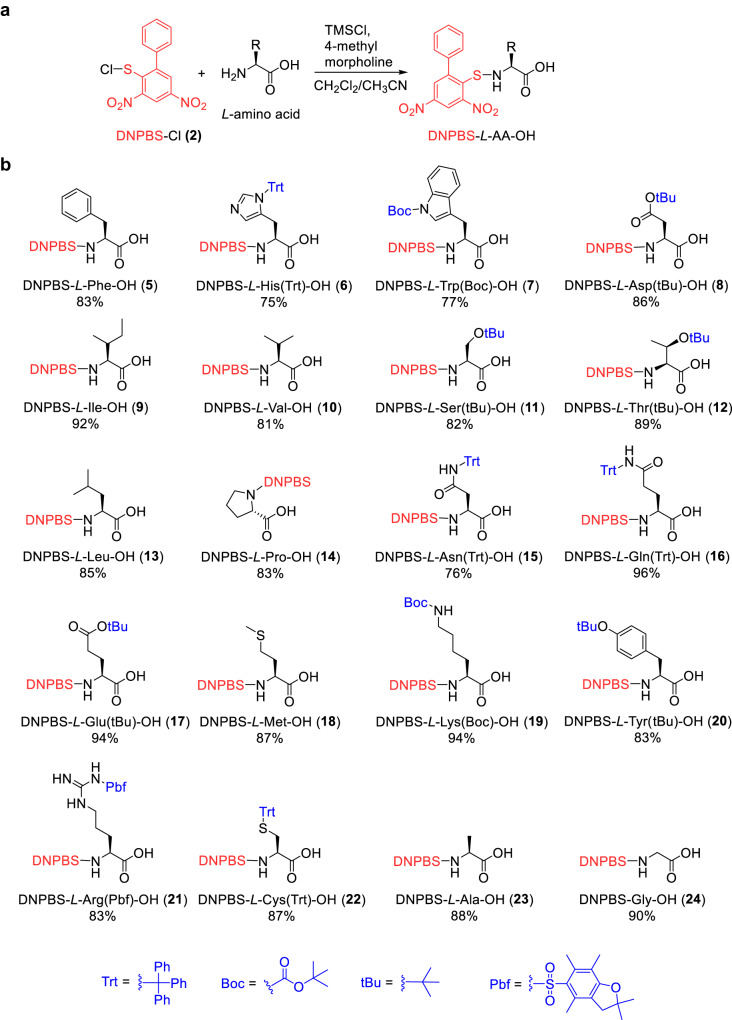
Synthesis of DNPBS-protected amino acids. **a** General synthetic strategy of DNPBS-protected amino acids. **b** Structures of DNPBS-protected amino acids. DNPBS, 2,4-dinitro-6-phenyl-benzene sulfenyl.

Because the deprotecting reagent (1 M *p*-toluenethiol in pyridine) for DNPBS SPPS is weakly basic, we protected the amino acid side chains with the acid-labile protecting groups that are used for Fmoc SPPS. The side-chain-protected *l*-amino acids were treated with trimethylsilyl chloride and 4-methyl morpholine in dichloromethane (DCM)/acetonitrile (MeCN) and subsequently with DNPBS-Cl (**2**). After reaction at room temperature for 20 min followed by simple precipitation and/or recrystallization, pure DNPBS-protected amino acids **5**–**24** were obtained in 75–96% yields (Fig. 2). The ^1^H NMR spectra of a solution of DNPBS-*l*-Ile-OH (**9**) in deuterated dimethyl formamide (DMF-d_7_) or THF-d_8_ showed no decomposition after up to 30 days at room temperature (Supplementary Fig. 1), indicating that DNPBS is a stable α-amino protecting group.

Because some Fmoc-protected amino acids are poorly soluble in low-polarity solvents, only highly polar solvents such as DMF and *N*-methylpyrrolidone can be used for Fmoc SPPS^9,17^. In contrast, all the DNPBS-protected amino acids we prepared exhibited good solubility (>0.1 M) in a broad range of organic solvents, including but not limited to DCM, EtOAc, DMF, and THF. This feature can be expected to allow peptide synthesis in greener solvents^18,19^ Of note, DNPBS-protected amino acids are insoluble in water, which is considered to be the most sustainable solvent for peptide synthesis^20–23^.

### Effect of coupling reagent on α-C racemization of His, Cys, and Ser

**Fig. 3:**
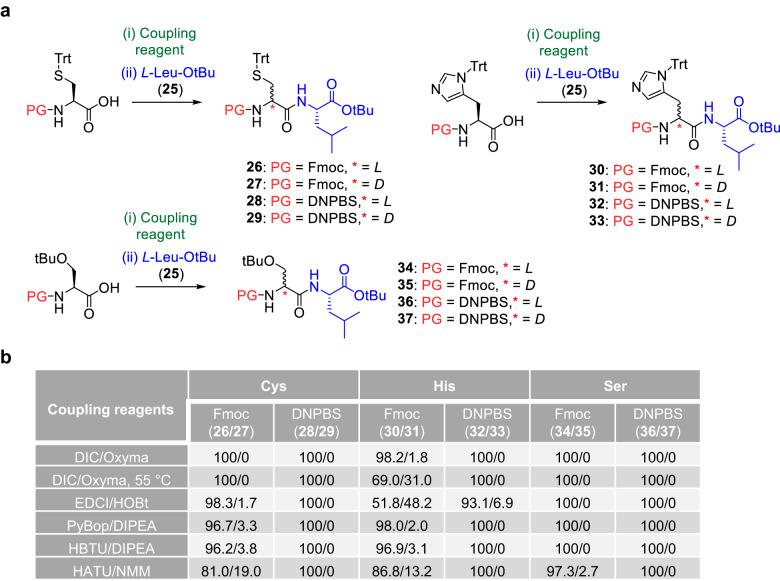
Effect of coupling reagent on α-C racemization of His, Cys, and Ser during coupling. **a** Reactions of *l*-His(Trt)-OH, *l*-Ser(tBu)-OH, and *l*-Cys(Trt)-OH with *l*-Leu-OtBu (**25**) in the presence of various coupling reagents at room temperature unless specified otherwise. **b** Ratio of *l*-product/*d*-product (as determined by chiral chromatography) during coupling reactions. DNPBS, 2,4-dinitro-6-phenyl-benzene sulfenyl; Fmoc, 9-fluorenylmethoxycarbonyl; DIC, diisopropylcarbodiimide; DIPEA, diisopropylethylamine; EDCI, *N*-ethyl-*N*’-(3-dimethylaminopropyl)carbodiimide hydrochloride; HATU, *N-*[(dimethylamino)−1*H*-1,2,3-triazolo[4,5-*b*]-pyridin-1-ylmethylene]-*N*-methylmethanaminium hexafluorophosphate *N*-oxide; HBTU, *N*-[(1H-benzotriazol-1-yl)(dimethylamino)-methylene]-*N*-methylmethanaminium hexafluorophosphate *N*-oxide; HOBt, 1-hydroxybenzotriazole; NMM: *N*-methylmorpholine; Oxyma, ethyl 2-cyano-2-(hydroxyimino)acetate; PyBop, benzotriazol-1-yloxytri(pyrrolidino) phosphonium hexafluorophosphate.

As mentioned above, activation of the Fmoc-amino acids during peptide bond coupling leads to racemization at the α-C (especially for His, Cys, and Ser)^5^, and the extent of racemization depends on the coupling reagent^24–27^. To the best of our knowledge, the effect of the coupling reagent on α-C racemization of various amino acids has not been fully explored. Therefore, we pre-activated Fmoc-*l*-His(Trt)-OH, Fmoc-*l*-Ser(tBu)-OH, and Fmoc-*l*-Cys(Trt)-OH by treating them with various coupling reagents for 5 min at room temperature, and then we coupled them with *l*-Leu-OtBu (**25**) (Fig. 3a and Supplementary Figs. 2–4). Chiral chromatography revealed that the extent of α-C racemization in the obtained dipeptides generally increased in the order Fmoc-*l*-Ser(tBu)-OH <Fmoc-*l*-Cys(Trt)-OH <Fmoc-*l*-His(Trt)-OH (Fig. 3b). Racemization of Fmoc-*l*-Ser(tBu)-OH was negligible except when the coupling reagent was HATU/NMM (HATU = *N*-[(dimethylamino)-1*H*-1,2,3-triazolo[4,5-*b*]pyridino-1-ylmethylene]-*N*-methylmethanaminium hexafluorophosphate; NMM = *N*-methylmorpholine). For Fmoc-*l*-Cys(Trt)-OH, racemization occurred with all the coupling reagents except DIC/Oxyma (DIC = diisopropylcarbodiimide; Oxyma = ethyl 2-cyano-2-(hydroxyimino)acetate). Fmoc-*l*-His(Trt)-OH underwent racemization to afford 1.8% of *d*-product **31** even when the mildest coupling reagent, DIC/Oxyma, was employed. Moreover, the percentage of *d*-product increased to 31.0% when the coupling was carried out at 55 °C. These results suggest that avoiding α-C racemization during Fmoc SPPS is essentially impossible.

In contrast, no obvious α-C racemization was observed when DNPBS-protected Cys, His, or Ser was coupled with l-Leu-OtBu (**25**) under the same conditions, except in the case of DNPBS-*l*-His(Trt)-OH, which afforded a small amount of *d*-product **33** when EDCl/HOBt (EDCl = *N*-ethyl-*N*’-(3-dimethylaminopropyl)carbodiimide hydrochloride; HOBt = 1-hydroxybenzotriazole) was used as the coupling reagent. Taken together, our results indicate that DNPBS is superior to Fmoc in that α-C racemization can be completely suppressed during DNPBS SPPS as long as the proper coupling reagent is chosen.

### Peptide synthesis by means of DNPBS SPPS

**Fig. 4:**
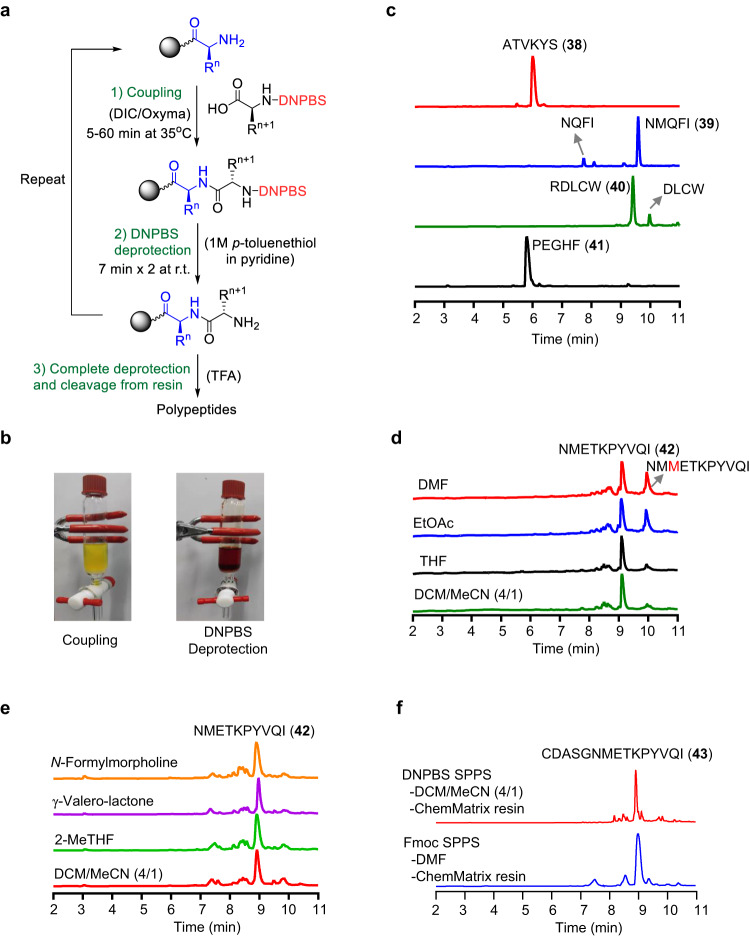
Synthesis of peptides by means of DNPBS solid-phase peptide synthesis (SPPS). **a** Steps of the DNPBS SPPS process. **b** Solution colors during the coupling step and the deprotection step. **c** UPLC-MS analysis of crude peptides **38**–**41**. **d**, **e** UPLC-MS analysis of crude peptide **42** synthesized in various coupling solvents on polystyrene resin (**d**) or ChemMatrix resin (**e**). **f** UPLC-MS analysis of crude peptide **43** synthesized by either DNPBS SPPS (top) or Fmoc SPPS (bottom). DNPBS, 2,4-dinitro-6-phenyl-benzene sulfenyl; Fmoc, 9-fluorenylmethoxycarbonyl.

Using a manual SPPS vessel and Rink amide aminomethyl polystyrene resin, we optimized the DNPBS SPPS process and synthesized peptides **38**–**41** on a 25 μM scale using DNPBS-protected amino acids **5**–**24**. Each DNPBS SPPS cycle comprises two steps: coupling and deprotection (Fig. 4a). We found that DIC/Oxyma was the best coupling reagent. Given that THF showed good compatibility with both the coupling reagent and the DNPBS-amino acids, it was used as the coupling solvent^9,28^. Using 3 equiv. of each amino acid and shaking for 30 min at 35 °C ensured quantitative coupling for most of the amino acids. DNPBS-*l*-Arg(Pbf)-OH (**21**) showed the lowest coupling efficiency, so the coupling step was repeated to ensure a satisfactory yield. In contrast, coupling of DNPBS-*l*-His(Trt)-OH (**6**) was complete in 5 min; and if the coupling time was extended beyond 5 min, redundant His incorporation was observed, indicating that under the coupling conditions, **6** was less stable than the other DNPBS-protected amino acids. It may be possible to eliminate this problem by optimizing the side-chain protecting group on the His residue.

Removal of the DNPBS group from the resin-bound peptide was much slower than removal from the free peptide in solution. Therefore, two treatments (7 min each) with 1 M *p*-toluenethiol/pyridine were carried out to ensure complete removal of DNPBS. Notably, the disulfane generated from the removed protecting group was red in pyridine solution, so we could monitor the deprotection process visually (Fig. 4b).

Once the peptide was assembled, the peptide chain was cleaved from the resin by treatment with 95% TFA containing 2.5% water and 2.5% triisopropylsilane at room temperature for 1 h. Ultra-high-performance liquid chromatography–mass spectrometry (UPLC-MS) of crude products **38**–**41** showed that their purity exceeded 80% (Fig. 4c). The observed side products were ascribed to sequence deletion and could be easily removed by routine purification methods.

Next, we synthesized a longer peptide (**42**) in various coupling solvents. We found that the overall yield of **42** in highly polar solvents (i.e., DMF and EtOAc) was markedly lower than that in THF (Fig. 4d). We attributed this mainly to redundant Met incorporation in the former solvents, suggesting that DNPBS-protected Met was less stable in polar solvents than in THF. Although our DNPBS-protected amino acids showed good solubility in DCM, this relatively nonpolar solvent was unsuitable for the coupling reaction because DIC/Oxyma was insufficiently soluble in DCM. Instead, we used 4:1 (v/v) DCM/MeCN, which was found to be a good option. Peptide synthesis in DCM/MeCN afforded **42** that was as pure as the product obtained in THF. Our results indicate that low-polarity solvents are superior to highly polar ones for DNPBS SPPS.

We found that during DNPBS SPPS, the coupling yield decreased dramatically when the peptide chain was longer than 10 residues, mainly because the swelling factor of the polystyrene resin decreased as the peptide chain length increased, especially in low-polarity solvents^5^. We thus turned to polyethylene glycol–based ChemMatrix resin, which swells well in a broad range of solvents and is suitable for the synthesis of long peptides^29^. Specifically, peptide **42** was synthesized on ChemMatrix resin with DCM/MeCN (4:1, v/v) as the coupling solvent. The swelling of the ChemMatrix resin was indeed much better than that of the polystyrene resin. However, the improved swelling did not lead to an obvious increase in synthetic yield. Utilizing a greener coupling solvent, such as 2-methyltetrahydrofuran^30^, γ-valerolactone, or *N*-formylmorpholine^31^, also failed to improve the synthetic yield (Fig. 4e).

Next, we tried to synthesize peptide **43** (15 residues) on ChemMatrix resin with 4:1 (v/v) DCM/MeCN as the coupling solvent. UPLC analysis of the obtained crude product revealed that the target peptide was obtained in 52% yield (Fig. 4f). In contrast, synthesis of the same sequence by means of Fmoc SPPS afforded a 65% yield. Taken together, these results indicate that DNPBS SPPS on ChemMatrix resin could be used for the synthesis long peptides but that this strategy is somewhat less efficient than standard Fmoc SPPS.

### Comparison of side reactions during DNPBS SPPS and conventional SPPS

**Fig. 5:**
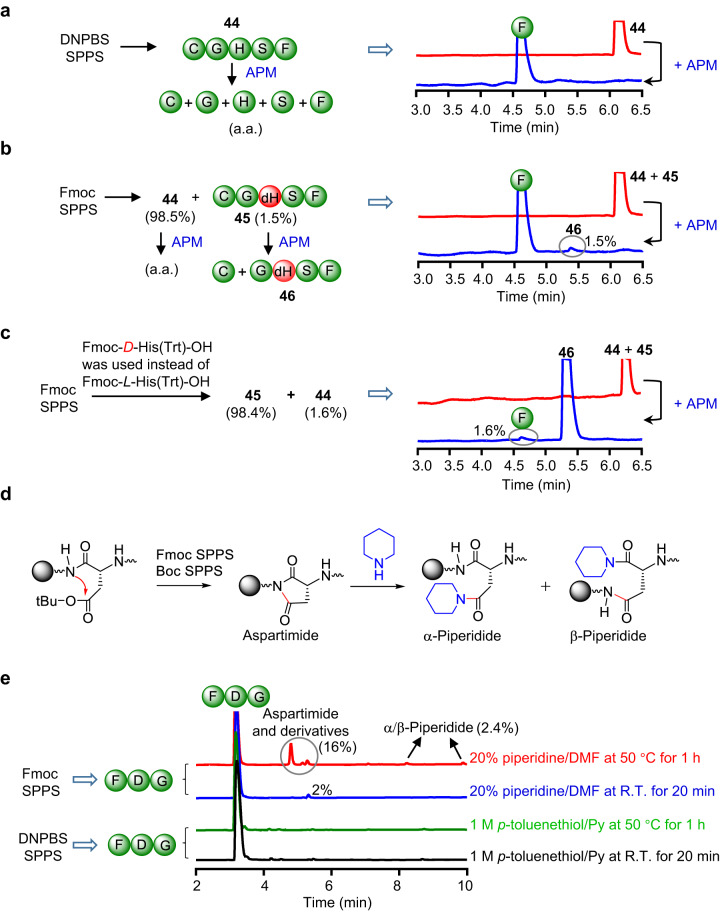
Comparison of side reactions during DNPBS solid-phase peptide synthesis (SPPS) and Fmoc SPPS. **a** UPLC-MS analysis of the products of APM (aminopeptidase M) digestion of peptide CGHSF (**44**) prepared by DNPBS SPPS. **b** UPLC-MS analysis of the products of APM digestion of **44** prepared by Fmoc SPPS. **c** UPLC-MS analysis of the product of APM digestion of authentic peptide CGdHSF (**45**) prepared by Fmoc SPPS. **d** Mechanism of aspartimide and piperidide formation during conventional SPPS. **e** UPLC-MS analyses of aspartimide and piperidide formation during the synthesis of tripeptide FDG by Fmoc SPPS and DNPBS SPPS. DNPBS, 2,4-dinitro-6-phenyl-benzene sulfenyl; Fmoc, 9-fluorenylmethoxycarbonyl.

The most deleterious side products of Fmoc SPPS arise from racemization at the α-C of Ser, Cys, and His. We have shown that coupling of DNPBS-protected amino acids with a free α-amino group exhibited greatly decreased α-C racemization compared with that observed for Fmoc-protected amino acids. To determine whether DNPBS SPPS could afford peptides with no α-C racemization at all, we synthesized peptide CGHSF (**44**) using optimized DNPBS SPPS conditions. The product was separated and digested with aminopeptidase M (APM), which hydrolyses *l*-amino acids starting from the *N*-terminal of peptides^32,33^. UPLC-MS analysis of the digestion mixture showed that **44** had been completely degraded into amino acid monomers (Fig. 5a). In contrast, digestion of **44** prepared by means of Fmoc SPPS (coupling with DIC/Oxyma at 35 °C in DMF) generated a side product (1.5%) in addition to the amino acid monomers (Fig. 5b). The side product had a [MH]^+^ value of 446.1871, suggesting that it was peptide **46**, which could have been produced by APM digestion of peptide **45**. To confirm this possibility, we synthesized authentic **45** by using Fmoc-*d*-His(Trt)-OH instead of natural Fmoc-*l*-His(Trt)-OH, and we found that **46** was indeed the predominant product of APM digestion of **45** (Fig. 5c). We also observed a small amount (1.6%) of free amino acid monomer, once again confirming that racemization at the α-C of His during Fmoc SPPS is difficult to avoid. Although under the above-mentioned mild conditions, α-C racemization during Fmoc SPPS was observed only for His and only to a limited extent (1.5%), racemization may become problematic when a polypeptide contains multiple His residues or when the synthesis is carried out at elevated temperature. In contrast, no racemization was observed during DNPBS SPPS.

Another problematic side reaction of both Fmoc and Boc SPPS is aspartimide formation at Asp residues during α-amino deprotection (Fig. 5d), especially when Asp is at the *N*-terminal of a Gly, Asn, Ala, or Gln residue^11,12^. These cyclic products can be transformed to acyclic products via hydrolysis and aminolysis (e.g., affording α- and β-piperidide during treatment with piperidine)^34^. To determine whether the use of DNPBS SPPS could prevent the formation of aspartimide and derivatives, we synthesized tripeptide FDG by means of DNPBS SPPS and subjected it to deprotection. We found that neither normal deprotection conditions (1 M *p*-toluenethiol/pyridine at room temperature for 20 min) nor harsh deprotection conditions (1 M *p*-toluenethiol/pyridine at 50 °C for 1 h) resulted in aspartimide formation (Fig. 5e). In contrast, synthesis of the same tripeptide by means of Fmoc SPPS and deprotection with 20% piperidine/DMF at room temperature for 20 min resulted in a 2% yield of an aspartimide. Increasing the deprotection temperature and time (20% piperidine/DMF at 50 °C for 1 h) dramatically enhanced the yield of side products.

Because the conditions used for removing DNPBS (1 M *p*-toluenethiol in pyridine) are much milder than the conditions for removing Fmoc (20% piperidine in DMF), aspartimide formation was completely precluded during DNPBS SPPS. Given that most of the side reactions observed during Fmoc SPPS are ascribed to the basic conditions used for Fmoc deprotection^10^, the use of DNPBS SPPS would avoid many of these side reactions.

### Synthesis of peptides by a combination of DNPBS SPPS and Fmoc SPPS

**Fig. 6:**
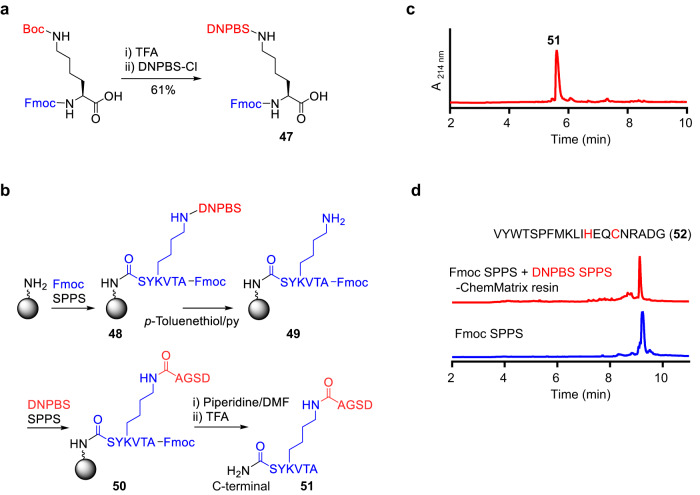
Synthesis of long and complex peptides by a combination of DNPBS and Fmoc solid-phase peptide synthesis (SPPS). **a**–**c** Synthesis and UPLC analysis of branched peptide **51**. **d** UPLC analysis of crude peptide **52** synthesized by a combination of DNPBS SPPS and Fmoc SPPS (top) or by Fmoc SPPS alone (bottom). DNPBS, 2,4-dinitro-6-phenyl-benzene sulfenyl; Fmoc, 9-fluorenylmethoxycarbonyl.

Although using DNPBS SPPS avoids the major side reactions associated with conventional SPPS, the former is less efficient than the latter for the synthesis of long peptides, as mentioned above. We therefore wondered whether it would be possible to take advantage of the merits of each method by combining them for the synthesis of complex peptides and long peptides. To explore this possibility, we synthesized a branched peptide^35,36^. Specifically, we first synthesized Fmoc-*l*-Lys(DNPBS)-OH (**47**, 61% yield) from Fmoc-*l*-Lys(Boc)-OH (Fig. 6a) and then used **47** to synthesize hexapeptide **48** by means of Fmoc SPPS (Fig. 6b). Subsequent removal of the DNPBS group on the Lys side chain with 1 M *p*-toluenethiol/pyridine was followed by chain elongation from the ε-amine of Lys by means of DNPBS SPPS to give **50**. Finally, the *N*-terminal Fmoc group of **50** was removed by treatment with piperidine/DMF, and subsequent reaction with TFA afforded target branched peptide **51**. UPLC analysis of the crude product revealed that its purity was nearly 90% (Fig. 6c), and its structure was confirmed by MS/MS analysis (Supplementary Fig. 5).

Next, we synthesized a model 20 mer peptide **52** containing all 20 natural amino acids^37^ on ChemMatrix resin by combining DNPBS SPPS and Fmoc SPPS (Fig. 6d). The histidine and cysteine residues in the sequence were incorporated by means of DNPBS SPPS, and all the other residues were incorporated by means of standard Fmoc SPPS. UPLC analysis of the crude product showed that the combined strategy afforded the target product in 42% yield (Fig. 6d). Of note, there were no impurities that co-elute with the main product. In contrast, synthesis of **52** based on Fmoc SPPS produced the aim product together with some co-eluting impurities (Fig. 6d), making it hard to obtain pure peptide by routine purification methods. Therefore, in this regard, the combined strategy can afford cleaner final products than Fmoc SPPS.

The above-described results suggest that Fmoc and DNPBS are orthogonal amino protecting groups. Given that DNPBS SPPS and Fmoc SPPS employ the same side-chain-protection strategies, resins, coupling reagents, and postsynthetic treatments, the combination of DNPBS SPPS and Fmoc SPPS is advantageous for synthesizing peptides that would be difficult to synthesize by means of conventional SPPS^38^.

## Discussion

In this study, we developed a reagent (DNPBS-Cl, **2**) for protection of amino groups and demonstrated its application in SPPS. DNPBS-Cl can be easily prepared and introduced to the α-amino group of amino acids and the ε-amino group of Lys in high yields. Unlike conventional α-amino protecting groups (e.g., Boc and Fmoc), which require strong acid or base for removal, DNPBS can be efficiently removed at room temperature in minutes under nearly neutral conditions (1 M *p*-toluenethiol/pyridine). Using DNPBS as a protecting group, we developed a method for SPPS, which we refer to as DNPBS SPPS. Compared with conventional SPPS, DNPBS SPPS has three obvious merits. First, coupling of DNPBS-protected amino acids with the *N*-terminal α-amino group of a polypeptide chain does not lead to α-C racemization and thence to the generation of diastereomeric impurities that are difficult to remove. Second, the conditions for DNPBS removal are mild enough to greatly suppress the main side reactions (e.g., aspartimide formation) that occur under the strongly acidic and basic deprotection conditions used in conventional SPPS. Third, removing DNPBS with 1 M *p*-toluenethiol in pyridine yields a red product (λ_max_ = 490 nm), so the progress of the deprotection reaction and each coupling yield can be monitored visually or by UV–vis spectroscopy.

Notably, however, when polystyrene resin was used, DNPBS SPPS could be employed only for the synthesis of peptides with less than 10 residues. In contrast, when polyethylene glycol–based ChemMatrix resin was used, longer peptides could be synthesized, but the coupling efficiency was not comparable to that of standard Fmoc SPPS. Fortunately, the orthogonality of the DNPBS and Fmoc protecting groups allowed DNPBS SPPS and Fmoc SPPS to be combined for the synthesis of peptides that would be difficult to prepare by either method alone. Moreover, the good solubility of DNPBS-protected amino acids in a broad range of organic solvents might make the use of DNPBS more attractive for liquid-phase peptide synthesis (LPPS) than for SPPS. Development of DNPBS-based LPPS is underway in our laboratory.

## Methods

### Synthesis of DNPBS-Cl (2)

Benzyl mercaptan (37.1 mL, 315.7 mmol) and Et_3_N (59.8 mL, 430.5 mmol) were added to a solution of 2-chloro-3,5-dinitro-biphenyl (80 g, 287 mmol) in CH_3_OH (200 mL). The reaction mixture was heated at reflux (80 °C) for 8 h under an argon atmosphere. After the mixture was cooled to −20 °C, the precipitate that formed was collected by filtration and washed with cooled CH_3_OH (−20 °C) and petroleum ether to give **1** (98.8 g, 94%) as a yellow solid. To a solution of **1** (98.8 g, 269.8 mmol) in 1,2-dichloroethane (200 mL) was added sulfuryl chloride (24 mL, 296.8 mmol). After stirring at room temperature for 2 h under an argon atmosphere, the reaction mixture was concentrated under a vacuum to a quarter of the original volume, and petroleum ether (300 mL) was added to the residue. The resulting precipitate was collected by filtration, washed with cooled petroleum ether, and dried under a vacuum to give **2** (71.3 g, 85%). ^1^H NMR (400 MHz, CDCl3) δ: 8.81 (d, *J* = 2.4 Hz, 1H, -ArH), 8.42 (d, *J* = 2.4 Hz, 1H, -ArH), 7.58 – 7.52 (m, 5H, -ArH). ^13^C NMR (100.6 MHz, CDCl_3_) δ: 150.2, 147.2, 146.6, 140.4, 137.5, 130.2, 129.7, 129.3, 128.4, 118.5.

### General procedure for synthesis of DNPBS-protected amino acids

4-Methylmorpholine (4 or 8 equiv.) and trimethylsilyl chloride (2 or 4 equiv.) were successively added to a magnetically stirred suspension of H-*l*-AA-OH (1 or 1.2 equiv., AA = amino acid) in anhydrous 1:1 (v/v) DCM/MeCN (30 mL). The turbid reaction mixture was stirred at 70 °C under an argon atmosphere until it became transparent (1 h). After the solution cooled to room temperature, DNPBS-Cl (6 mmol, 1 equiv.) in anhydrous DCM (15 mL) was added dropwise, and the solution was stirred for 20 min at room temperature. The reaction was quenched with CH_3_OH (1 mL) and concentrated under a vacuum. The residue was partitioned between EtOAc (40 mL) and aqueous citric acid (2%, 40 mL). The organic layer was separated, washed once with 2% aqueous citric acid (40 mL) and twice with saturated aqueous NaCl (30 mL), dried over sodium sulfate, and then filtered; and the filtrate was concentrated under a vacuum. The residue was dissolved in EtOAc or DCM. Pure DNPBS-protected amino acids were obtained by precipitation from petroleum ether and/or Et_2_O.

### DNPBS SPPS

Peptide sequences were assembled on Rink amide aminomethyl polystyrene resin (loading 0.338 mmol/g) or H-Rink amide ChemMatrix resin (loading 0.4–0.6 mmol/g) in a manual SPPS vessel (5 mL). All compositions are reported as % volume unless specified otherwise. The resin (74 mg, 0.025 mmol) was swelled in anhydrous DCM (2 mL) for 30 min. After draining, the resin was washed with DCM (3 × 5 mL) and subjected to Fmoc-removal conditions, and then DNPBS-protected amino acids were incorporated by means of the following two-step procedure:

#### Step 1 (coupling)

DNPBS–*l*-AA-OH (3 equiv., 0.075 mmol) in 1.5 mL of anhydrous THF (for polystyrene resin) or 4:1 (v/v) DCM/MeCN (for ChemMatrix resin) was mixed with Oxyma (0.075 mmol) and DIC (0.075 mmol); and the mixture was added to the peptidyl resin. The reaction temperature was kept at 35 °C for 30 min with continuous vortexing at 30 rpm. The resin was drained and then washed with DMF (3 × 5 mL) and DCM (3 × 5 mL). For His, the coupling time was always limited to less than 5 min. For Arg, two 30 min coupling periods were used.

#### Step 2 (DNPBS removal)

Deprotection solution (2 mL of 1 M *p*-toluenethiol in pyridine) was used to remove DNPBS (7 min at room temperature) with continuous vortexing at 30 rpm. After draining, the deprotecting was repeat once again (total 2 × 7 min). The resin was washed with pyridine (2 × 5 mL), DMF (2 × 5 mL) and DCM (2 × 5 mL).

#### Postsynthetic treatment

The peptidyl resin was washed with DMF (3 × 5 mL) and then with DCM (3 × 2 mL) and dried under a continuous stream of argon. The peptide was released from the resin with 95 vol% TFA containing 2.5% water and 2.5% triisopropylsilane (50 uL of TFA solution per milligram of peptidyl resin) at room temperature for 1 h with continuous vortexing at 900 rpm. The resin was removed by filtration and washed with TFA. All filtrates were combined and concentrated under a vacuum to nearly a fifth of the initial volume, and the peptide was precipitated from Et_2_O. The peptide pellet was collected by centrifugation, dried under an argon flow, and stored at −20 °C until characterization. All peptide samples were analysed on a UPLC-MS instrument equipped with a reverse-phase C18 column (ACQUITY UPLC® BEH C18, 100 × 2.1 mm, 1.7 μm). UPLC conditions: column temperature, 35 °C; solvent, linear gradient from 2 to 40 vol% MeCN in water containing 0.1 vol% TFA over 15 min; flow rate, 0.4 mL/min. The detection wavelength was 210 nm unless specified otherwise.

### Branched peptide synthesis

Peptide ATVKYS was synthesized on Rink amide aminomethyl polystyrene resin (loading 0.338 mmol/g) on a 0.025 mmol scale by means of standard Fmoc SPPS except that Fmoc-*l*-Lys(DNPBS)-OH (**47**) dissolved in 4:1 (v/v) DCM/MeCN was used for Lys incorporation. The final Fmoc-removal step was omitted, so the *N*-terminal amino function was protected by Fmoc. Then the resin was treated with 1 M *p*-toluenethiol in pyridine (2 mL) at room temperature twice (7 min each time) to remove the DNPBS group on the Lys side chain. After the resin was washed with DMF (3 × 5 mL) and DCM (3 × 5 mL), chain elongation from ε-amino group of Lys was carried out by means of standard DNPBS SPPS with 4:1 (v/v) DCM/MeCN as the coupling solvent. Finally, the *N*-terminal Fmoc group on the Ala residue was removed by treatment with 2 mL of 20% piperidine in DMF at room temperature twice (10 min each time). After postsynthetic treatment, the obtained crude product was analysed by UPLC-MS. UPLC conditions: C18 column (ACQUITY UPLC® BEH C18, 100 × 2.1 mm, 1.7 μm); column temperature, 35 °C; solvent, linear gradient from 2 to 40 vol% MeCN in water containing 0.1 vol% TFA over 15 min; flow rate, 0.4 mL/min. The detection wavelength was 210 nm.

### Combining Fmoc SPPS and DNPBS SPPS for synthesis of long peptides

Long peptides were synthesized on H-Rink amide ChemMatrix resin at a 0.025 mmol scale. After resin swelling, DNPBS–*l*-AA-OH (0.075 mmol) in 1.5 mL of anhydrous 4:1 (v/v) DCM/MeCN or Fmoc–*l*-AA-OH (0.075 mmol) in 1.5 mL of DMF was mixed with Oxyma (0.075 mmol) and DIC (0.075 mmol); and the mixture was then added to the peptidyl resin. The coupling reaction was allowed to proceed at 35 °C for 30–120 min with continuous vortexing at 30 rpm. For DNPBS–*l*-His-OH, the coupling time was limited to 5 min. The resin was drained and then washed with DMF (3 × 5 mL) and DCM (3 × 5 mL).

The Fmoc group was removed by incubation twice with 2 mL of 20% piperidine in DMF for 10 min at room temperature with continuous vortexing at 30 rpm; 2 mL of 1 M *p*-toluenethiol in pyridine was used to remove DNPBS from the α-amino group for 10 min at room temperature with continuous vortexing at 30 rpm. After draining, the deprotecting was repeat once again (total 2 × 10 min). The resin was washed with pyridine (2 × 5 mL), DMF (2 × 5 mL), and DCM (2 × 5 mL) in that order.

### Supplementary information

Supplementary Information

Peer review file

## Data Availability

Methods and all relevant data are available in Supplementary Information and from the authors.
