# Supplementary material for: Suppression of alpha-carbon racemization in peptide synthesis based on a thiol-labile amino protecting group
Source: Nat Commun. 2023 Sep 1;14:5324. doi: 10.1038/s41467-023-41115-x (PMC10474026; doi:10.1038/s41467-023-41115-x)

Supplementary Information

# Suppression of Alpha-Carbon Racemization in Peptide Synthesis Based on a Thiol-Labile Amino Protecting Group

Yifei Zhou, Hongjun Li, Yi Huang, Jiahui Li, Guiyu Deng, Gong Chen, Zhen Xi and Chuanzheng Zhou*

[a] State Key Laboratory of Elemento-Organic Chemistry, Frontiers Science Center for New Organic Matter, Department of Chemical Biology, College of Chemistry, Nankai University, Tianjin 300071, China

* chuanzheng.zhou@nankai.edu.cn

**This PDF file includes:**

Supplementary Materials and Methods (S2)
Supplementary Figures (S19)
NMR spectra of synthesized new compounds (S24)
High-resolution mass spectral (HRMS) of new compounds (S55)

## Supplementary Materials and Methods

**General method.** Unless noted otherwise, all chemicals were obtained from commercial supplier and used without further purification. Rink amide aminomethyl polystyrene resin (loading 0.338 mmol/g) for solid-phase peptide synthesis was purchased from GL Biochem (shanghai) Ltd and H-Rink amide ChemMatrix resin (loading 0.4-0.6 mmol/g) was purchased from Sigma. THF was dried by distillation over sodium. $CH_3CN$ and DCM was dried by distillation over $CaH_2$. Other completely dry solvents were obtained from J&K Chemical and used without further purification. $^1H$ and $^{13}C$ NMR spectra were obtained from a Bruker ASCEND 400 MHz instrument. $^1H$ NMR data are reported as follows: chemical shift (δ ppm), multiplicity (s = singlet, brs = broad singlet, d = doublet, t = triplet, q = quartet, m = multiplet, dd = doublet of doublet, dt = doublet of triplet, ddd = doublet of doublet of doublet), coupling constant (Hz), and integration. $^{13}C$ NMR data are reported as chemical shift relative to $CDCl_3$ (77.16 ppm). Analytical thin-layer chromatography (TLC) was carried out on SiLiDa silica gel GF254 plates. Column chromatography was carried out on silica gel (300-400 mesh), thin-layer chromatography plate (thickness 1 mm) and aluminum oxide (300-400 mesh). HPLC was conducted on Waters HP Series fitted with Ultimate® XB-C18 (10×250 mm, 5 μm). High resolution mass spectra (HRMS) were recorded on Waters Xevo G2-XS Q-TOF mass spectrometry. Chiral chromatography analysis was performed on Thermo Scientific Dionex UltiMate 3000 Standard Systems using chiral column with hexane and isopropanol as eluent.

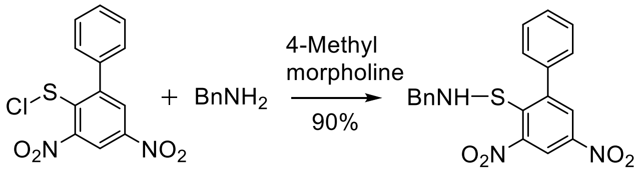

DNPBS-Cl (**2**) **3**

**Synthesis of 3**. **2** (1.49 g, 4.8 mmol) in anhydrous DCM (20 mL) was added dropwise slowly to a mixture of benzylamine (436 μL, 4 mmol) and 4-methylmorpholine (880 μL, 8 mmol) in 3 mL anhydrous DCM. After stirring at room temperature for 20 min under an argon atmosphere, the reaction mixture was quenched with saturated aqueous NaCl (3 mL). The organic layer was separated and dried over sodium sulfate, and concentrated on vacuum. The residue was purified on a silica gel column (10% EtOAc in PE, v%) to give **3** (1.37 g, 90%) as a yellow solid. $R_f$ = 0.61 (10% EtOAc in PE, v%). $^1H$ NMR (400 MHz, $CDCl_3$) δ: 8.71 (d, $J$ = 2.4 Hz, 1H, -ArH), 8.21 (d, $J$ = 2.4 Hz, 1H, -ArH), 7.59 – 7.57 (m, 2H, -ArH), 7.51 – 7.45 (m, 3H, -ArH), 7.19 – 7.18 (m, 3H, -ArH), 6.96 – 6.94 (m, 2H, -ArH), 3.57 (d, $J$ = 5.5 Hz, 2H, $-CH_2$), 2.26 (t, $J$ = 5.4 Hz, 1H, -NH). $^{13}C$ NMR (100.6 MHz, $CDCl_3$) δ: 149.7, 146.4, 145.3, 143.9, 137.4, 137.2, 129.6, 129.5, 128.4, 128.3, 127.9, 127.8, 119.2, 56.3. HRMS (ESI-Q-TOF) m/z: $[M+H]^+$ calcd for $C_{19}H_{16}N_3O_4S$ 382.0856; found 382.0880.

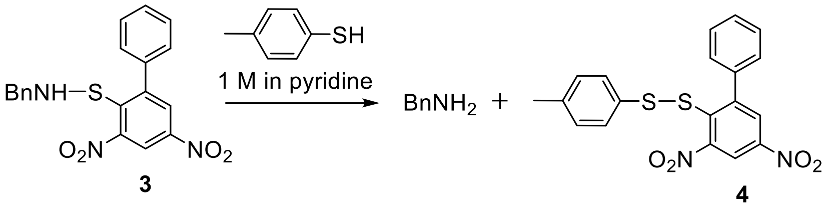


**Synthesis of 4**. (a) To a solution of **3** (1.55 g, 5 mmol) in 20 mL anhydrous DCM was added *p*-toluenethiol (621 mg, 5 mmol). After stirring at room temperature for 30 min under an argon atmosphere, the reaction mixture was concentrated and the residue was purified by column chromatography on silica gel (6.7% EtOAc in PE, v%) to give **4** (1.89 g, 95%). (b) Compound **3** (10 mg, 0.03 mmol) was dissolved in 1 M *p*-toluenethiol in pyridine (1 mL) and stirred at room temperature for 1 min. The reaction mixture was concentrated on vacuum. The residue was dissolved in 1 mL DCM and then purified by a thin-layer chromatography plate (eluent: 10% EtOAc in PE, v%) to give **4** as a yellow solid. $R_f$ = 0.48 (6% EtOAc in PE, v%). $^1$H NMR (400 MHz, $CDCl_3$) δ: 8.49 (d, $J$ = 2.3 Hz, 1H, -ArH), 8.28 (d, $J$ = 2.3 Hz, 1H, -ArH), 7.48 – 7.42 (m, 3H, -ArH), 7.35 – 7.28 (m, 2H, -ArH), 7.02 – 6.97 (m, 4H, -ArH), 2.29 (s, 3H, $-CH_3$). $^{13}$C NMR (100.6 MHz, $CDCl_3$) δ: 153.1, 149.3, 146.5, 139.4, 138.6, 137.5, 131.6, 131.0, 130.0, 129.4, 129.2, 128.7, 127.8, 118.4, 21.2. HRMS (ESI-Q-TOF) m/z: $[M+H]^+$ calcd for $C_{19}H_{15}N_2O_4S_2$ 399.0468; found 399.0461.

**Preparation of DNPBS-protected amino acid building blocks**

Side chains of amino acids were protected as follows: tert-butyloxycarbonyl (Boc) for Lys and Trp; tert-butyl ester for Ser, Thr, Tyr, Asp, Glu; trityl (Trt) for Cys, His, Asn, Gln; 2,2,4,6,7-Pentamethyl-2,3-dihydrobenzofuran-5-sulfonyl (Pbf) for Arg. Side chain-protected amino acids were reacted with DNPBS-Cl to afford DNPBS-protected building blocks according to the general procedure.

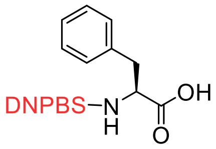


DNPBS-*L*-Phe-OH (**5**)
83%

**Synthesis of DNPBS-*L*-Phe-OH (5)**. **5** was synthesized on 6 mmol scale according to the general procedure. H-*L*-Phe-OH: 4-methylmorpholine : TMSCl : DNPBS-Cl = 7.2 mmol : 28.8 mmol : 14.4 mmol : 6 mmol. The crude product was dissolved in the minimum volume of $Et_2O$, followed by precipitation from petrol ether to give **5** (2.19 g, 83%) as a yellow solid. $R_f$ = 0.81 (50% EtOAc in PE, with 1% $CH_3COOH$, v/v). $^1$H NMR (400 MHz, DMSO) δ: 12.71 (brs, 1H, -COOH), 8.51 (d, $J$ = 2.3 Hz, 1H, -ArH), 8.12 (d, $J$ = 2.4 Hz, 1H, -ArH), 7.57 – 7.50 (m, 3H, -ArH), 7.46 – 7.44 (m, 2H, -ArH), 7.19 – 7.11 (m, 3H, -ArH), 6.96 – 6.95 (m, 2H, -ArH), 4.30 (d, $J$ = 7.1 Hz, 1H, -CH), 3.49 (dd, $J$ = 6.8 Hz, $J$ = 13.6 Hz, 1H, -NH), 2.77 (q, $J$ = 6.5 Hz, 1H, $-CH_2$), 2.67 (q, $J$ = 7.1 Hz, 1H, $-CH_2$). $^{13}$C NMR (100.6 MHz, DMSO) δ: 173.9, 149.2, 145.2, 144.9,

143.4, 137.2, 137.1, 129.7, 129.3, 129.2, 128.5, 126.7, 119.9, 119.8, 64.3, 37.3. HRMS (ESI-Q-TOF) m/z: $[M+Na]^+$ calcd for $C_{21}H_{17}N_3NaO_6S$ 462.0730; found, 462.0775.

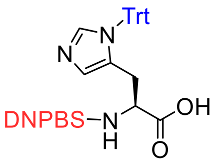


DNPBS-*L*-His(Trt)-OH (**6**)
75%

**Syethesis of DNPBS-*L*-His(Trt)-OH (6)**. **6** was synthesized on 6 mmol scale according to the general procedure. H-*L*-His(Trt)-OH: 4-methylmorpholine : TMSCl : DNPBS-Cl = 6 mmol : 48 mmol : 24 mmol : 6 mmol. After quenching with $CH_3OH$ and concentration on vacuum, the left residue was partitioned between EtOAc (40 mL) and 0.5% aqueous solution of citric acid (40 mL). The organic layer was washed with 0.5% aqueous solution of citric acid (40 mL) once, then saturated aqueous NaCl (30 mL) twice. The organic layer was dried over sodium sulfate, filtered and concentrated on vacuum. The residue was dissolved in the minimum volume of DCM (5 mL), followed by precipitation from $Et_2O$ to give **6** (3.02 g, 75%) as a yellow solid. $R_f$ = 0.78 (50% EtOAc in PE, with 1% $CH_3COOH$, v/v). $^1H$ NMR (400 MHz, $CDCl_3$) δ: 14.57 (brs, 1H, -COOH), 8.53 (d, $J$ = 2.2 Hz, 1H, -ArH), 8.18 (d, $J$ = 2.4 Hz, 1H, -ArH), 7.56 (s, 1H), 7.42 – 7.32 (m, 15H, -ArH), 7.13 – 7.11 (m, 5H, -ArH), 6.64 (s, 1H), 3.19 (m, 1H), 3.07 – 2.97 (m, 2H), 2.78 (dd, $J$ = 6.2 Hz, $J$ = 14.6 Hz, 1H). $^{13}C$ NMR (100.6 MHz, $CDCl_3$) δ: 173.5, 149.3, 145.9, 144.9, 143.9, 141.7, 137.7, 137.1, 133.4, 129.7, 129.3, 129.2, 128.4, 128.3, 127.6, 121.0, 119.0, 76.2, 62.0, 29.2. HRMS (ESI-Q-TOF) m/z: $[M+Na]^+$ calcd for $C_{37}H_{29}N_5NaO_6S$ 694.1731; found, 694.1887.

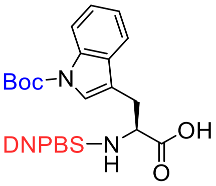


DNPBS-*L*-Trp(Boc)-OH (**7**)
77%

**Synthesis of DNPBS-*L*-Trp(Boc)-OH (7)**. **7** was synthesized on 6 mmol scale according to the general procedure. H-*L*-Trp(Boc)-OH: 4-methylmorpholine : TMSCl : DNPBS-Cl = 6 mmol : 24 mmol : 12 mmol : 6 mmol. The crude product was dissolved in 5 mL EtOAc, followed by precipitation from $Et_2O$ to give **7** (2.67 g, 77%) as a yellow solid. $R_f$ = 0.64 (25% EtOAc in PE, with 1% $CH_3COOH$, v/v). $^1H$ NMR (400 MHz, DMSO-*d6*) δ: 8.14 (s, 1H, -ArH), 7.85 – 7.84 (m, 1H, -ArH), 7.79 – 7.78 (m, 1H, -ArH), 7.54 – 7.47 (m, 3H, -ArH), 7.33 – 7.27 (m, 3H, -ArH), 7.19 – 7.14 (m, 3H, -ArH), 4.65 (d, $J$ = 5.6 Hz, 1H), 3.79 (m, 1H), 2.79 (d, $J$ = 7.0 Hz, 2H), 1.66 (s, 9H, $-(CH_3)_3$). $^{13}C$ NMR (100.6 MHz, DMSO-*d6*) δ: 174.4, 149.4, 148.7, 145.8, 144.3, 143.0, 136.9, 134.8, 130.2, 129.8, 129.4, 129.3, 126.2, 124.5, 124.2, 122.6, 119.5,

119.1, 115.9, 114.9, 83.9, 28.2. HRMS (ESI-Q-TOF) m/z: $[M+Na]^+$ calcd for $C_{28}H_{26}N_4NaO_8S$ 601.1364; found, 601.1416.

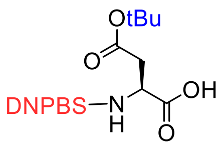


DNPBS-*L*-Asp(tBu)-OH (**8**)
86%

**Synthesis of DNPBS-*L*-Asp(tBu)-OH (8)**. **8** was synthesized on 6 mmol scale according to the general procedure. H-*L*-Asp(tBu)-OH: 4-methylmorpholine : TMSCl : DNPBS-Cl = 6 mmol : 24 mmol : 12 mmol : 6 mmol. The crude product was dissolved in 5 mL EtOAc, followed by precipitation from $Et_2O$ and petrol ether (1/9, v/v) to give **8** (2.40 g, 86%) as a yellow solid. $R_f = 0.76$ (6% $CH_3OH$ in DCM, with 1% $CH_3COOH$, v/v). $^1H$ NMR (400 MHz, $CDCl_3$) δ: 8.58 (d, $J = 2.4$ Hz, 1H, -ArH), 8.22 (d, $J = 2.4$ Hz, 1H, -ArH), 7.52 – 7.44 (m, 5H, -ArH), 3.47 – 3.42 (m, 1H), 3.31 (d, $J = 7.8$ Hz, 1H), 2.63 (dd, $J = 5.0$ Hz, $J = 17.2$ Hz, 1H), 2.55 (dd, $J = 5.0$ Hz, $J = 17.1$ Hz, 1H), 1.38 (s, 9H, $-(CH_3)_3$). $^{13}C$ NMR (100.6 MHz, $CDCl_3$) δ: 177.3, 169.5, 149.3, 145.2, 145.2, 144.2, 137.1, 129.5, 129.3, 128.5, 127.6, 119.1, 82.2, 60.3, 37.6, 27.9. HRMS (ESI-Q-TOF) m/z: $[M+Na]^+$ calcd for $C_{20}H_{21}N_3NaO_8S$ 486.0942; found, 486.0988.

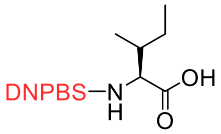


DNPBS-*L*-Ile-OH (**9**)
92%

**Synthesis of DNPBS-*L*-Ile-OH (9)**. **9** was synthesized on 6 mmol scale according to the general procedure. H-*L*-Ile-OH: 4-methylmorpholine : TMSCl : DNPBS-Cl = 7.2 mmol : 28.8 mmol : 14.4 mmol : 6 mmol. The crude product was dissolved in 5 mL EtOAc, followed by precipitation from $Et_2O$ and PE (1/9, v/v) to give **9** (2.17 g, 92%) as a yellow solid. $R_f = 0.81$ (6% $CH_3OH$ in DCM, with 1% $CH_3COOH$, v/v). $^1H$ NMR (400 MHz, $CDCl_3$) δ: 9.76 (brs, 1H, -COOH), 8.60 (d, $J = 2.4$ Hz, 1H, -ArH), 8.23 (d, $J = 2.4$ Hz, 1H, -ArH), 7.50 – 7.46 (m, 5H, -ArH), 3.13 (dd, $J = 4.6$ Hz, $J = 7.7$ Hz, 1H), 2.76 (d, $J = 7.8$ Hz, 1H), 1.65 – 1.55 (m, 1H), 1.28 – 1.18 (m, 1H), 1.07 – 0.96 (m, 1H), 0.78 (t, $J = 7.3$ Hz, 3H), 0.73 (d, $J = 6.9$ Hz, 3H). $^{13}C$ NMR (100.6 MHz, $CDCl_3$) δ: 178.4, 149.6, 145.5, 145.1, 144.7, 137.4, 129.4, 129.2, 128.5, 127.9, 119.1, 68.6, 37.9, 25.1, 15.1, 11.6. HRMS (ESI-Q-TOF) m/z: $[M+H]^+$ calcd for $C_{18}H_{20}N_3O_6S$ 406.1067; found, 406.1063.

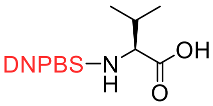


DNPBS-*L*-Val-OH (**10**)
81%

**Synthesis of DNPBS-*L*-Val-OH (10)**. **10** was synthesized on 6 mmol scale according to the general procedure. H-*L*-Val-OH: 4-methylmorpholine : TMSCl : DNPBS-Cl = 7.2 mmol : 28.8 mmol : 14.4 mmol : 6 mmol. The crude product was dissolved in 5 mL EtOAc, followed by precipitation from $Et_2O$ and PE (1/9, v/v) to give **10** (1.90 g, 81%) as a yellow solid. $R_f$ = 0.85 (50% EtOAc in PE, with 1% $CH_3COOH$, v/v). $^1H$ NMR (400 MHz, $CDCl_3$) δ: 8.61 (d, *J* = 2.4 Hz, 1H, -ArH), 8.23 (d, *J* = 2.4 Hz, 1H, -ArH), 7.49 (m, 5H, -ArH), 3.08 (dd, *J* = 4.8 Hz, *J* = 8.1 Hz, 1H), 2.77 (d, *J* = 8.1 Hz, 1H), 1.91 – 1.83 (m, 1H), 0.78 – 0.73 (m, 6H). $^{13}C$ NMR (100.6 MHz, $CDCl_3$) δ: 178.1, 149.6, 145.5, 145.2, 144.8, 137.4, 129.4, 129.2, 128.5, 127.8, 119.1, 70.0, 31.3, 18.5, 17.7. HRMS (ESI-Q-TOF) m/z: $[M+Na]^+$ calcd for $C_{17}H_{17}N_3NaO_6S$ 414.0730; found, 414.0702.

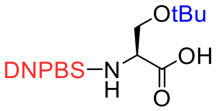


DNPBS-*L*-Ser(tBu)-OH (**11**)
82%

**Synthesis of DNPBS-*L*-Ser($^t$Bu)-OH (11)**. **11** was synthesized on 6 mmol scale according to the general procedure. H-*L*-Ser(tBu)-OH: 4-methylmorpholine : TMSCl : DNPBS-Cl = 6 mmol : 24 mmol : 12 mmol : 6 mmol. The crude product was recrystallized from $CH_3OH$ to give **11** (2.14 g, 82%) as a yellow solid. $R_f$ = 0.91 (33% EtOAc in PE, with 1% $CH_3COOH$, v/v). $^1H$ NMR (400 MHz, $CDCl_3$) δ: 10.05 (brs, 1H, -COOH), 8.47 (d, *J* = 2.4 Hz, 1H, -ArH), 8.13 (d, *J* = 2.4 Hz, 1H, -ArH), 7.45 – 7.35 (m, 5H, -ArH), 3.49 (dd, *J* = 3.3 Hz, *J* = 8.8 Hz, 1H), 3.33 – 3.25 (m, 2H), 3.20 (d, *J* = 8.3 Hz, 1H), 1.00 (s, 9H, $-(CH_3)_3$). $^{13}C$ NMR (100.6 MHz, $CDCl_3$) δ: 176.3, 149.2, 145.4, 145.1, 144.2, 137.1, 129.5, 129.2, 128.6, 127.3, 119.1, 74.0, 63.7, 61.7, 27.1. HRMS (ESI-Q-TOF) m/z: $[M+Na]^+$ calcd for $C_{19}H_{21}N_3NaO_7S$ 458.0992; found, 458.1017.

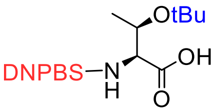


DNPBS-*L*-Thr(tBu)-OH (**12**)
89%

**Synthesis of DNPBS-*L*-Thr(tBu)-OH (12)**. **12** was synthesized on 6 mmol scale according to the general procedure. H-*L*-Thr(tBu)-OH: 4-methylmorpholine : TMSCl : DNPBS-Cl = 6 mmol : 24 mmol : 12 mmol : 6 mmol. The crude product was dissolved in 5 mL EtOAc, followed by precipitation from $Et_2O$ and PE (1/9, v/v) to give **12** (2.40 g, 89%) as a yellow solid. $R_f$ = 0.76 (33% EtOAc in PE, with 1%

$CH_3COOH$, v/v). $^{1}$H NMR (400 MHz, $CDCl_3$) δ: 10.20 (brs, 1H, -COOH), 8.65 (d, *J* = 2.4 Hz, 1H, -ArH), 8.29 (d, *J* = 2.4 Hz, 1H, -ArH), 7.59 – 7.51 (m, 5H, -ArH), 4.03 – 3.97 (m, 1H), 3.24 (d, *J* = 4.8 Hz, 1H), 3.10 (t, *J* = 4.5 Hz, 1H), 1.14 (s, 9H, $-(CH_3)_3$), 0.99 (d, *J* = 6.4 Hz, 3H). $^{13}C$ NMR (100.6 MHz, $CDCl_3$) δ: 171.6, 150.1, 145.6, 144.9, 143.8, 136.8, 129.8, 129.6, 128.2, 128.1, 118.7, 66.4, 27.8, 17.4. HRMS (ESI-Q-TOF) m/z: $[M+Na]^+$ calcd for $C_{20}H_{23}N_3NaO_7S$ 472.1149; found, 472.1165.

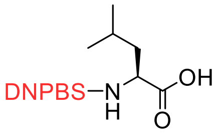


DNPBS-*L*-Leu-OH (**13**)
85%

**Synthesis of DNPBS-*L*-Leu-OH (13)**. **13** was synthesized on 6 mmol scale according to the general procedure. H-*L*-Leu-OH: 4-methylmorpholine : TMSCl : DNPBS-Cl = 7.2 mmol : 28.8 mmol : 14.4 mmol : 6 mmol. The crude product was dissolved in the minimum volume of $Et_2O$ (5 mL), followed by precipitation from PE to give **13** (2.07 g, 85%) as a yellow solid. $R_f$ = 0.83 (33% EtOAc in PE, with 1% $CH_3COOH$, v/v). $^{1}$H NMR (400 MHz, $CDCl_3$) δ: 10.72 (brs, 1H, -COOH), 8.60 (d, *J* = 2.4 Hz, 1H, -ArH), 8.29 (d, *J* = 2.4 Hz, 1H, -ArH), 7.59 – 7.51 (m, 5H, -ArH), 4.03 – 3.97 (m, 1H), 3.24 (d, *J* = 4.8 Hz, 1H), 3.10 (t, *J* = 4.5 Hz, 1H), 1.14 (s, 9H, $-(CH_3)_3$), 0.99 (d, *J* = 6.4 Hz, 3H). $^{13}C$ NMR (100.6 MHz, $CDCl_3$) δ: 179.5, 149.6, 145.5, 145.0, 144.8, 137.3, 129.5, 129.2, 128.5, 127.7, 119.0, 62.4, 41.3, 24.6, 22.5, 21.9. HRMS (ESI-Q-TOF) m/z: $[M+Na]^+$ calcd for $C_{18}H_{19}N_3NaO_6S$ 428.0887; found, 428.0937.

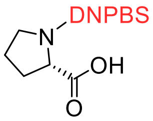


DNPBS-*L*-Pro-OH (**14**)
83%

**Synthesis of DNPBS-*L*-Pro-OH (14)**. **14** was synthesized on 6 mmol scale according to the general procedure. H-*L*-Pro-OH: 4-methylmorpholine : TMSCl : DNPBS-Cl = 7.2 mmol : 28.8 mmol : 14.4 mmol : 6 mmol. The crude product was dissolved in the minimum volume of $Et_2O$ (5 mL), followed by precipitation from PE to give **14** (1.94 g, 83%) as an orange red solid. $R_f$ = 0.83 (33% EtOAc in PE, with 1% $CH_3COOH$, v/v). $^{1}$H NMR (400 MHz, $CDCl_3$) δ: 10.12 (brs, 1H, -COOH), 8.35 (d, *J* = 2.3 Hz, 1H, -ArH), 8.06 (d, *J* = 2.3 Hz, 1H, -ArH), 7.42 – 7.30 (m, 5H, -ArH), 3.46 (dd, *J* = 3.1 Hz, *J* = 8.5 Hz, 1H), 2.87 – 2.81 (m, 1H), 1.97 – 1.88 (m, 1H), 1.80 – 1.73 (m, 1H), 1.65 – 1.60 (m, 2H). $^{13}C$ NMR (100.6 MHz, $CDCl_3$) δ: 179.0, 148.3, 145.2, 144.6, 137.5, 129.4, 129.3, 129.0, 128.6, 127.2, 118.8, 60.5, 30.1, 23.8, 14.2. HRMS (ESI-Q-TOF) m/z: $[M+H]^+$ calcd for $C_{17}H_{16}N_3O_6S$ 390.0754; found, 390.0804.

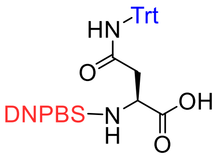


DNPBS-*L*-Asn(Trt)-OH (**15**)
76%

**Synthesis of DNPBS-*L*-Asn(Trt)-OH (15)**. **15** was synthesized on 6 mmol scale according to the general procedure. H-*L*-Asn(Trt)-OH: 4-methylmorpholine : TMSCl : DNPBS-Cl = 6 mmol : 24 mmol : 12 mmol : 6 mmol. The crude product was dissolved in the minimum volume of DCM (6 mL), followed by precipitated with PE to give **15** (2.96 g, 76%) as a yellow solid. $R_f$ = 0.83 (6% $CH_3OH$ in DCM, with 1% $CH_3COOH$, v/v). $^1H$ NMR (400 MHz, $CDCl_3$) δ: 10.46 (brs, 1H, -COOH), 8.42 (d, *J* = 2.4 Hz, 1H , -ArH), 8.19 (d, *J* = 2.4 Hz, 1H , -ArH), 7.49 – 7.41 (m, 5H, -ArH), 7.28 – 7.23 (m, 9H, -ArH), 7.13 – 7.11 (m, 6H, -ArH), 3.11 (dd, *J* = 6.7 Hz, *J* = 12.9 Hz, 1H), 2.97 (d, *J* = 7.8 Hz, 1H), 2.33 – 2.17 (m, 2H), 1.88 – 1.79 (m, 1H), 1.69 – 1.60 (m, 1H). $^{13}C$ NMR (100.6 MHz, $CDCl_3$) δ: 175.2, 172.0, 149.0, 145.0, 144.9, 144.1, 143.9, 136.7, 129.7, 129.3, 128.6, 128.0, 127.4, 127.2, 119.2, 70.9, 32.6, 28.0, 20.7. HRMS (ESI-Q-TOF) m/z: $[M+Na]^+$ calcd for $C_{35}H_{28}N_4NaO_7S$ 671.1571; found, 671.1686.

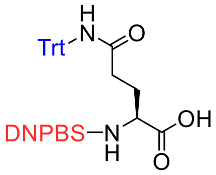


DNPBS-*L*-Gln(Trt)-OH (**16**)
96%

**Synthesis of DNPBS-*L*-Gln(Trt)-OH (16)**. **16** was synthesized on 6 mmol scale according to the general procedure. H-*L*-Gln(Trt)-OH: 4-methylmorpholine : TMSCl : DNPBS-Cl = 7.2 mmol : 28.8 mmol : 14.4 mmol : 6 mmol. The crude product was dissolved in the minimum volume of DCM (5 mL), followed by precipitation from PE to give **16** (3.82 g, 96%) as a yellow solid. $R_f$ = 0.89 (50% EtOAc in PE, with 1% $CH_3COOH$, v/v). $^1H$ NMR (400 MHz, $CDCl_3$) δ: 10.46 (brs, 1H, -COOH), 8.42 (d, *J* = 2.4 Hz, 1H, -ArH), 8.19 (d, *J* = 2.4 Hz, 1H, -ArH), 7.49 – 7.41 (m, 5H, -ArH), 7.28 – 7.23 (m, 9H, -ArH), 7.13 – 7.11 (m, 6H, -ArH), 3.11 (dd, *J* = 6.7 Hz, *J* = 12.9 Hz, 1H), 2.97 (d, *J* = 7.8 Hz, 1H), 2.33 – 2.17 (m, 2H), 1.88 – 1.79 (m, 1H), 1.69 – 1.60 (m, 1H). $^{13}C$ NMR (100.6 MHz, $CDCl_3$) δ: 175.2, 172.0, 149.0, 145.0, 144.9, 144.1, 143.9, 136.7, 129.7, 129.3, 128.6, 128.0, 127.4, 127.2, 119.2, 70.9, 32.6, 28.0, 20.7. HRMS (ESI-Q-TOF) m/z: $[M+Na]^+$ calcd for $C_{36}H_{30}N_4NaO_7S$ 685.1727; found, 685.1719.

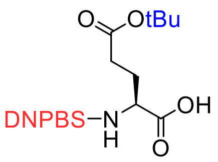


DNPBS-*L*-Glu(tBu)-OH (**17**)
94%

**Synthesis of DNPBS-*L*-Glu(tBu)-OH (17)**. **17** was synthesized on 6 mmol scale according to the general procedure. H-*L*-Glu(tBu)-OH: 4-methylmorpholine : TMSCl : DNPBS-Cl = 6 mmol : 24 mmol : 12 mmol : 6 mmol. The crude product was dissolved in the minimum volume of $Et_2O$ (6 mL), followed by precipitation from petrol ether to give **17** (2.69 g, 94%) as a yellow solid. $R_f$ = 0.83 (50% EA in PE, with 1% $CH_3COOH$, v/v). $^1H$ NMR (400 MHz, $CDCl_3$) δ: 8.63 (d, $J$ = 2.4 Hz, 1H, -ArH), 8.24 (d, $J$ = 2.4 Hz, 1H, -ArH), 7.57 – 7.42 (m, 5H, -ArH), 3.26 (dd, $J$ = 6.5 Hz, $J$ = 12.7 Hz, 1H), 2.82 (d, $J$ = 6.7 Hz, 1H), 2.19 – 2.05 (m, 2H), 1.88 – 1.79 (m, 1H), 1.72 – 1.63 (m, 1H), 1.38 (s, 9H, -$(CH_3)_3$). $^{13}C$ NMR (100.6 MHz, $CDCl_3$) δ: 177.1, 171.9, 149.6, 145.5, 144.8, 144.6, 137.3, 129.5, 129.4, 128.3, 128.0, 119.1, 81.0, 62.5, 30.8, 28.0, 27.0. HRMS (ESI-Q-TOF) m/z: $[M+Na]^+$ calcd for $C_{21}H_{23}N_3NaO_8S$ 500.1098; found, 500.1112.

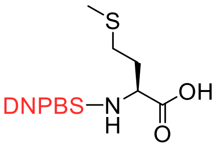


DNPBS-*L*-Met-OH (**18**)
87%

**Synthesis of DNPBS-*L*-Met-OH (18)**. **18** was synthesized on 6 mmol scale according to the general procedure. H-*L*-Met-OH: 4-methylmorpholine : TMSCl : DNPBS-Cl = 7.2 mmol : 28.8 mmol : 14.4 mmol : 6 mmol. The crude product was dissolved in 5 mL EtOAc, followed by precipitation from PE to give **18** (2.21 g, 87%) as a yellow solid. $R_f$ = 0.83 (33% EtOAc in PE, with 1% $CH_3COOH$, v/v). $^1H$ NMR (400 MHz, $CDCl_3$) δ: 9.91 (brs, 1H, -COOH), 8.55 (d, $J$ = 2.3 Hz, 1H, -ArH), 8.17 (d, $J$ = 2.3 Hz, 1H, -ArH), 7.48 – 7.33 (m, 5H, -ArH), 3.35 (dd, $J$ = 6.3 Hz, $J$ = 12.1 Hz, 1H), 2.85 (d, $J$ = 6.7 Hz, 1H), 2.32 – 2.21 (m, 2H), 1.91 (s, 3H, -$CH_3$), 1.84 – 1.74 (m, 1H), 1.68 – 1.60 (m, 1H). $^{13}C$ NMR (100.6 MHz, $CDCl_3$) δ: 178.2, 149.5, 145.4, 144.8. 144.5, 137.2, 129.6, 129.3, 128.4, 127.9, 119.2, 62.0, 30.8, 29.7, 15.2. HRMS (ESI-Q-TOF) m/z: $[M+H]^+$ calcd for $C_{17}H_{18}N_3O_6S_2$ 424.0632; found, 424.0644.

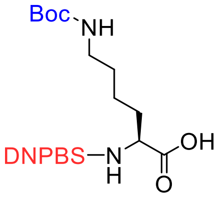


DNPBS-*L*-Lys(Boc)-OH (**19**)
94%

**Synthesis of DNPBS-*L*-Lys(Boc)-OH (19)**. **19** was synthesized on 6 mmol scale according to the general procedure. H-*L*-Lys(Boc)-OH: 4-methylmorpholine : TMSCl : DNPBS-Cl = 7.2 mmol : 28.8 mmol : 14.4 mmol : 6 mmol. The residue was dissolved in 5 mL EtOAc, followed by precipitation from petrol ether give **19** (2.94 g, 94%) as a yellow solid. $R_f$ = 0.53 (33% EtOAc in PE, with 1% $CH_3COOH$, v/v). $^1$H NMR (400 MHz, DMSO) δ: 12.51 (brs, 1H, -COOH), 8.60 (d, $J$ = 2.4 Hz, 1H, -ArH), 8.14 (d, $J$ = 2.4 Hz, 1H, -ArH), 7.56 – 7.50 (m, 3H, -ArH), 7.49 – 7.44 (m, 2H, -ArH), 6.65 (m, 1H), 4.46 (d, $J$ = 7.6 Hz, 1H), 3.02 (dd, $J$ = 7.1 Hz, $J$ = 13.1 Hz, 1H), 2.77 (dd, $J$ = 6.7 Hz, $J$ = 12.9 Hz, 2H), 1.46 – 1.40 (m, 1H), 1.36 (s, 9H, -OtBu), 1.33 – 1.29 (m, 1H), 1.23 – 1.20 (m, 2H), 1.12 – 1.04 (m, 2H). $^{13}$C NMR (100.6 MHz, $CDCl_3$) δ: 174.7, 156.0, 149.2, 145.2, 145.0, 143.3, 137.2, 129.7, 129.3, 127.1, 119.9, 77.8, 63.6, 31.4, 29.7, 28.7, 22.8. HRMS (ESI-Q-TOF) m/z: $[M+Na]^+$ calcd for $C_{23}H_{28}N_4NaO_8S$ 543.1520; found, 543.1524.

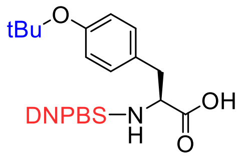


DNPBS-*L*-Tyr(tBu)-OH (**20**)
83%

**Synthesis of DNPBS-*L*-Tyr(tBu)-OH (20)**. **20** was synthesized on 6 mmol scale according to the general procedure. H-*L*-Tyr(tBu)-OH: 4-methylmorpholine : TMSCl : DNPBS-Cl = 6 mmol : 24 mmol : 12 mmol : 6 mmol. The crude product was dissolved in 5 mL EtOAc, followed by precipitation from $Et_2O$ and PE (1/9, v/v) to give **20** (2.55 g, 83%) as a yellow solid. $R_f$ = 0.78 (6% $CH_3OH$ in DCM, with 1% $CH_3COOH$, v/v). $^1$H NMR (400 MHz, $CDCl_3$) δ: 8.55 (d, $J$ = 2.4 Hz, 1H, -ArH), 8.18 (d, $J$ = 2.4 Hz, 1H, -ArH), 7.48 – 7.39 (m, 5H, -ArH), 6.85 – 6.80 (m, 4H, -ArH), 3.55 (dd, $J$ = 6.3 Hz, $J$ = 13.0 Hz, 1H), 2.90 – 2.85 (m, 1H), 2.78 – 2.73 (m, 2H), 1.31 (s, 9H, $-(CH_3)_3$). $^{13}$C NMR (100.6 MHz, $CDCl_3$) δ: 177.1, 154.5, 149.3, 145.3, 144.8, 137.2, 129.8, 129.6, 129.4, 129.2, 128.5, 127.6, 124.2, 119.1, 78.6, 64.4, 37.2, 28.8. HRMS (ESI-Q-TOF) m/z: $[M+Na]^+$ calcd for $C_{25}H_{25}N_3NaO_7S$ 534.1305; found,534.1296.

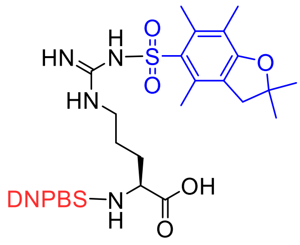


DNPBS-*L*-Arg(Pbf)-OH (**21**)
83%

**Synthesis of DNPBS-*L*-Arg(Pbf)-OH (21)**. **21** was synthesized on 6 mmol scale according to the general procedure. H-*L*-Arg(Pbf)-OH: 4-methylmorpholine : TMSCl : DNPBS-Cl = 6 mmol : 48 mmol :24 mmol : 6 mmol. The crude product was

subjected to purification on a flash silica gel column chromatography (33% EA in PE, with 1% $CH_3COOH$, v/v) to afford yellow solid, which was precipitated against $Et_2O$ and petrol ether (1/9, v/v) to give **21** (3.79 g, 83%) as a yellow solid. $R_f = 0.51$ (33% EA in PE, with 1% $CH_3COOH$, v/v). $^1H$ NMR (400 MHz, $CDCl_3$) δ: 8.57 (d, $J = 2.3$ Hz, 1H, -ArH), 8.11 (d, $J = 2.3$ Hz, 1H, -ArH), 7.48 – 7.43 (m, 5H, -ArH), 6.81 (br, 2H), 4.01 (br, 1H), 2.95 (s, 2H), 2.86 (m, 3H), 2.47 (s, 3H), 2.42 (s, 3H), 1.99 (s, 3H), 1.90 (brs, 1H), 1.40 (s, 6H), 1.23 – 1.10 (m, 4H). $^{13}C$ NMR (100.6 MHz, $CDCl_3$) δ: 157.9, 156.8, 149.2, 146.0, 144.9, 143.3, 137.7, 137.2, 134.9, 131.9, 129.6, 129.2, 127.0, 124.7, 119.8, 116.7, 86.7, 43.0, 28.8, 26.8, 25.8, 19.4, 18.0, 12.7. HRMS (ESI-Q-TOF) m/z: $[M+H]^+$ calcd for $C_{31}H_{37}N_6O_9S_2$ 701.2058; found, 701.2172.

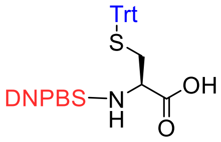


DNPBS-*L*-Cys(Trt)-OH (**22**)
87%

**Synthesis of DNPBS-*L*-Cys(Trt)-OH (22)**. **22** was synthesized on 6 mmol scale according to the general procedure. H-*L*-Cys(Trt)-OH: 4-methylmorpholine : TMSCl : DNPBS-Cl = 6 mmol : 24 mmol : 12 mmol : 6 mmol. The crude product was dissolved in the minimum volume of DCM (5 mL), followed by precipitation from petrol ether to give **22** (3.33 g, 87%) as a yellow solid. $R_f = 0.51$ (33% EA in PE, with 1% $CH_3COOH$, v/v). $^1H$ NMR (400 MHz, $CDCl_3$) δ: 10.37 (brs, 1H, -COOH), 8.64 (d, $J = 2.1$ Hz, 1H, -ArH), 8.20 (d, $J = 2.2$ Hz, 1H, -ArH), 7.47 – 7.44 (m, 5H, -ArH), 7.29 – 7.22 (m, 15H, -ArH), 2.91 (dd, $J = 5.7$ Hz, $J = 11.4$ Hz, 1H), 2.80 (d, $J = 5.6$ Hz, 1H), 2.43 (dd, $J = 5.2$ Hz, $J = 12.9$ Hz, 1H), 2.32 (dd, $J = 6.6$ Hz, $J = 12.8$ Hz, 1H). $^{13}C$ NMR (100.6 MHz, $CDCl_3$) δ: 176.5, 149.5, 145.8, 145.3, 144.7, 144.0, 137.5, 129.4, 129.3, 128.3, 128.0, 126.9, 118.9, 66.9, 33.5, 22.7. HRMS (ESI-Q-TOF) m/z: $[M+Na]^+$ calcd for $C_{34}H_{27}N_3NaO_6S_2$ 660.1233; found, 660.1248.

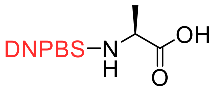


DNPBS-*L*-Ala-OH (**23**)
88%

**Synthesis of DNPBS-*L*-Ala-OH (23)**. **23** was synthesized on 6 mmol scale according to the general procedure. H-*L*-Ala-OH: 4-methylmorpholine : TMSCl : DNPBS-Cl = 7.2 mmol : 28.8 mmol : 14.4 mmol : 6 mmol. The residue was dissolved in the minimum volume of $Et_2O$, followed by precipitation from petrol ether to give **23** (1.92 g, 88%) as a yellow solid. $R_f = 0.83$ (33% EA in PE, with 1% $CH_3COOH$, v/v). $^1H$ NMR (400 MHz, $CDCl_3$) δ: 10.44 (brs, 1H, -COOH), 8.63 (d, $J = 2.4$ Hz, 1H, -ArH), 8.25 (d, $J = 2.4$ Hz, 1H, -ArH), 7.55 – 7.48 (m, 5H, -ArH), 3.29 (m, 1H), 2.75 (m, 1H), 1.14 (d, $J = 7.1$ Hz, 3H). $^{13}C$ NMR (100.6 MHz, $CDCl_3$) δ: 179.0, 149.4, 145.3, 145.2, 144.2, 137.2, 129.6, 129.4, 128.3, 127.9, 119.1, 58.5, 17.7. HRMS (ESI-Q-TOF) m/z: $[M+Na]^+$ calcd for $C_{15}H_{13}N_3NaO_6S$, 386.0423; found, 386.0401.

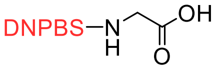


DNPBS-Gly-OH (**24**)
90%

**Synthesis of DNPBS-Gly-OH (24)**. **24** was synthesized on 6 mmol scale according to the general procedure. H- Gly-OH: 4-methylmorpholine : TMSCl : DNPBS-Cl = 7.2 mmol : 28.8 mmol : 14.4 mmol : 6 mmol. The residue was dissolved in 5 mL EtOAc, followed by precipitation from petrol ether to give **24** (1.89 g, 90%) as a yellow solid. $R_f$ = 0.21 (4.8% $CH_3OH$ in DCM, with 1% $CH_3COOH$, v/v). $^1H$ NMR (400 MHz, $CDCl_3$) δ: 9.82 (brs, 1H, -COOH), 8.67 (d, $J$ = 2.4 Hz, 1H, -ArH), 8.24 (d, $J$ = 2.4 Hz, 1H, -ArH), 7.52 (m, 5H, -ArH), 3.31(s, 2H), 2.57 (brs, 1H, -NH). $^{13}C$ NMR (100.6 MHz, $CDCl_3$) δ: 176.0, 149.2, 145.4, 145.3, 143.9, 137.3, 129.7, 129.5, 128.4, 127.9, 119.3, 52.4. HRMS (ESI-Q-TOF) m/z: $[M-H]^-$ calcd. for C14H10N3O6S, 348.0296; found, 348.0306.

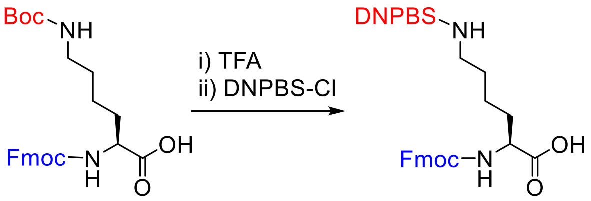


Fmoc-*L*-Lys(Boc)-OH    Fmoc-*L*-Lys(DNPBS)-OH (**47**)

**Synthesis of Fmoc-*L*-Lys(DNPBS)-OH (47)**. To a solution of Fmoc-*L*-Lys(Boc)-OH (3 g, 6.4 mmol) in 50 mL DCM was added 50 mL TFA. After stirring at room temperature for 2 h under an argon atmosphere, the reaction mixture was concentrated on vacuum to 1/10 of the original volume. 100 mL $Et_2O$ was added to the residue and precipitant was collected by filtration and washed with $Et_2O$, then dried on vacuum to give Fmoc-*L*-Lys($NH_2$·TFA)-OH (2.97 g, 99%), which used in next step without purification.

To a solution of Fmoc- *L*-Lys($NH_2$·TFA)-OH (2.97 g, 6.38 mmol) in 50 mL anhydrous DCM was added pyridine (10.3 mL). When the mixture was completely clarified, DNPBS-Cl (1.98 g, 6.38 mmol) in anhydrous DCM (20 mL) was added dropwise slowly to the solution. After stirring at room temperature for 20 min under an argon atmosphere, the reaction mixture was washed with 2% aqueous solution of citric acid (100 mL) twice, then the organic layer was dried over sodium sulfate, filtered and concentrated on vacuum. The residue was purified on flash aluminum oxide (50% EtOAc in PE, with 1% $CH_3COOH$, v/v) to give **46** (2.5 g, 61%) as a yellow solid. $R_f$ = 0.81 (50% EtOAc in PE, with 1% $CH_3COOH$, v/v). $^1H$ NMR (400 MHz, DMSO) δ: 12.55 (brs, 1H, -COOH), 8.66 (d, $J$ = 2.4 Hz, 1H, -ArH), 8.16 (d, $J$ = 2.4 Hz, 1H, -ArH), 7.89 – 7.87 (m, 2H, -ArH), 7.73 – 7.70 (m, 2H, -ArH), 7.57 – 7.50 (m, 6H, -ArH, -NH), 7.43 – 7.39 (m, 2H, -ArH), 7.33 – 7.30 (m, 2H, -ArH), 4.32 – 4.27 (m, 2H), 4.25 – 4.20 (m, 1H), 3.86 – 3.80 (m, 1H), 3.44 (t, $J$ = 5.4 Hz, 1H), 2.43 – 2.41 (m, 2H), 1.60 – 1.45 (m, 2H), 1.16 – 1.11 (m, 4H). $^{13}C$ NMR (100.6 MHz, DMSO) δ:

174.4, 156.6, 149.2, 145.2, 145.1, 144.3, 144.2, 143.0, 141.2, 137.2, 129.8, 129.5, 128.9, 128.1, 127.7, 125.7, 120.5, 119.9, 65.4, 54.1, 51.7, 47.1, 29.0, 26.8, 23.1. HRMS (ESI-Q-TOF) m/z: $[M+H]^+$ calcd for $C_{33}H_{31}N_4O_8S$ 643.1857; found, 643.1844.

**Stability of DNPBS protected amino acid.**

DNPBS-*L*-Ile-OH (**9**) was dissolved in DMF-*d7* or THF-*d8* (final 10 mM). The solutions were kept at room temperature and $^1$H NMR was measured at intervals.

**UV-Vis spectrometry of 3 and 4.**

UV-Vis spectrometry of **3** and **4** was measured using a JASCO J-715 spectropolarimeter instrument. The samples were prepared by dissolving **3** or **4** in pyridine or THF (final concentration 33 mg/ L). Spectra were recorded at 25°C using a 1 cm path length cuvette. Each spectrum was an average of 3 scans and the absorb spectra were recorded from 600 to 200 nm.

**Preparation of racemic model dipeptide.**

To a magnetically stirred suspension of 1:1 mixture of Fmoc-*L*-AA-OH (AA means amino acid) and Fmoc-*D*-AA-OH (0.115 mmol) in 2 mL of anhydrous DMF was successively added *L*-Leu-OtBu (26 µL, 0.126 mmol), Oxyma (18 mg, 0.126 mmol) and DIC (13 mL, 0.126 mmol). After stirring 30 min at room temperature, the reaction mixture was purified by preparative TLC to give the racemate of Fmoc-*L*-AA-*L*-Leu-OtBu and Fmoc-*D*-AA-*L*-Leu-OtBu as a white solid. The ee value was determined by chiral chromatography analysis.

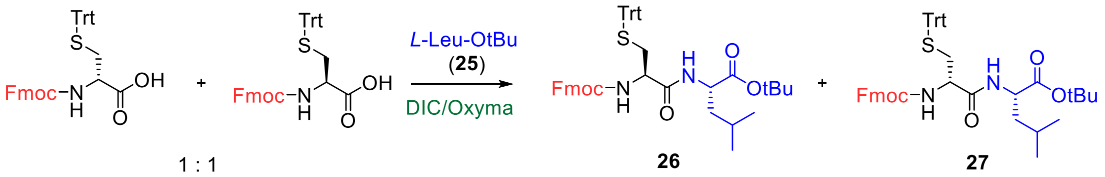


Racemate of Fmoc-*L*-Cys(Trt)-*L*-Leu-OtBu (**26**) and Fmoc-*D*-Cys(Trt)-*L*-Leu-OtBu (**27**), white solid, $R_f$ = 0.56 (20% EtOAc in PE, v/v). Chiral chromatography analysis conditions: Chiralpak IC column (hexanes: isopropanol = 80:20, flow rate = 1.0 mL/min, column temperature = 30 ℃, wavelength = 254 nm), $t_R$ = 5.50 min (Fmoc-*D*-Cys(Trt)-*L*-Leu-OtBu), $t_R$ = 7.06 min (Fmoc-*L*-Cys(Trt)-*L*-Leu-OtBu).

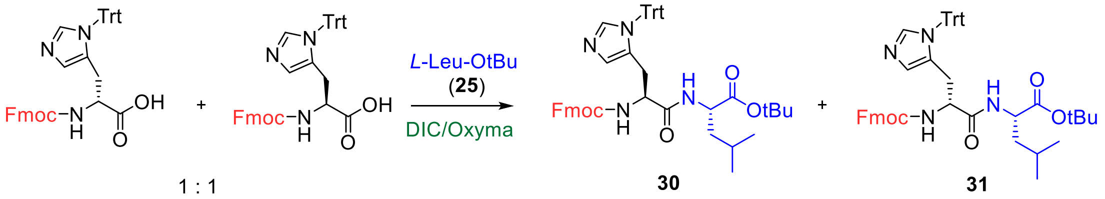


Racemate of Fmoc-*L*-His(Trt)-*L*-Leu-OtBu (**30**) and Fmoc-*D*-His(Trt)-*L*-Leu-OtBu (**31**), white solid, $R_f$ = 0.53 (33% EtOAc in petrol ehter, with 1% $Et_3N$). Chiral chromatography analysis conditions: Chiralpak AD-H column (hexanes: isopropanol

= 80:20, flow rate = 1.0 mL/min, column temperature = 30 ℃, wavelength = 254 nm), tR = 5.66 min (Fmoc-*L*-His(Trt)-*L*-Leu-OtBu), tR = 15.45 min (Fmoc-*D*-His(Trt)-*L*-Leu-OtBu).

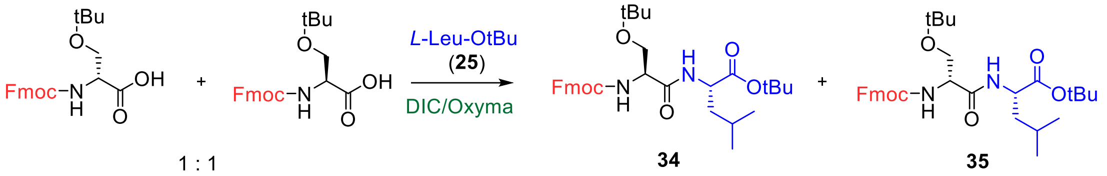


Racemate of Fmoc-*L*-Ser(tBu)-*L*-Leu-OtBu (**34**) and Fmoc-*D*-Ser(tBu)-*L*-Leu-OtBu (**35**), white solid, $R_f$ = 0.58 (20% EtOAc in PE, v/v). Chiral chromatography analysis conditions: Chiralpak IC column (hexanes: isopropanol = 80:20, flow rate = 1.0 mL/min, column temperature = 30 ℃, wavelength = 254 nm), tR = 6.93 min (Fmoc-*D*-Ser(tBu)-*L*-Leu-OtBu), tR = 9.19 min (Fmoc-*L*-Ser(tBu)-*L*-Leu-OtBu).

To a magnetically stirred suspension of racemate of DNPBS-*L*-AA-OH and DNPBS-*D*-AA-OH (0.115 mmol) in 2 mL of anhydrous THF was successively added *L*-Leu-OtBu (26 µL, 0.126 mmol), Oxyma (18 mg, 0.126 mmol) and DIC (13 mL, 0.126 mmol). After stirring 30 min at room temperature, the reaction mixture was purified by preparative TLC to give the racemate of DNPBS-*L*-AA-*L*-Leu-OtBu and DNPBS-*D*-AA-*L*-Leu-OtBu as a yellow solid. The ee value was determined by chiral chromatography analysis.

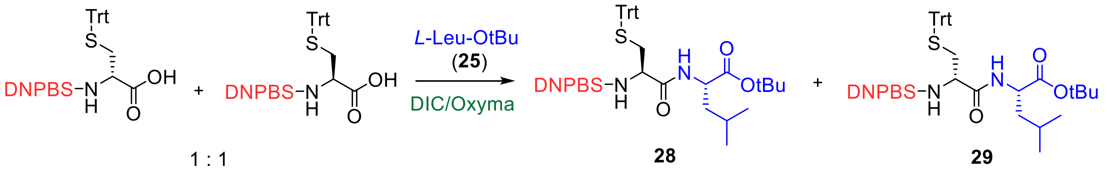


Racemate of DNPBS-*L*-Cys(Trt)-*L*-Leu-OtBu (**28**) and DNPBS-*D*-Cys(Trt)-*L*-Leu-OtBu (**29**), white solid, $R_f$ = 0.63 (20% EtOAc in PE, v/v). Chiral chromatography analysis conditions: Chiralpak Cellulose-1 column (hexanes: isopropanol = 80:20, flow rate = 1.0 mL/min, column temperature = 30 ℃, wavelength = 254 nm), tR = 6.31 min (DNPBS-*L*-Cys(Trt)-*L*-Leu-OtBu), tR = 8.00 min (DNPBS-*D*-Cys(Trt)-*L*-Leu-OtBu).

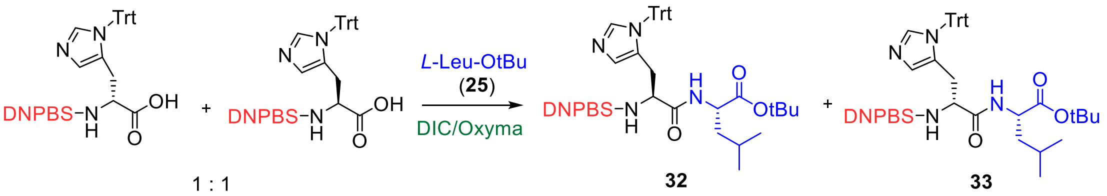


Racemate of DNPBS-*L*-His(Trt)-*L*-Leu-OtBu (**32**) and DNPBS-*D*-His(Trt)-OH-*L*-Leu-OtBu (**33**), yellow solid, $R_f$ = 0.63 (33% EtOAc in PE, with 1% $Et_3N$). Chiral chromatography analysis conditions: Chiralpak AD-H column

(hexanes: isopropanol = 80:20, flow rate = 1.0 mL/min, column temperature = 30 ℃, wavelength = 254 nm), tR = 8.38 min (DNPBS-*L*-His(Trt)-*L*-Leu-OtBu), tR = 13.62 min (DNPBS-*D*-His(Trt)-*L*-Leu-OtBu).

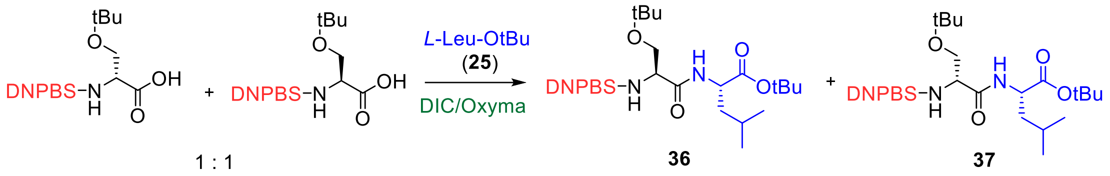


Racemate of DNPBS-*L*-Ser(tBu)-*L*-Leu-OtBu (**36**) and DNPBS-*D*-Ser(tBu)-*L*-Leu-OtBu (**37**), yellow solid, $R_f$ = 0.65 (20% EtOAc in PE, v/v). Chiral chromatography analysis conditions: Chiralpak Cellulose-1 column (hexanes: isopropanol = 80:20, flow rate = 1.0 mL/min, column temperature = 30 ℃, wavelength = 254 nm), tR = 6.68 min (DNPBS-*D*-Ser(tBu)-*L*-Leu-OtBu), tR = 7.24 min (DNPBS-*L*- Ser(tBu)-*L*-Leu-OtBu).

**Effect of different coupling reagents on the α-C racemization**

**PyBop/DIPEA**: DNPBS-*L*-AA-OH (0.115 mmol) or Fmoc-*L*-AA-OH (0.115 mmol) in 2 mL of anhydrous DMF was added PyBop (0.126 mmol) and DIPEA (0.23 mmol). After stirring 5 min at room temperature, *L*-Leu-OtBu (0.126 mmol) was added to the solution, which resumed stirring at room temperature for 30 min. The reaction mixture was purified by preparative TLC to give Fmoc-AA-*L*-Leu-OtBu or DNPBS-AA-*L*-Leu-OtBu. The ee value was determined by chiral chromatography analysis.

**HBTU/DIPEA**: DNPBS-*L*-AA-OH (0.115 mmol) or Fmoc-*L*-AA-OH (0.115 mmol) in 2 mL of anhydrous DMF was added HBTU (0.126 mmol) and DIPEA (0.23 mmol). After stirring 5 min at room temperature, *L*-Leu-OtBu (0.126 mmol) was added to the solution, which resumed stirring at room temperature for 30 min. The reaction mixture was purified by preparative TLC to give Fmoc-AA-*L*-Leu-OtBu or DNPBS-AA-*L*-Leu-OtBu. The ee value was determined by chiral chromatography analysis.

**HATU/NMM**: DNPBS-*L*-AA-OH (0.115 mmol) or Fmoc-*L*-AA-OH (0.115 mmol) in 2 mL of anhydrous DMF was added HATU (0.126 mmol) and 4-methylmorpholine (0.23 mmol). After stirring 5 min at room temperature, *L*-Leu-OtBu (0.126 mmol) was added to the solution, which resumed stirring at room temperature for 30 min. The reaction mixture was purified by preparative TLC to give Fmoc-AA-*L*-Leu-OtBu or DNPBS-AA-*L*-Leu-OtBu. The ee value was determined by chiral chromatography analysis.

**EDCI/HOBt**: DNPBS-*L*-AA-OH (0.115 mmol) or Fmoc-*L*-AA-OH (0.115 mmol) in 2 mL of anhydrous DMF was added EDCI (0.126 mmol) and HOBt (0.023 mmol). After stirring 5 min at room temperature, *L*-Leu-OtBu (0.126 mmol) was added to the solution, which resumed stirring at room temperature for 30 min. The reaction mixture was purified by preparative TLC to give Fmoc-AA-*L*-Leu-OtBu or DNPBS-AA-*L*-Leu-OtBu. The ee value was determined by chiral chromatography

analysis.

**DIC/Oxyma**: DNPBS-*L*-AA-OH or Fmoc-*L*-AA-OH (0.115 mmol) in 2 mL of anhydrous THF or Fmoc-*L*-AA-OH (0.115 mmol) in 2 mL of anhydrous DMF was added Oxyma (0.126 mmol) and DIC (0.126 mmol). After stirring 5 min at room temperature, *L*-Leu-OtBu (0.126 mmol) was added to the solution, which resumed stirring at room temperature for 30 min. The reaction mixture was purified by preparative TLC to give Fmoc- AA-*L*-Leu-OtBu or DNPBS- AA-*L*-Leu-OtBu. The ee value was determined by chiral chromatography analysis. In addition, the same coupling reaction was conducted at 55 ℃.

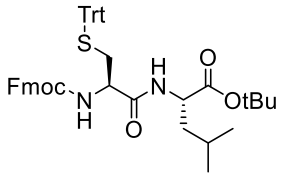


Fmoc-*L*-Cys(Trt)-*L*-Leu-OtBu (**26**)

White solid, $R_f$ = 0.53 (20% EtOAc in PE, v/v). $^1$H NMR (400 MHz, $CDCl_3$) δ: 7.64 – 7.60 (m, 2H, -ArH), 7.46 – 7.44 (m, 2H, -ArH), 7.33 – 7.23 (m, 8H, -ArH), 7.17 – 7.14 (m, 8H, -ArH), 7.10 – 7.07 (m, 3H, -ArH), 6.18 (d, *J* = 8.0 Hz, 1H), 5.04 (d, *J* = 8.0 Hz, 1H), 4.29 – 4.23 (m, 2H), 4.08 (t, *J* = 6.9 Hz, 1H), 3.73 (m, 1H), 2.64 (dd, *J* = 8.0 Hz, *J* = 13.0 Hz, 1H), 2.50 (dd, *J* = 4.6 Hz, *J* = 12.8 Hz, 1H), 1.49 – 1.44 (m, 2H), 1.30 (s, 9H, -OtBu), 1.17 (m, 1H), 0.78 (d, *J* = 5.7 Hz, 6H). $^{13}$C NMR (100.6 MHz, $CDCl_3$) δ: 171.4, 169.7, 156.0, 144.5, 143.9, 143.7, 141.3. 129.7, 128.1, 127.8, 127.2, 127.0, 125.2, 120.0, 81.9, 67.4, 54.0, 51.7, 47.1, 41.8, 34.0, 29.8, 28.0, 24.9, 22.8, 22.2. HRMS (ESI-Q-TOF) m/z: $[M+Na]^+$ calcd for $C_{47}H_{50}N_2NaO_5S$ 777.3333; found, 777.3311.

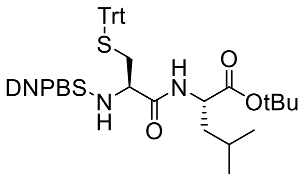


DNPBS-*L*-Cys(Trt)-*L*-Leu-OtBu (**28**)

Yellow solid, $R_f$ = 0.63 (20% EtOAc in PE, v/v). $^1$H NMR (400 MHz, $CDCl_3$) δ: 8.48 (d, *J* = 2.4 Hz, 1H, -ArH), 8.11 (d, *J* = 2.4 Hz, 1H, -ArH), 7.39 – 7.31 (m, 5H, -ArH), 7.21 – 7.09 (m, 15H, -ArH), 5.61 (d, *J* = 8.5 Hz, 1H), 4.17 – 4.12 (m, 1H), 2.73 (d, *J* = 5.7 Hz, 1H), 2.32 (dd, *J* = 6.0 Hz, *J* = 12.6 Hz, 1H), 2.26 – 2.16 (m, 2H), 1.42 – 1.36 (m, 2H), 1.27 (s, 9H, -OtBu), 1.18 (m, 1H), 0.81 (d, *J* = 6.2 Hz, 3H), 0.75 (d, *J* = 6.1 Hz, 3H). $^{13}$C NMR (100.6 MHz, $CDCl_3$) δ: 171.2, 169.5, 149.4, 145.6, 145.4, 144.7, 144.2, 137.6, 129.5, 129.4, 129.2, 128.4, 128.0, 126.9, 118.9, 81.8, 67.0, 64.8, 51.3, 41.6, 34.9, 27.9, 24.8, 22.7, 22.0. HRMS (ESI-Q-TOF) m/z: $[M+Na]^+$ calcd for $C_{44}H_{46}N_4NaO_7S_2$ 829.2700; found, 829.2735.

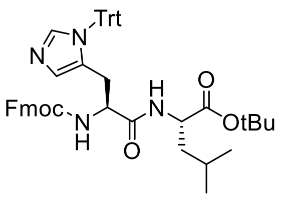

Fmoc-*L*-His(Trt)-*L*-Leu-OtBu (**30**)

White solid, R$_f$ = 0.52 (33% EtOAc in PE, with 1% $Et_3N$). $^1$H NMR (400 MHz, $CDCl_3$) δ: 7.75 (d, *J* = 7.5 Hz, 2H, -ArH), 7.66 – 7.60 (m, 3H, -ArH), 7.40 – 7.39 (m, 2H, -ArH), 7.31 – 7.26 (m, 11H, -ArH), 7.12 – 7.10 (m, 6H, -ArH), 4.56 (dd, *J* = 6.0 Hz, *J* = 12.5 Hz, 1H), 4.45 (dd, *J* = 8.1 Hz, *J* = 13.8 Hz, 1H), 4.34 (d, *J* = 7.4 Hz, 2H), 4.21 (t, *J* = 7.0 Hz, 1H), 3.08 (d, *J* = 5.5 Hz, 2H), 1.67 – 1.55 (m, 2H), 1.42 (s, 9H, -OtBu), 1.31 – 1.25 (m, 1H). $^{13}$C NMR (100.6 MHz, $CDCl_3$) δ: 171.7, 171.0, 144.0, 142.3, 141.3, 138.3, 136.9, 129.8, 128.1, 127.7, 127.1, 125.3, 125.2, 119.9, 119.5, 81.5, 75.4, 67.2, 51.8, 47.2, 41.6, 28.0, 24.9, 22.822.2. HRMS (ESI-Q-TOF) m/z: $[M+H]^+$ calcd for $C_{50}H_{53}N_4O_5$ 789.4010; found, 789.4030.

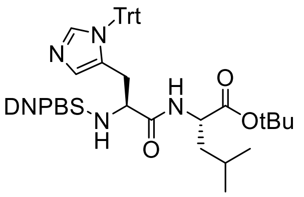

DNPBS-*L*-His(Trt)-*L*-Leu-OtBu (**32**)

Yellow solid, R$_f$ = 0.57 (33% EtOAc in PE, with 1% $Et_3N$). $^1$H NMR (400 MHz, $CDCl_3$) δ: 8.44 (d, *J* = 1.9 Hz, 1H, -ArH), 8.10 (d, *J* = 1.9 Hz, 1H, -ArH), 7.33 (m, 5H, -ArH), 7.23 – 7.18 (m, 9H, -ArH), 7.01 – 6.95 (m, 7H, -ArH), 6.43 (s, 1H), 4.22 – 4.16 (m, 1H), 3.90 (d, *J* = 4.8 Hz, 1H), 3.38 (dd, *J* = 5.9 Hz, *J* = 11.5 Hz, 1H), 2.66 (dd, *J* = 6.1 Hz, *J* = 14.9 Hz, 1H), 2.55 (dd, *J* = 6.2 Hz, *J* = 14.9 Hz, 1H), 1.41 – 1.37 (m, 4H), 1.25 (s, 9H, -OtBu), 0.78 (d, *J* = 6.1 Hz, 3H), 0.73 (d, *J* = 6.0 Hz, 3H). $^{13}$C NMR (100.6 MHz, $CDCl_3$) δ: 171.5, 171.3, 149.2, 145.4, 145.1, 144.3, 142.3, 138.5, 137.3, 136.6, 129.7, 129.3, 129.1, 128.5, 128.1, 127.5, 119.3, 119.2, 81.4, 75.3, 65.3, 53.4, 51.3, 44.2, 41.4, 31.2, 28.1, 27.9, 24.9, 22.9, 22.8, 22.0, 21.9. HRMS (ESI-Q-TOF) m/z: $[M+H]^+$ calcd for $C_{47}H_{49}N_6O_7S$ 841.3378; found, 841.3490.

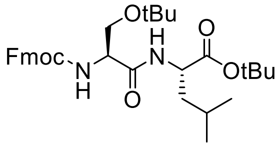

Fmoc-*L*-Ser(tBu)-*L*-Leu-OtBu (**34**)

White solid, R$_f$ = 0.51 (20% EtOAc in PE, v/v). $^1$H NMR (400 MHz, $CDCl_3$) δ: 7.67 – 7.65 (m, 2H, -ArH), 7.53 – 7.51 (m, 2H, -ArH), 7.32 – 7.28 (m, 2H, -ArH), 7.24 – 7.15 (m, 3H, -ArH), 5.71 (m, 1 H), 4.41 (m, 1H), 4.35 – 4.27 (m, 2 H) , 4.14 (m, 2 H) , 3.75 (q, *J* = 3.7 Hz, 1 H), 3.32 (t, *J* = 8.1 Hz, 1 H), 1.66 – 1.52 (m, 2 H), 1.47 - 1.42 (m, 1 H), 1.37 (s, 9H, -OtBu), 1.14 (s, 9H, -OtBu), 0.86 (d, *J* = 6.3 Hz, 6 H). $^{13}$C NMR (100.6 MHz, $CDCl_3$) δ: 171.6, 169.9, 156.1, 143.9, 143.8, 141.3, 127.7, 127.1, 125.2,

120.0, 81.7, 74.3, 67.1, 61.8, 54.3, 51.7, 47.2, 41.9, 28.0, 27.4, 24.9, 22.8, 22.2. HRMS (ESI-Q-TOF) m/z: $[M+H]^+$ calcd for $C_{32}H_{44}N_2NaO_6$ 575.3092; found, 575.3231.

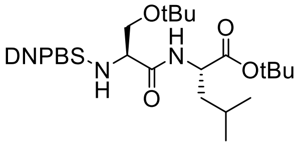


DNPBS-*L*-Ser(tBu)-*L*-Leu-OtBu (**36**)

Yellow solid, $R_f$ = 0.61 (20% EtOAc in PE, v/v). $^1$H NMR (400 MHz, $CDCl_3$) δ: 8.54 (m, 1H, -ArH), 8.18 (m, 1H, -ArH), 7.43 (m, 5H, -ArH), 6.64 (d, *J* = 8.0 Hz, 1H), 4.27 (m, 1H), 3.34 (m, 1H), 3.16 – 3.08 (m, 3H), 1.47 – 1.44 (m, 2H), 1.33 (s, 9H, -OtBu), 1.18 (m, 1H), 1.00 (s, 9H, -OtBu), 0.84 – 0.80 (m, 6H). $^{13}$C NMR (100.6 MHz, $CDCl_3$) δ:171.5, 169.7, 149.4, 145.4, 145.1, 144.5, 137.4, 129.4, 129.3, 128.4, 128.0, 119.1, 81.6, 73.8, 64.6, 61.4, 51.2, 41.8, 29.7, 27.9, 27.2, 24.7, 22.8, 22.0. HRMS (ESI-Q-TOF) m/z: $[M+Na]^+$ calcd for $C_{29}H_{40}N_4O_8S$ 627.2459; found, 627.2602.

**Fmoc SPPS**

Fmoc SPPS-based peptide synthesis was carried out parallelly with DNPBS-based SPPS. The resin, coupling times and reaction conditions were exactly the same as that employed in DNPBS SPPS except following specifications:

Coupling: DMF was used as the coupling solvent.

Fmoc deprotection: Fmoc group was removed by incubation with 2 mL of deprotection solution (20% piperidine in DMF) at room temperature (total 2 × 10 min).

**Analysis of aspartimide formation in different SPPS strategies**

*DNPBS SPPS strategy*: Tripeptide FDG was synthesized on Rink amide-AM resin (loading 0.338 mmol/g) at 0.05 mmol scale based on DNPBS SPPS. The final DNPBS deprotection step was carried out by incubation with 2 mL of deprotection solution (1 M *p*-toluenethiol in pyridine) at room temperature twice (10 min each time) or at 50°C for 60 min. Then resin was treated with 95% TFA (with 2.5% each of water and triisopropylsilane) at room temperature for 1 h and the obtained solution was analyzed directly by UPLC-MS. UPLC conditions: C18 column (ACQUITY UPLC® BEH C18, 100 × 2.1 mm, 1.7 μm); column temperature 35°C; A linear gradient from 2 to 20 vol% acetonitrile in water containing 0.1 vol% TFA over 10 min; flow rate, 0.4 mL/min; The detection wavelength was 210 nm. $t_R$ (desired peptide) = 3.0 – 3.5 min, m/z, $[MH]^+$ cal. 337.1506, found 337.1618; $t_R$ (aspartmide) = 4.2 – 5.8 min, m/z, $[MH]^+$ cal. 319.1401 , found 319.1424; $t_R$ (α + β piperidide) = 8.2 and 9.9 min, m/z $[MH]^+$ cal. 404.2292, found 404.2375.

*Fmoc SPPS strategy*: Tripeptide FDG was synthesized on Rink aminomethyl polystyrene resin (loading 0.338 mmol/g) at 0.05 mmol scale based Fmoc SPPS. The final Fmoc deprotection step was carried out by incubation with 2 mL of 20%

piperidine in DMF at room temperature twice (10 min each time) or at 50°C for 60 min. The post-synthetic treatment was the same as the above DNPBS SPPS strategy.

**Analysis of α-C racemization in different SPPS strategies**

Peptide CGHSF was synthesized on Rink aminomethyl polystyrene resin (loading 0.338 mmol/g) at 0.025 mmol scale using either DNPBS SPPS or Fmoc SPPS. After post-synthetic treatment, the full-length peptide was isolated by HPLC and was subjected to aminopeptidase M (APM) digestion: To a solution of peptide (5.6 μg/μL) in Tris buffer (25 mM, pH 7.5, 500 mM NaCl) was added 0.175 U of APM, total reaction volume 100 μL. The mixture was incubated at 37 ℃ for 24 h, followed by quenching with TFA (final 1 vol%). The obtained sample was subjected to UPLC-MS analysis directly. UPLC conditions: C18 column (ACQUITY UPLC® BEH C18, 100 × 2.1 mm, 1.7 μm); column temperature: 35°C; A linear gradient from 2 to 20 vol% acetonitrile in water containing 0.1 vol% TFA over 10 min; flow rate, 0.4 mL/min; The detection wavelength was 210 nm.

# Supplementary Figures

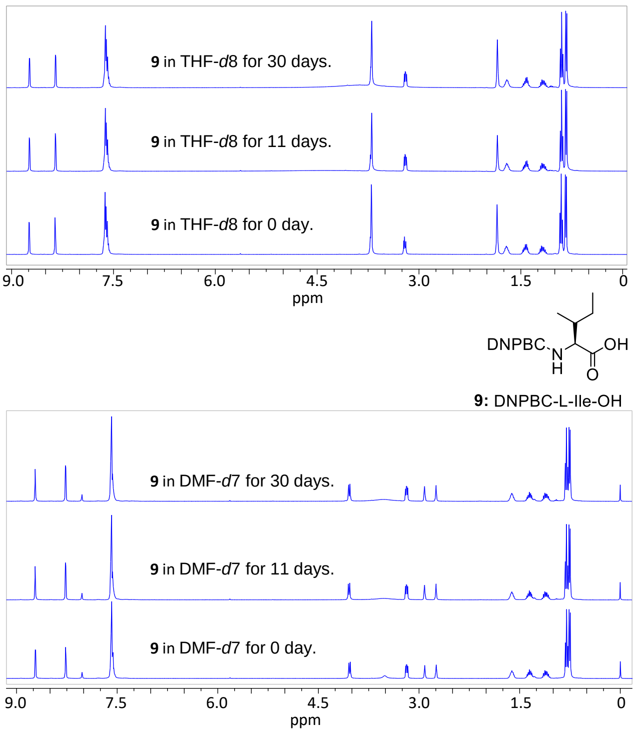


**Supplementary Figure 1**. $^{1}$H NMR spectrum of **9** in DMF-*d7* or THF-*d8* for 30 days.

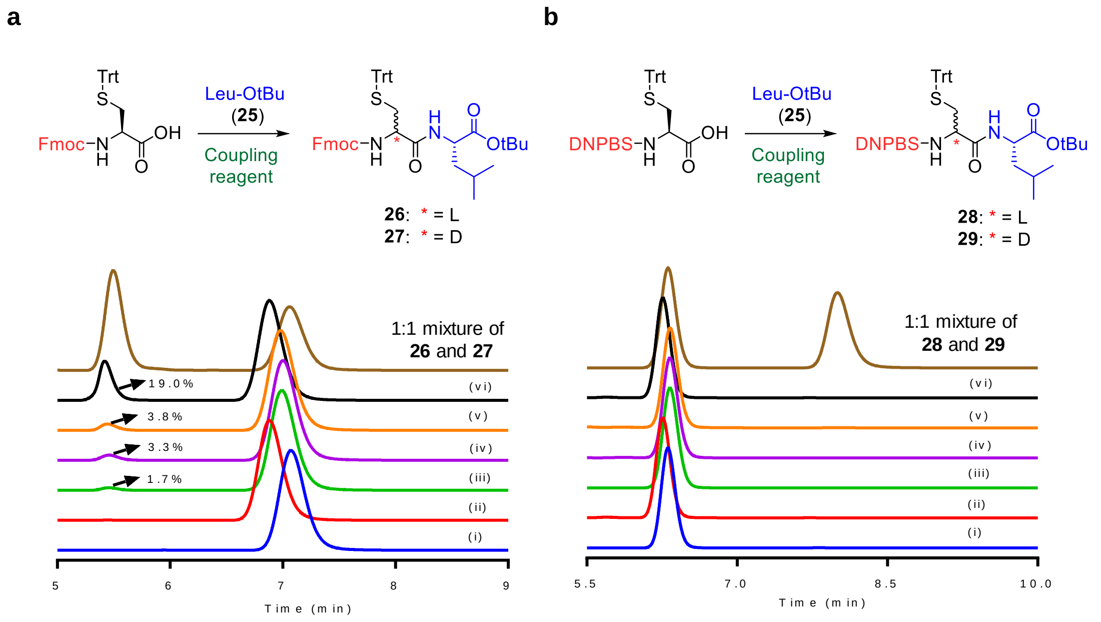


**Supplementary Figure 2**. UPLC analysis of α-C racemization for coupling of Cys under different coupling reagents. (a) Fmoc strategy. (b) DNPBS strategy. Coupling reagents: (i) DIC/Oxyma, R.T.; (ii) DIC/Oxyma, 55 ℃; (iii) EDCI/HOBt, R.T.; (iv) PyBop/DIPEA, R.T.; (v) HBTU/DIPEA, R.T.; (vi) HATU/NMM, R.T.

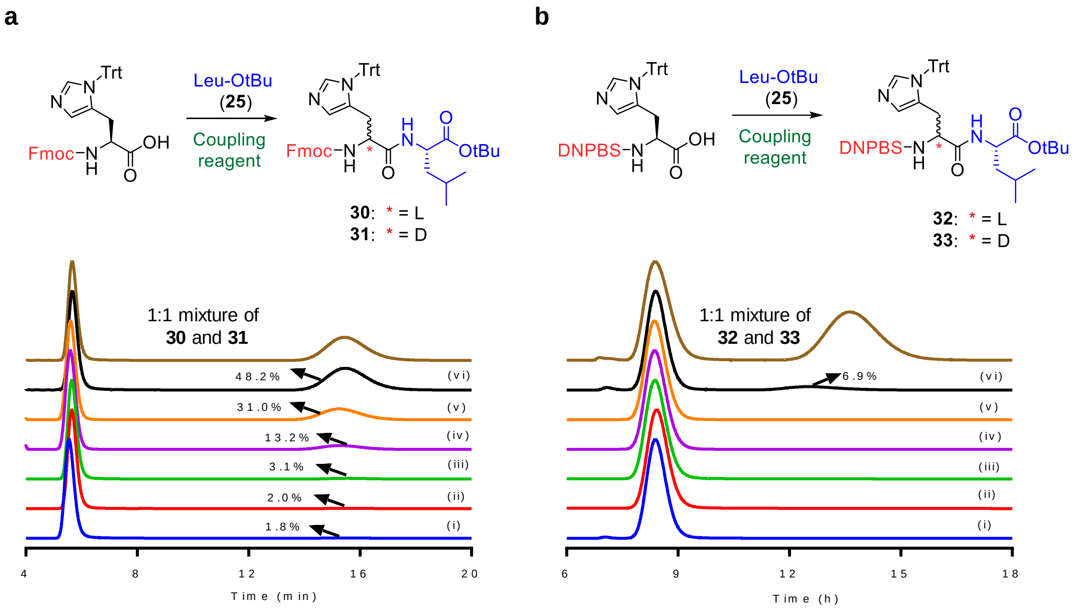


**Supplementary Figure 3**. UPLC analysis of α-C racemization for coupling of His under different coupling reagents. (a) Fmoc strategy. (b) DNPBS strategy. Coupling reagents: (i) DIC/Oxyma, R.T.; (ii) PyBop/DIPEA, R.T.; (iii) HBTU/DIPEA, R.T.; (iv) HATU/NMM, R.T. (v) DIC/Oxyma, 55 ℃; (vi) EDCI/HOBt, R.T.

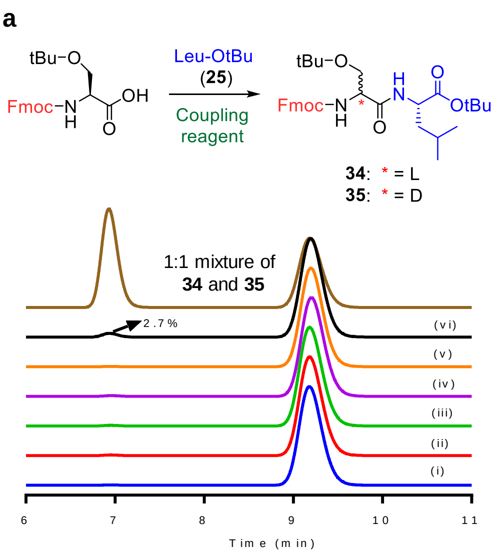


**Supplementary Figure 4**. UPLC analysis of α-C racemization for coupling of Ser under different coupling reagents. (a) Fmoc strategy. (b) DNPBS strategy. Coupling reagents: (i) DIC/Oxyma, R.T.; (ii) PyBop/DIPEA, R.T.; (iii) HBTU/DIPEA, R.T.; (iv) EDCI/HOBt, R.T.; (v) DIC/Oxyma, 55 ℃; (vi) HATU/NMM, R.T.

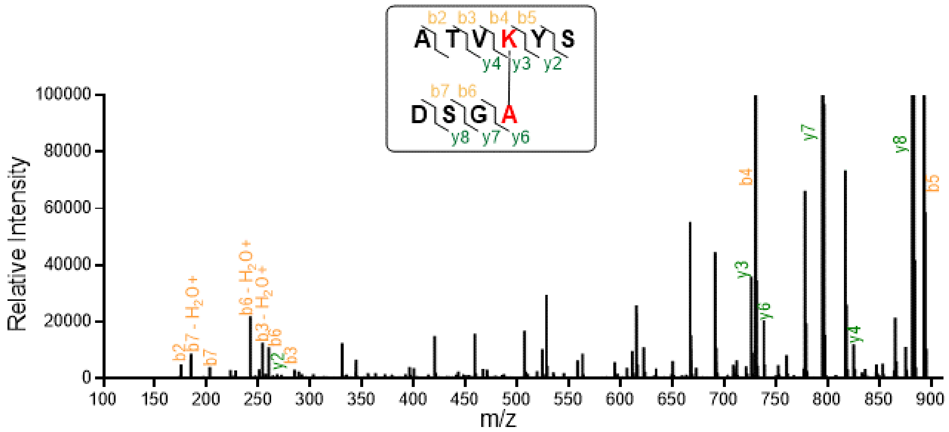


**Supplementary Figure 5**. MS-MS of branched peptide **50**.

## NMR spectra of synthesized new compounds

$^{1}H$ NMR spectrum of **1** (in $CDCl_3$):

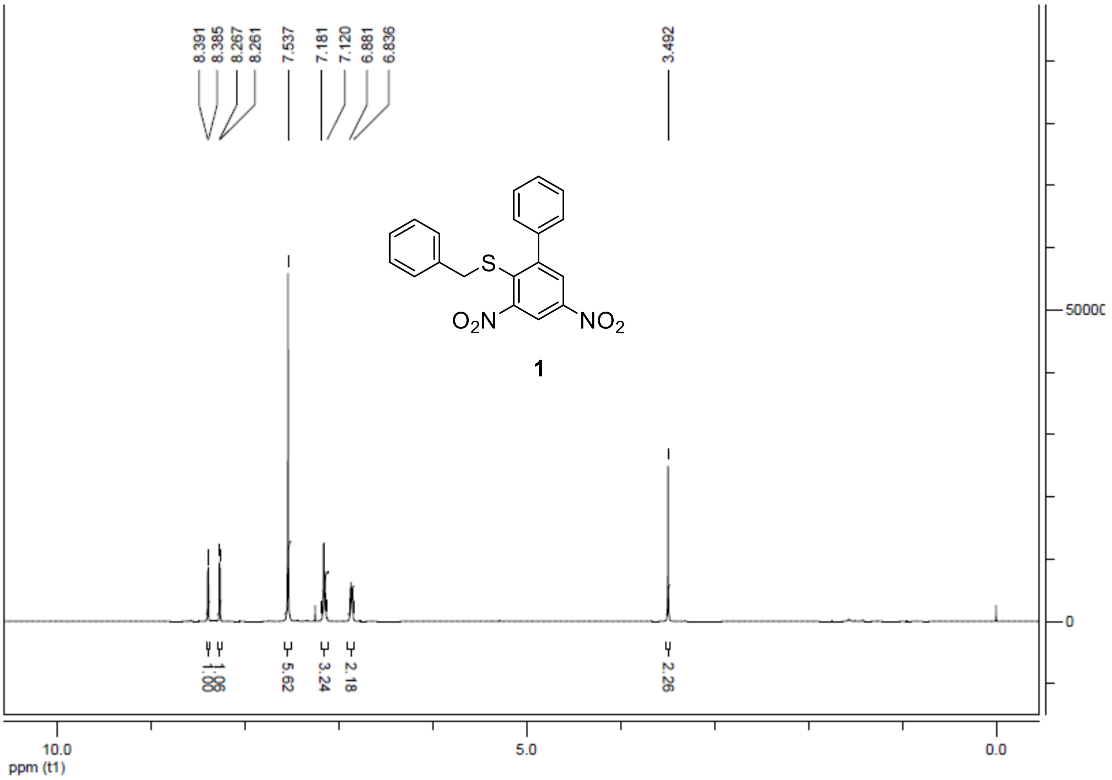


$^{13}C$ NMR spectrum of **1** (in $CDCl_3$):

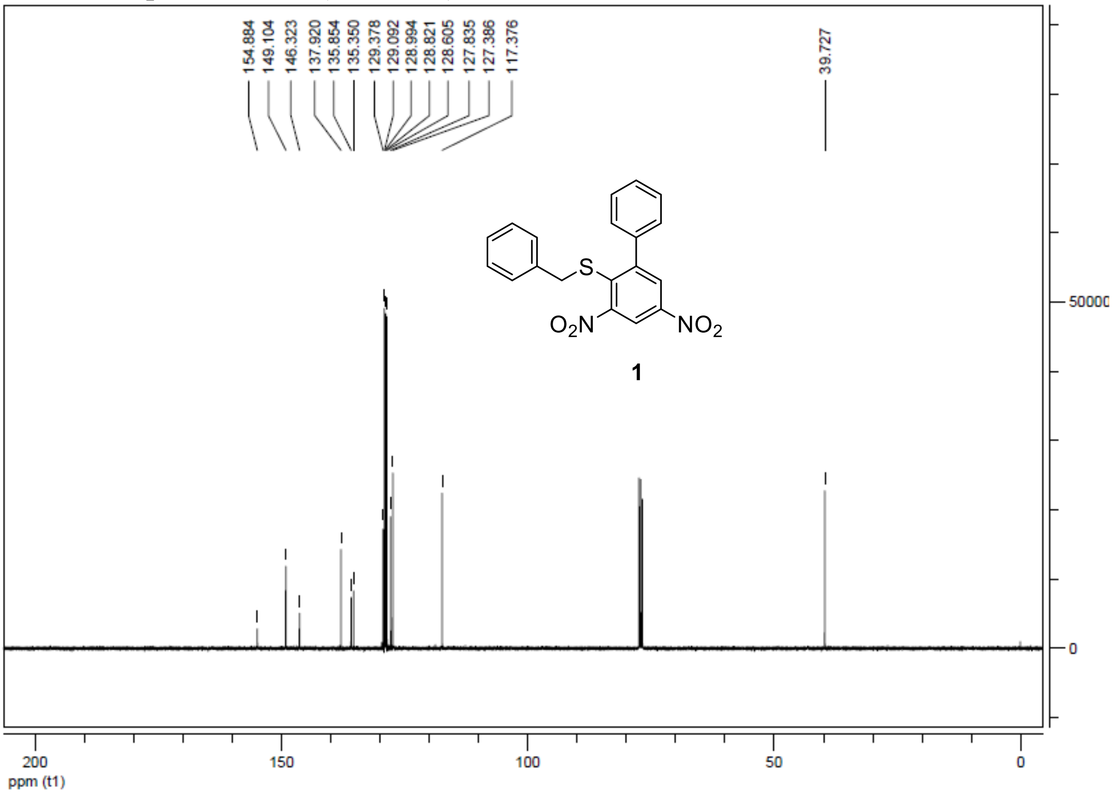

$^{1}$H NMR spectrum of **2** (in $CDCl_3$):

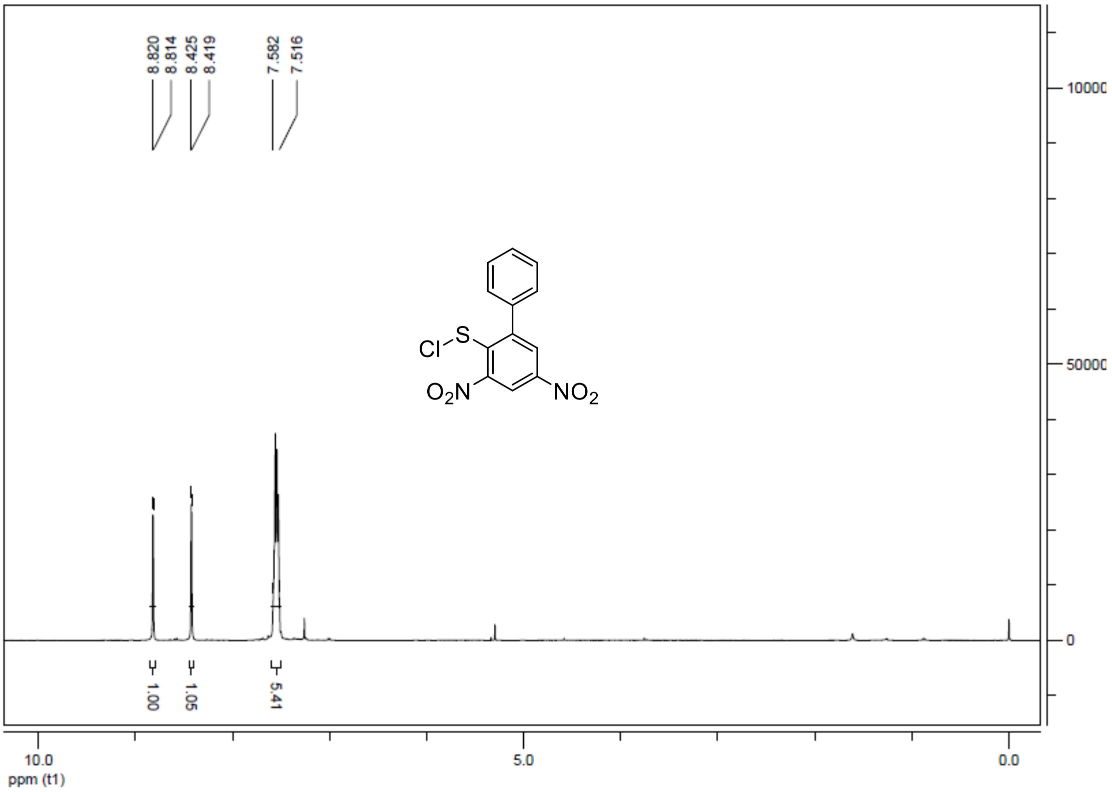


$^{13}$C NMR spectrum of **2** (in $CDCl_3$):

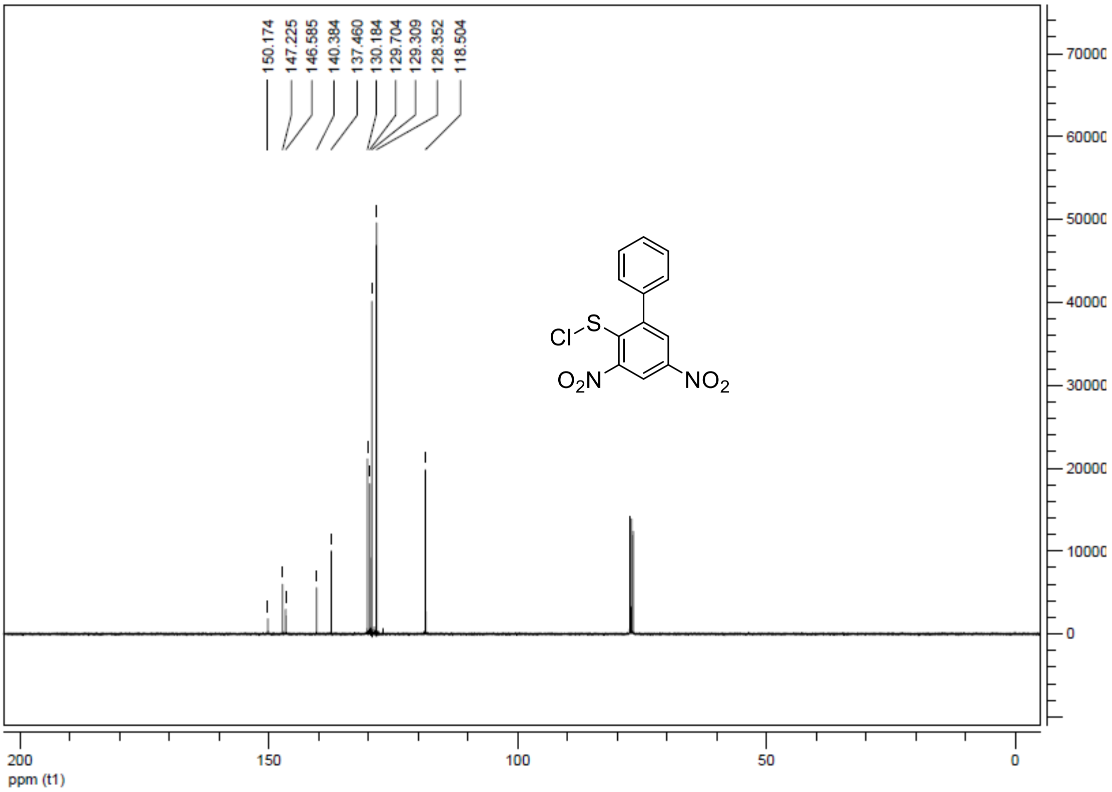

$^{1}$H NMR spectrum of **3** (in $CDCl_3$):

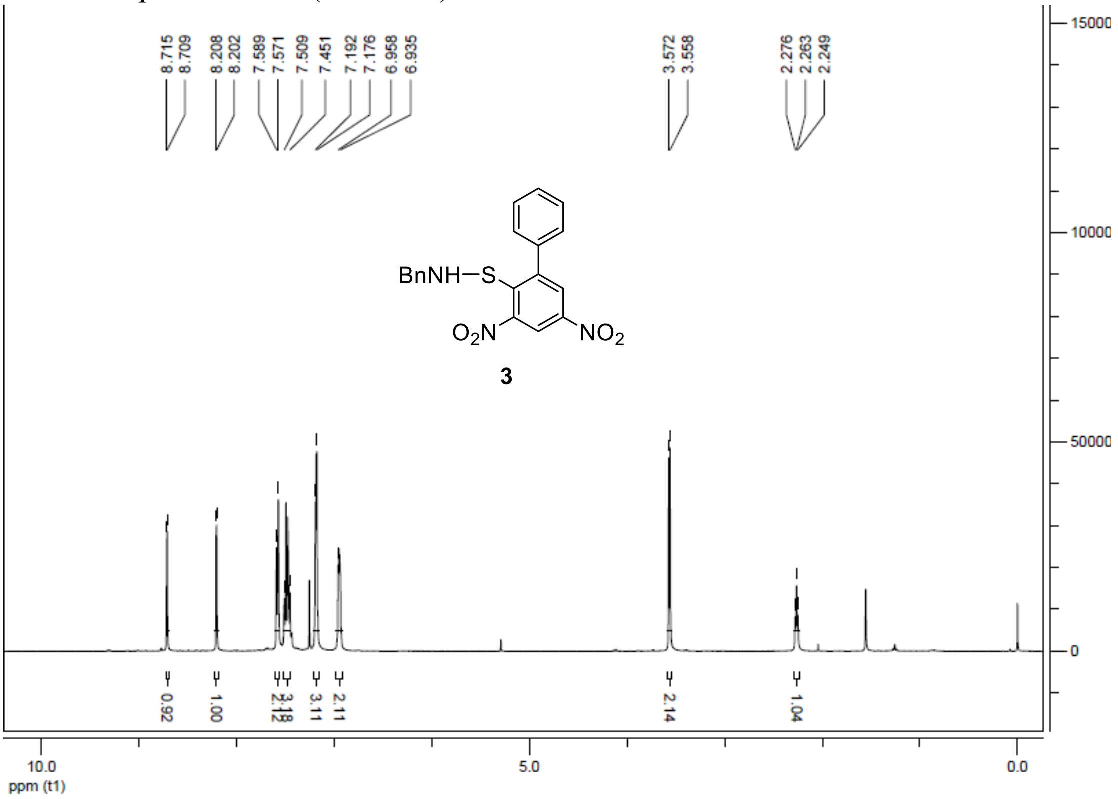


$^{13}$C NMR spectrum of **3** (in $CDCl_3$):

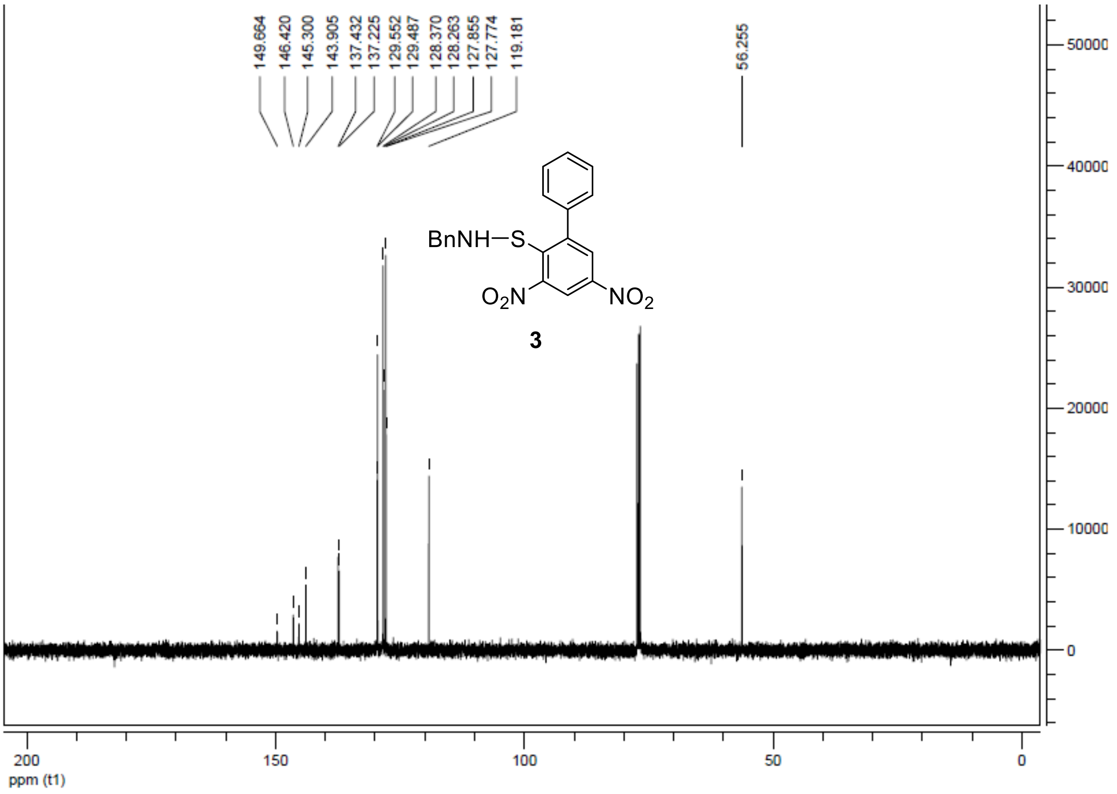

$^1H$ NMR spectrum of **4** (in $CDCl_3$):

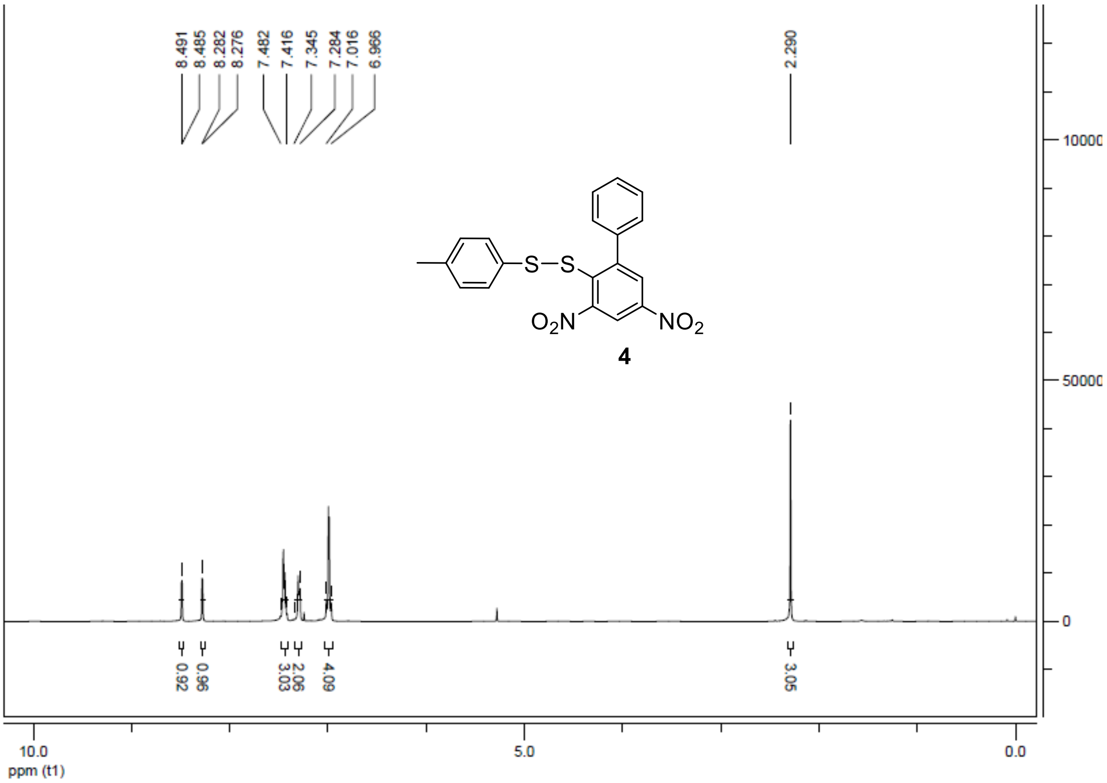


$^{13}C$ NMR spectrum of **4** (in $CDCl_3$):

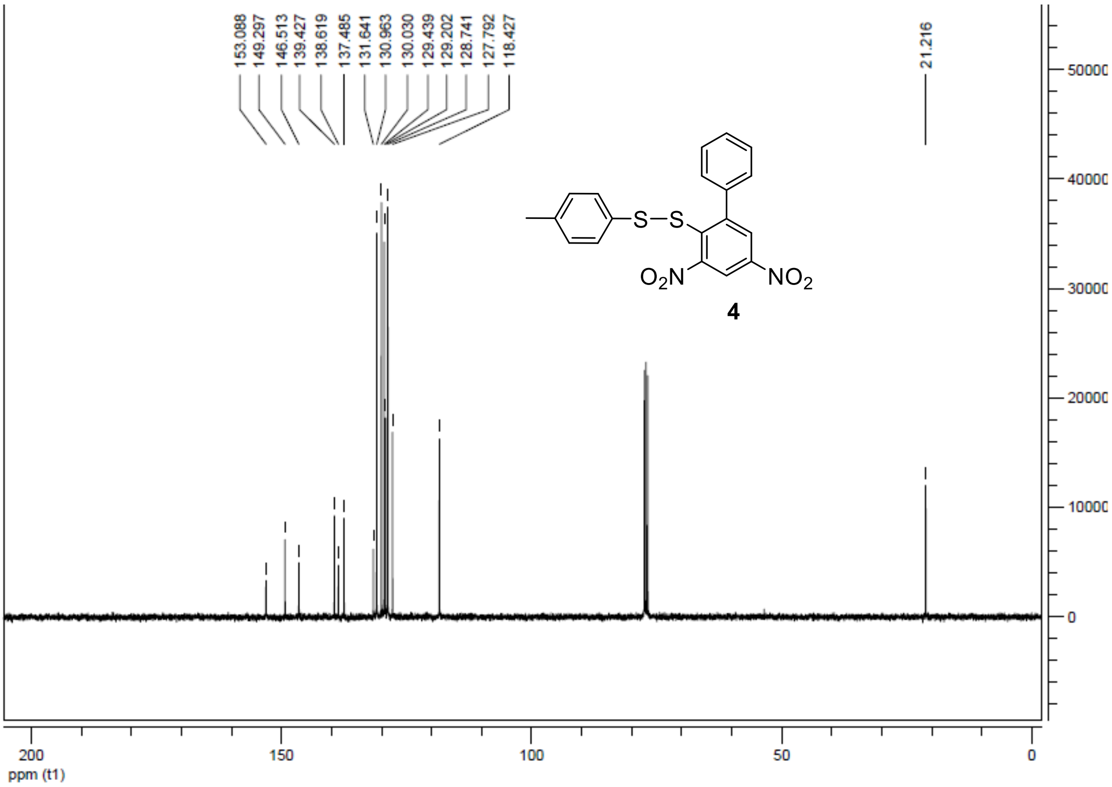

$^{1}$H NMR spectrum of **5** (in DMSO):

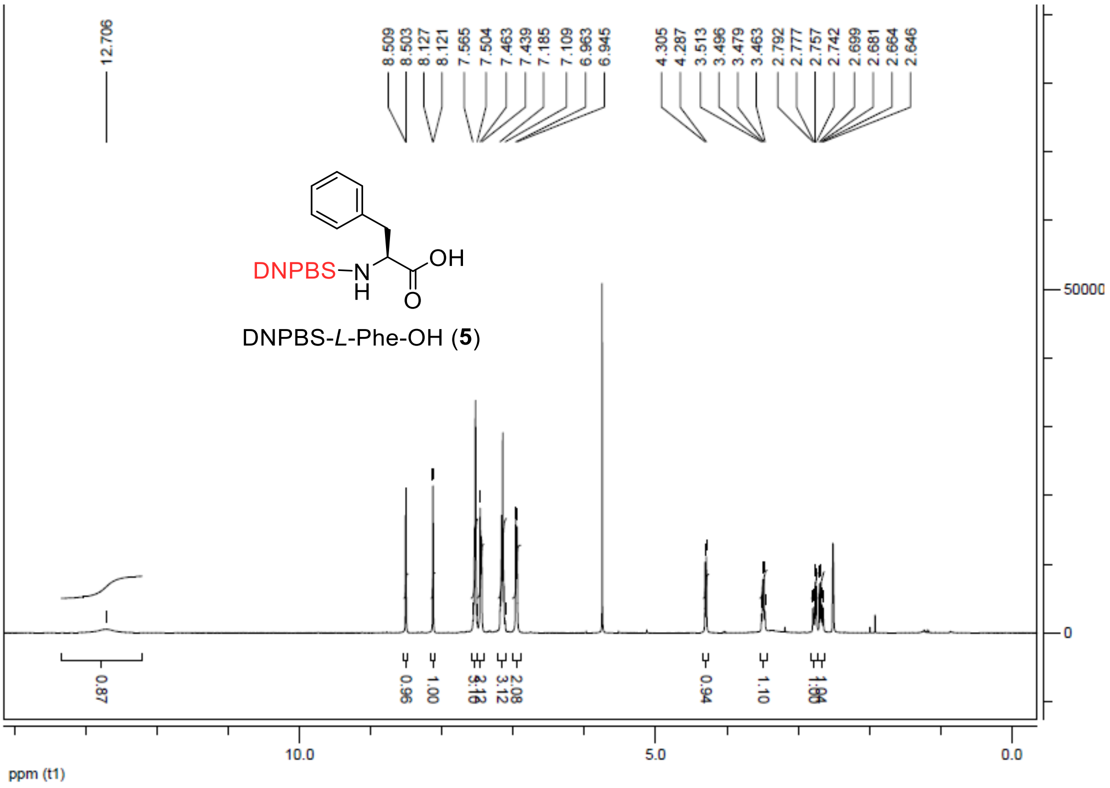


$^{13}$C NMR spectrum of **5** (in DMSO):

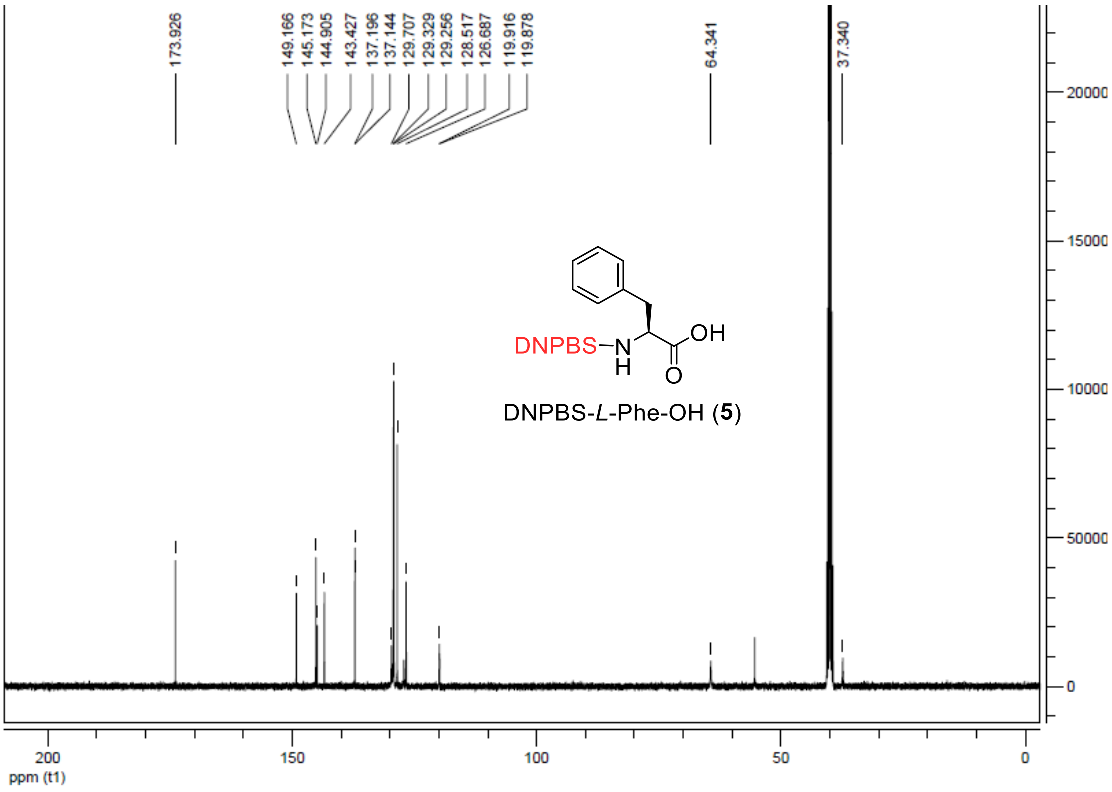

$^{1}$H NMR spectrum of **6** (in $CDCl_3$):

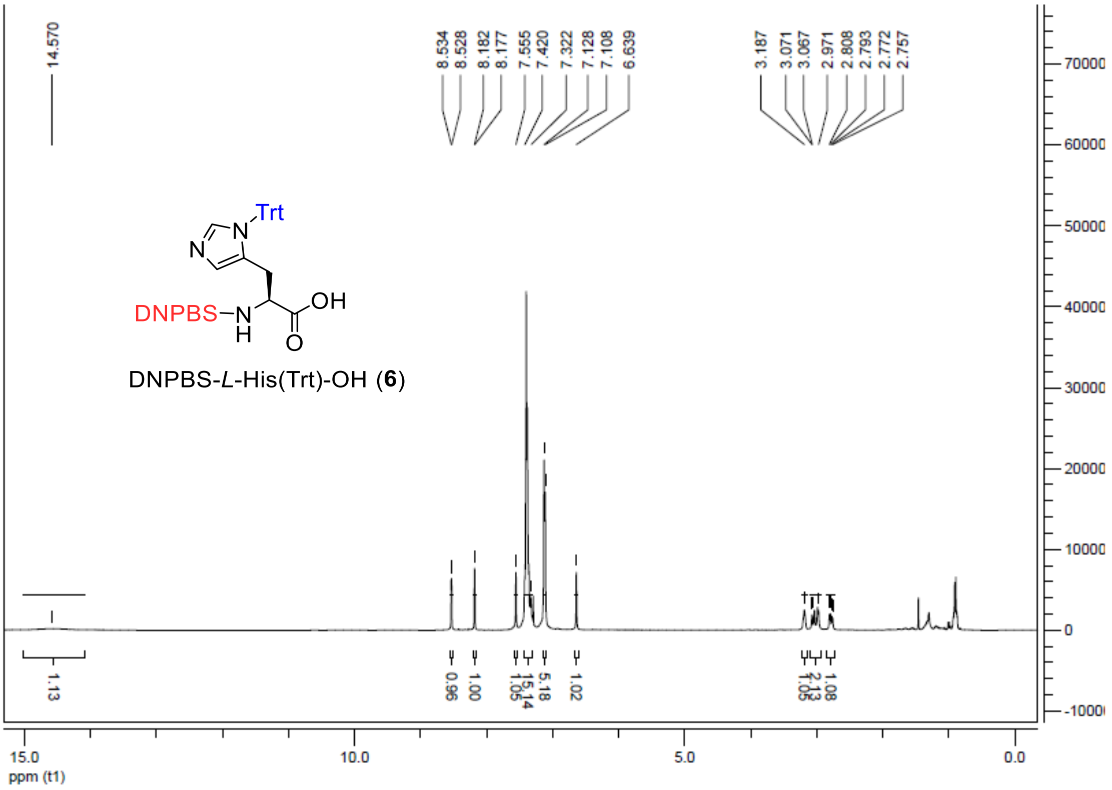


$^{13}$C NMR spectrum of **6** (in $CDCl_3$).

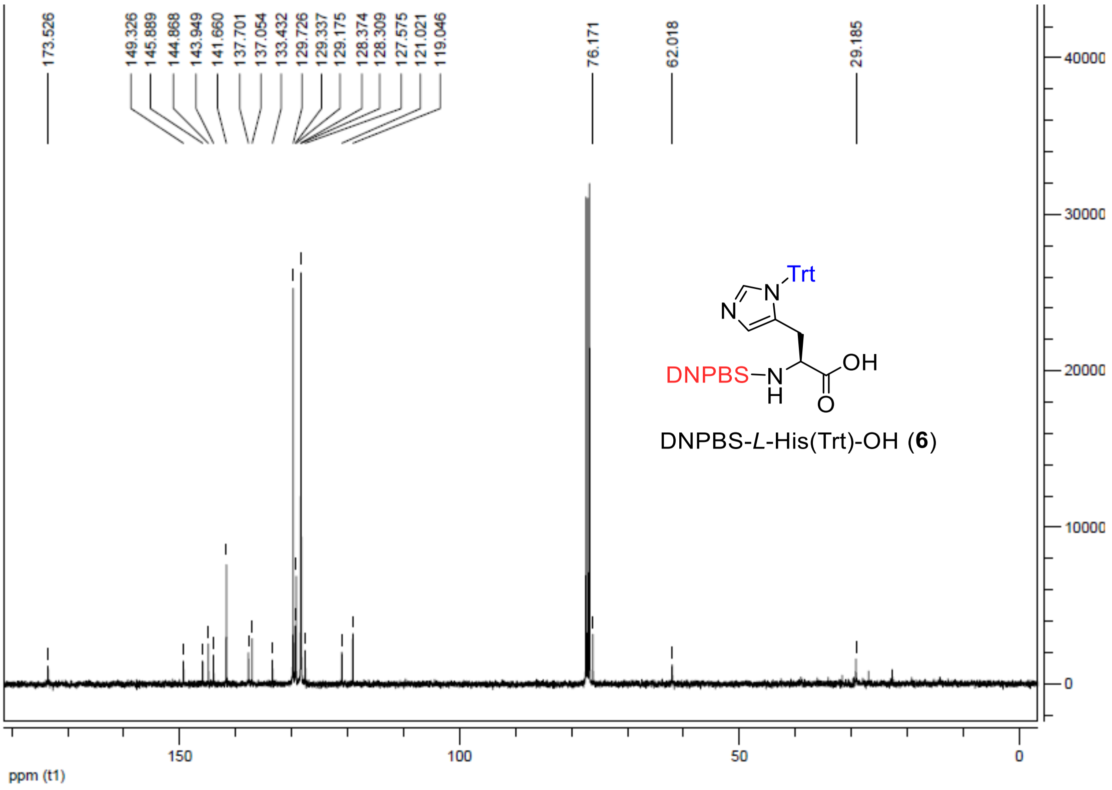

$^{1}$H NMR spectrum of **7** (in DMSO):

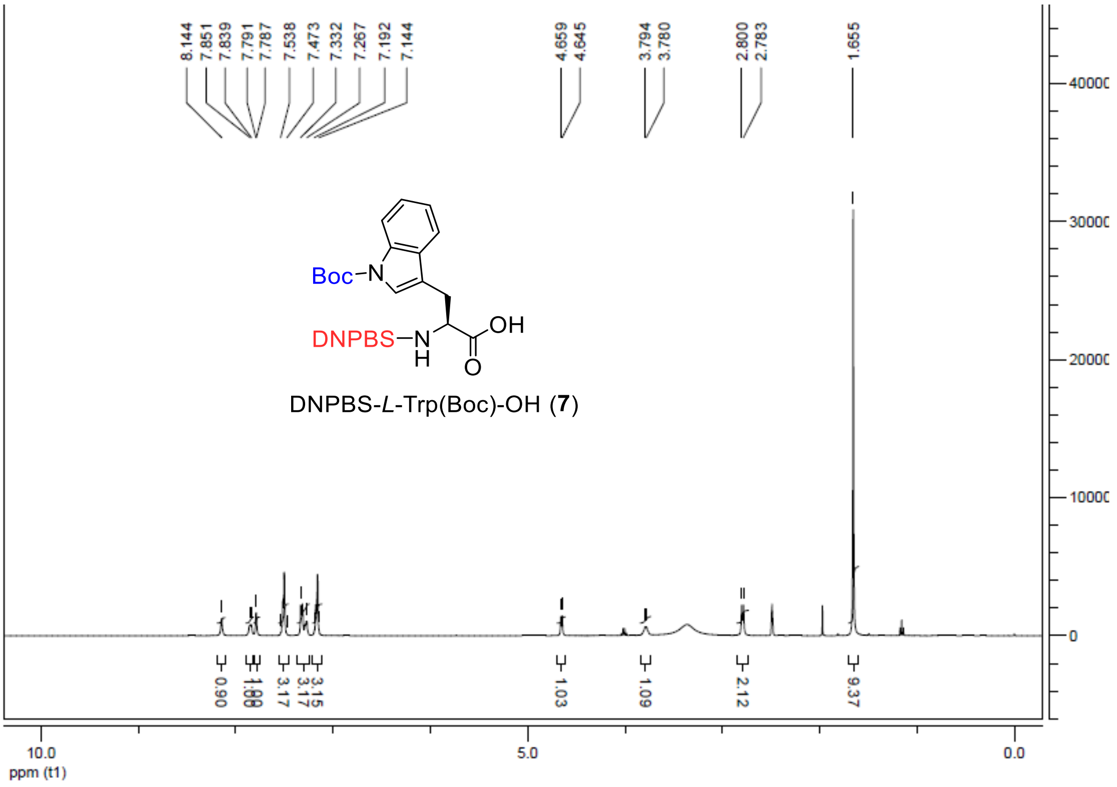


$^{13}$C NMR spectrum of **7** (in DMSO).

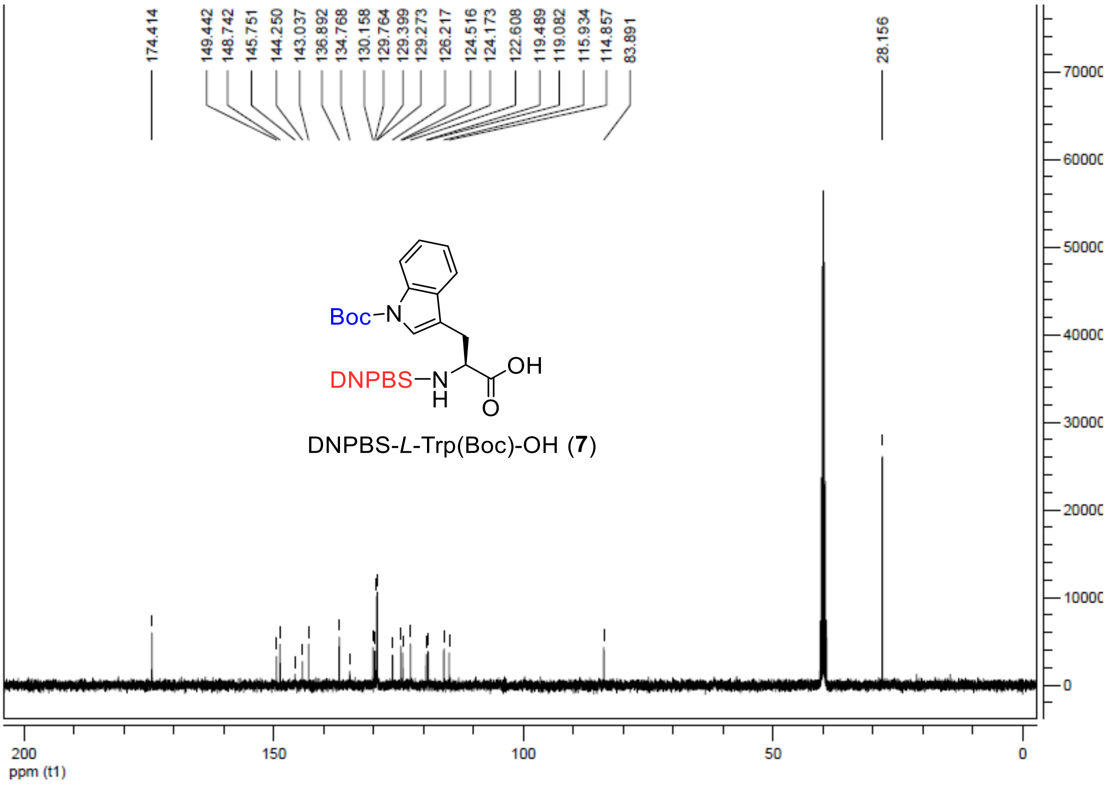

$^{1}$H NMR spectrum of **8** (in $CDCl_3$):

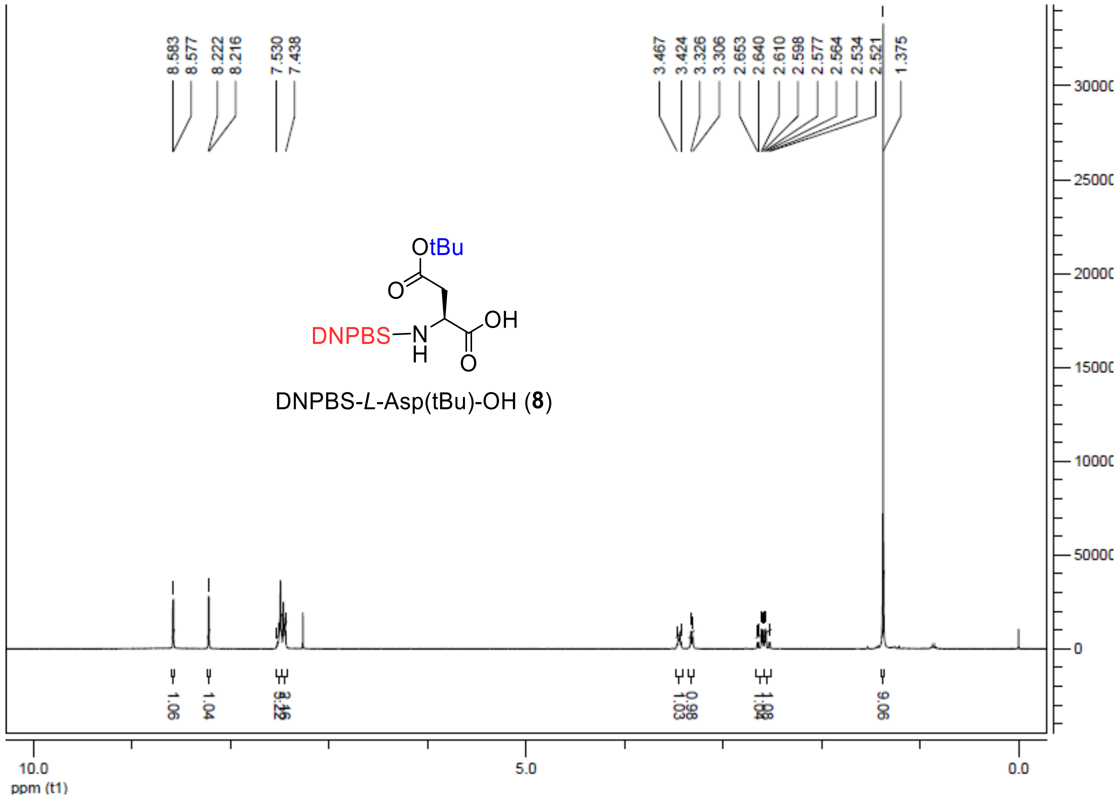


$^{13}$C NMR spectrum of **8** (in $CDCl_3$):

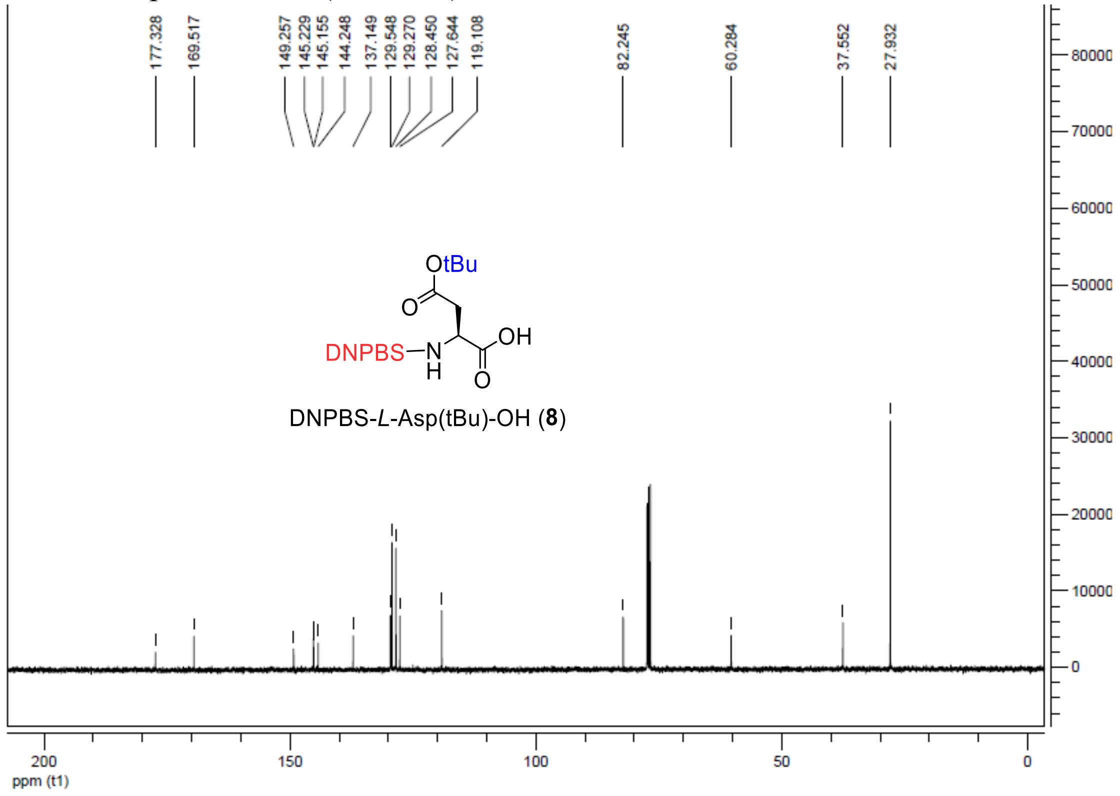

$^{1}H$ NMR spectrum of **9** (in $CDCl_3$):

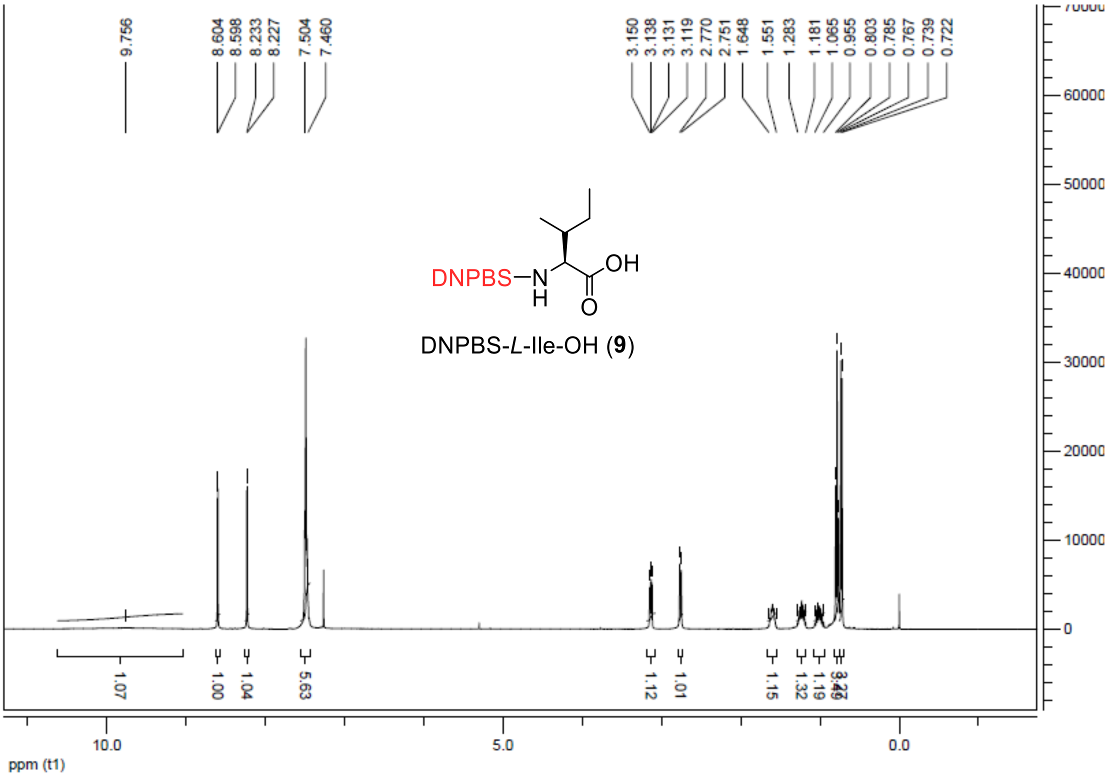


$^{13}C$ NMR spectrum of **9** (in $CDCl_3$):

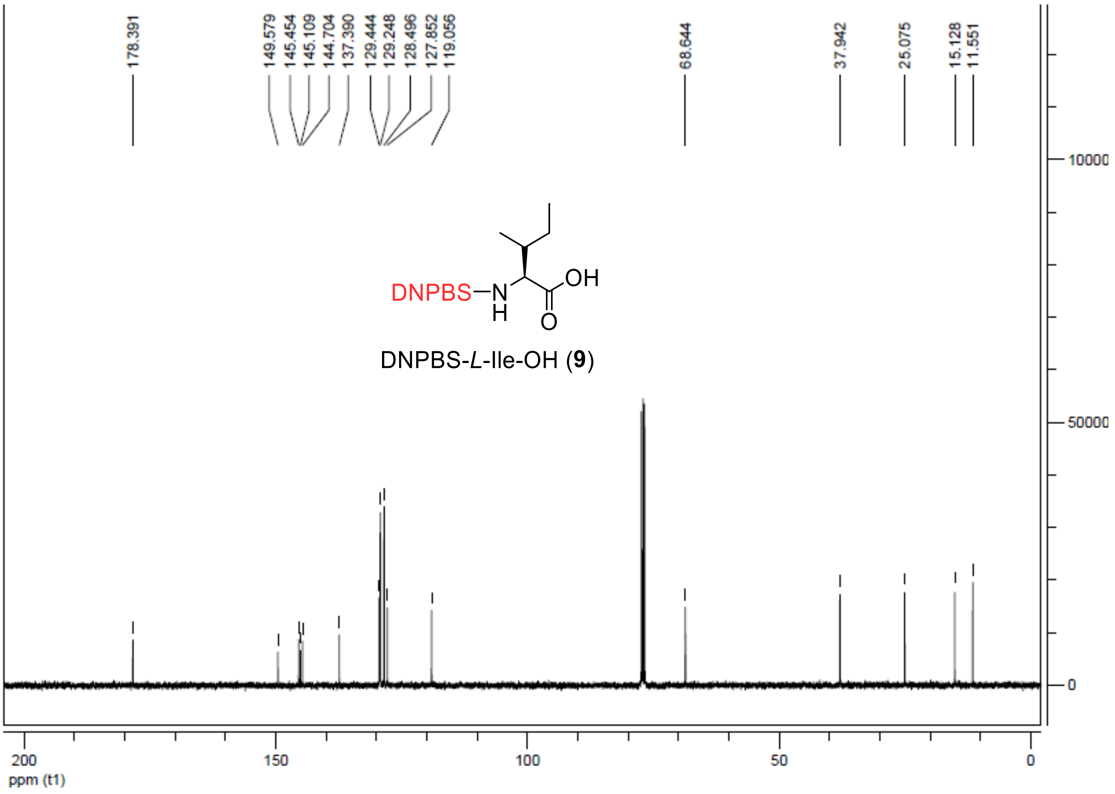

$^{1}$H NMR spectrum of **10** (in $CDCl_3$):

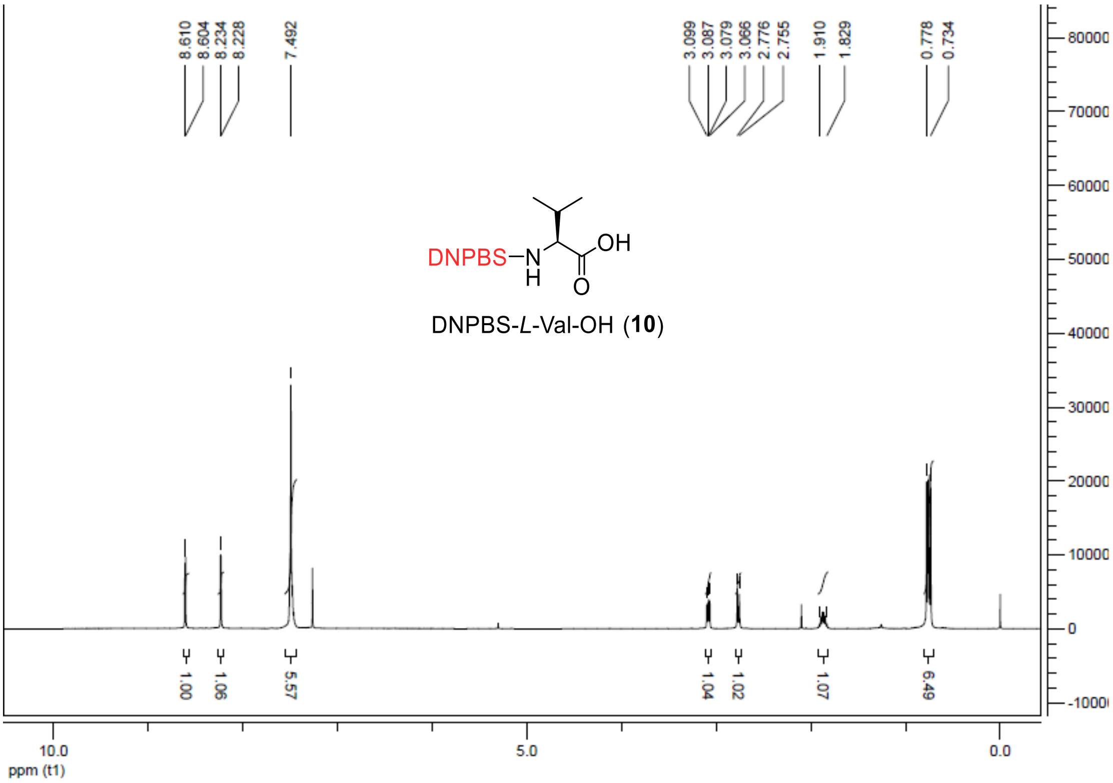


$^{13}$C NMR spectrum of **10** (in $CDCl_3$):

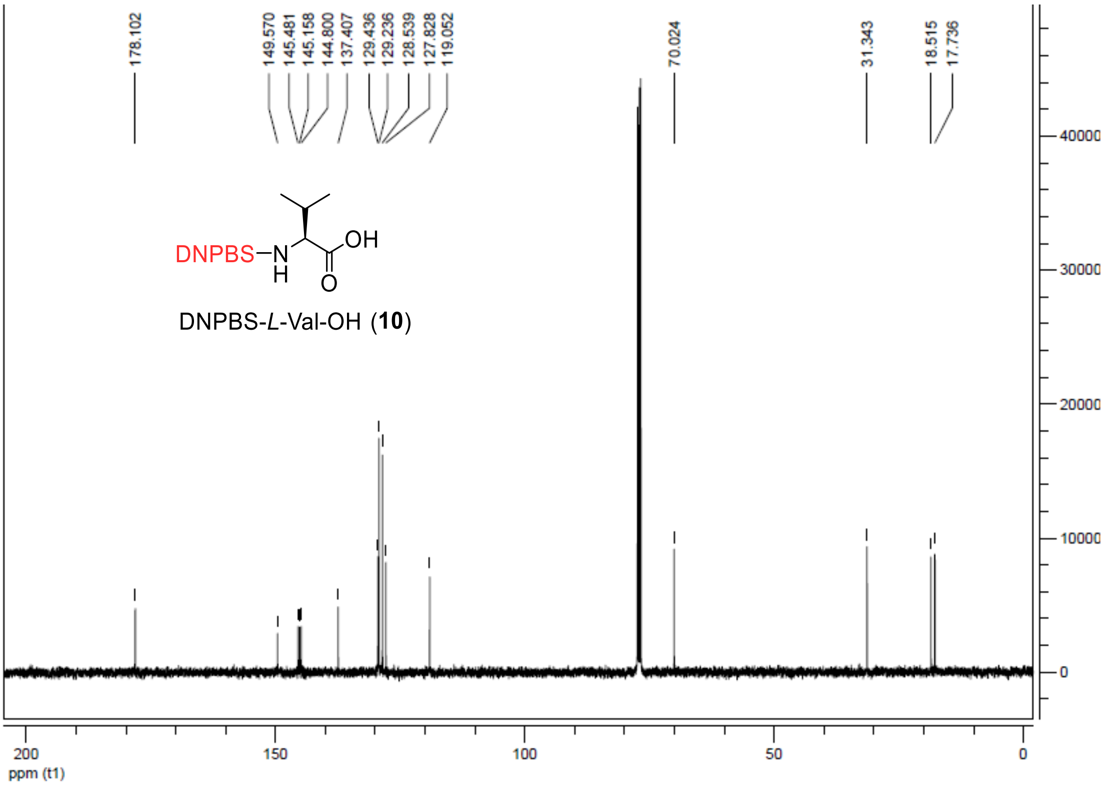

$^{1}$H NMR spectrum of **11** (in $CDCl_3$):

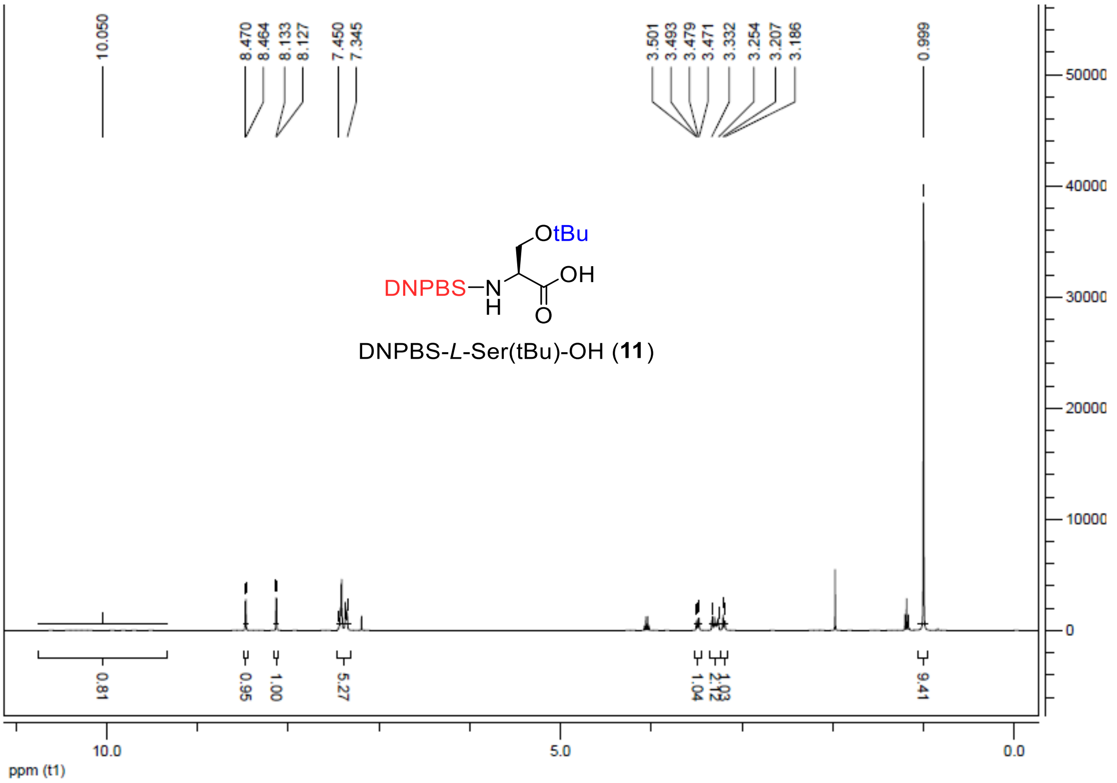


$^{13}$C NMR spectrum of **11** (in $CDCl_3$):

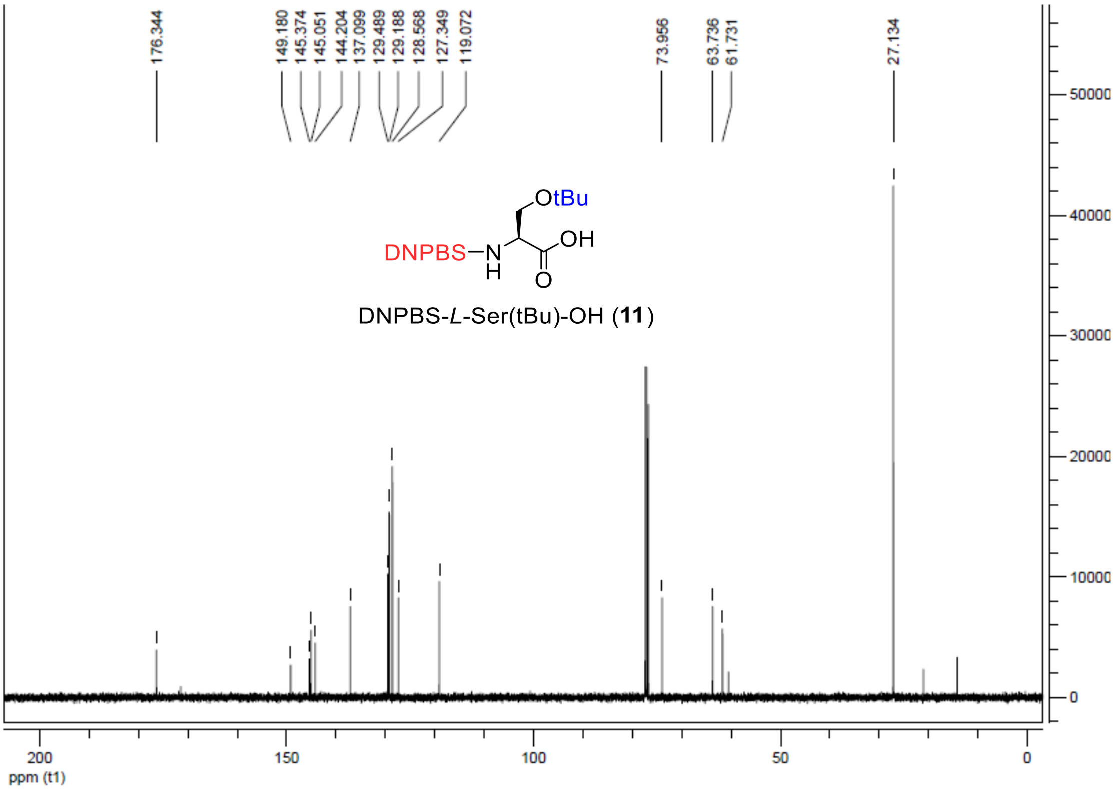

$^{1}$H NMR spectrum of **12** (in $CDCl_3$):

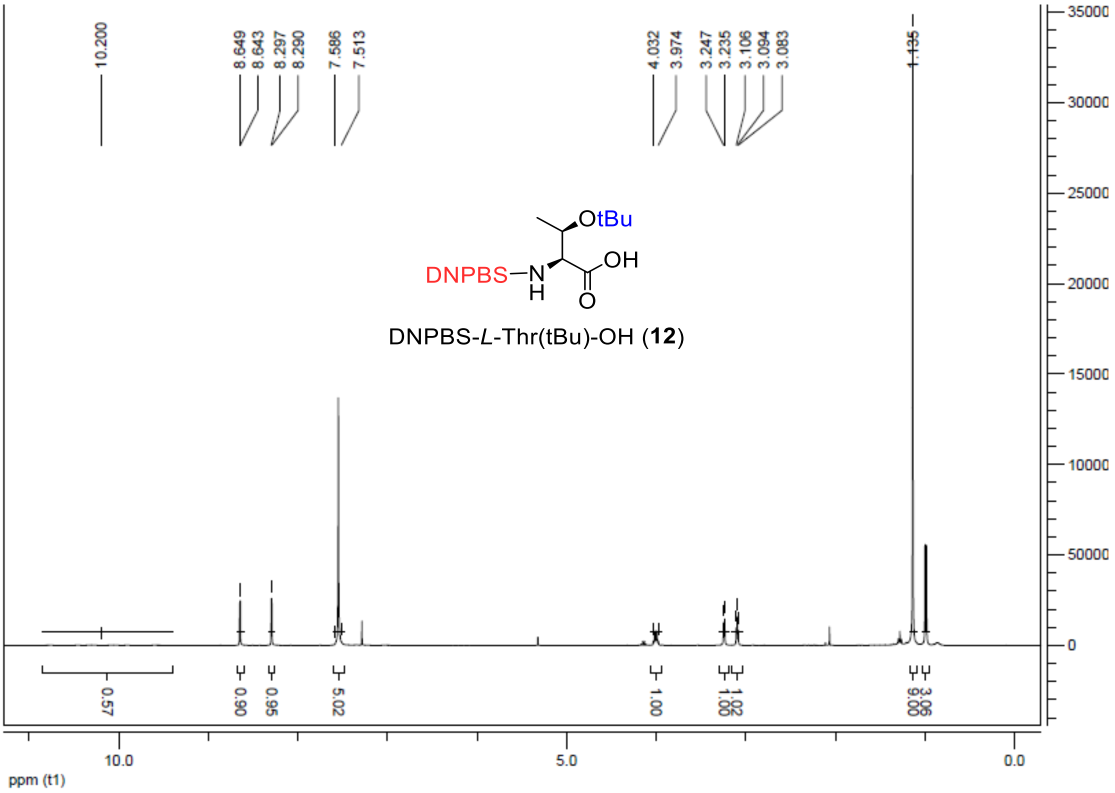


$^{13}$C NMR spectrum of **12** (in $CDCl_3$):

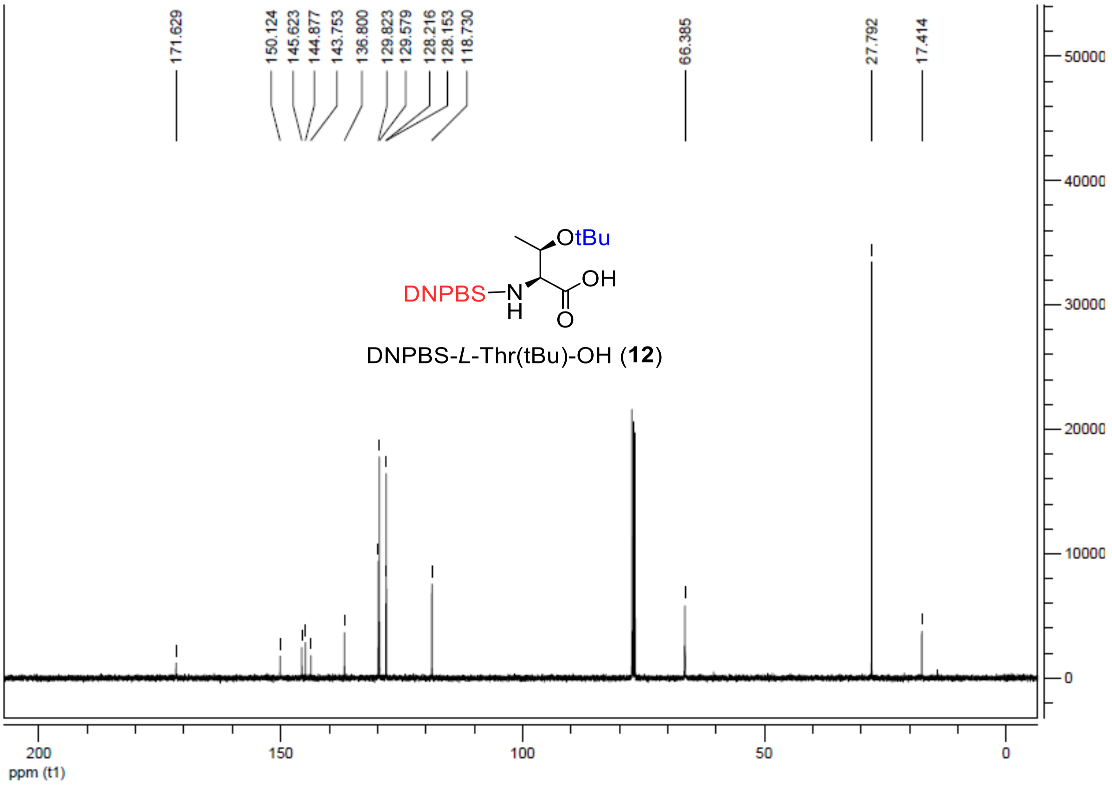

$^{1}$H NMR spectrum of **13** (in $CDCl_3$):

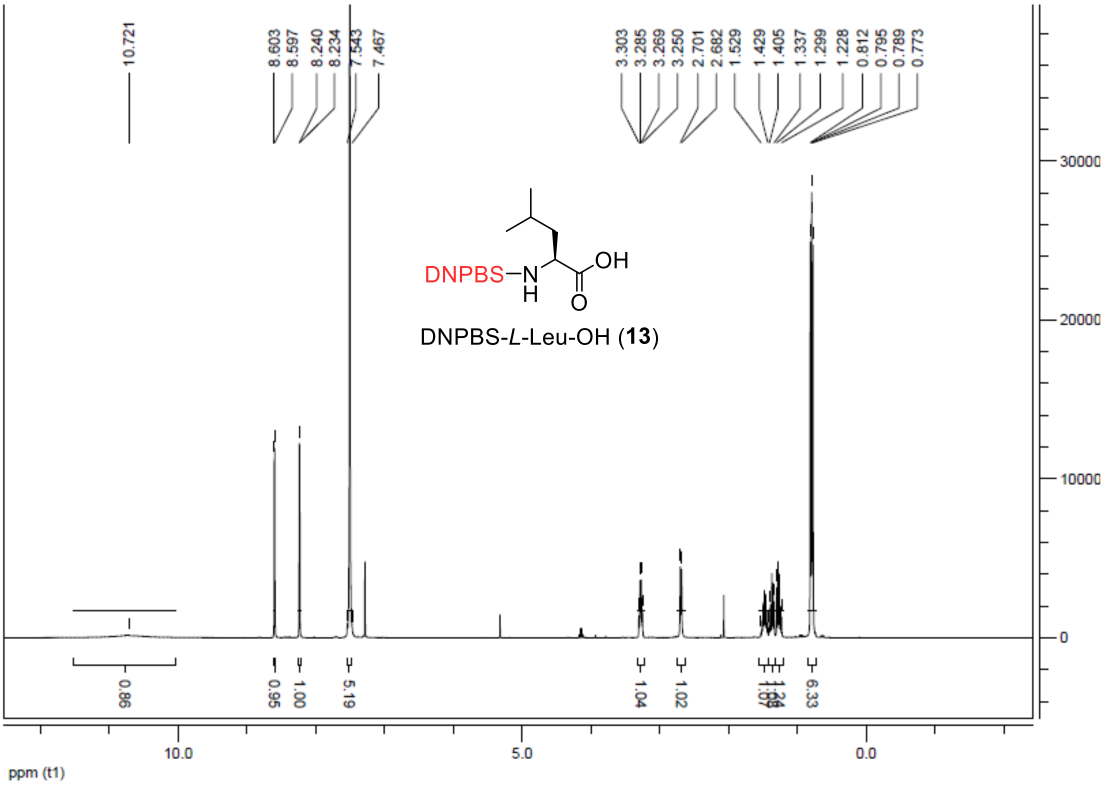


$^{13}$C NMR spectrum of **13** (in $CDCl_3$):

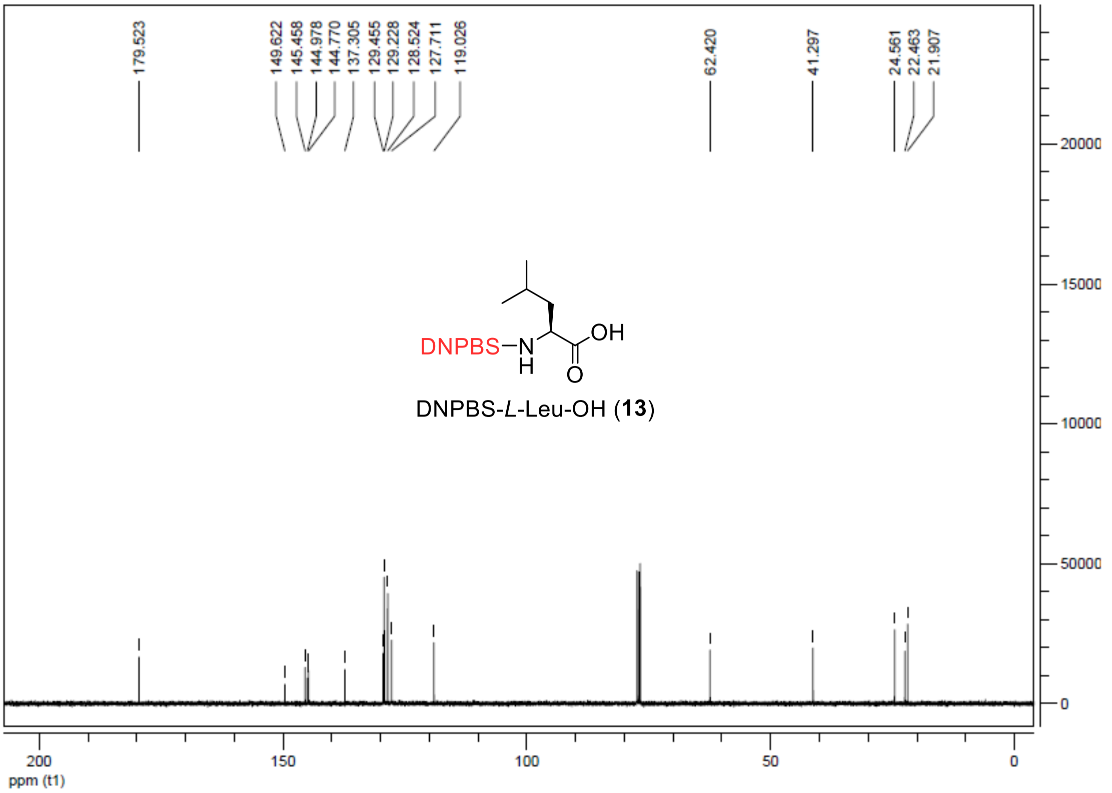

$^1$H NMR spectrum of **14** (in $CDCl_3$):

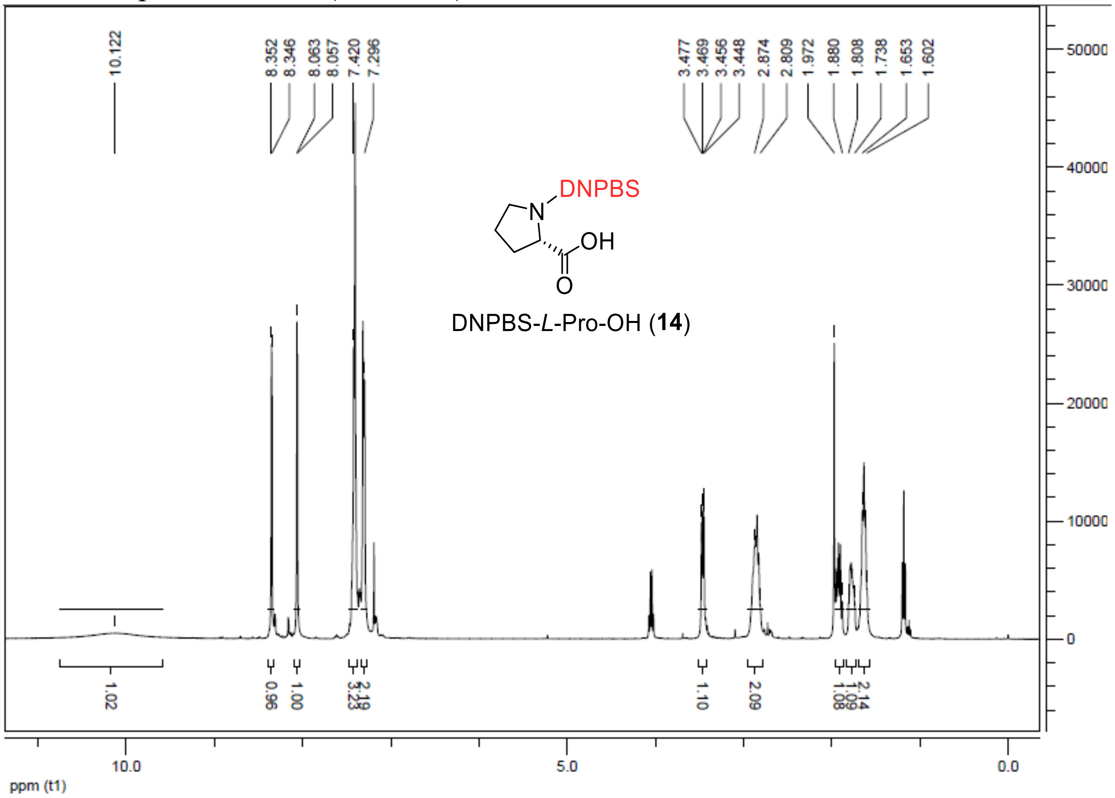


$^{13}$C NMR spectrum of **14** (in $CDCl_3$):

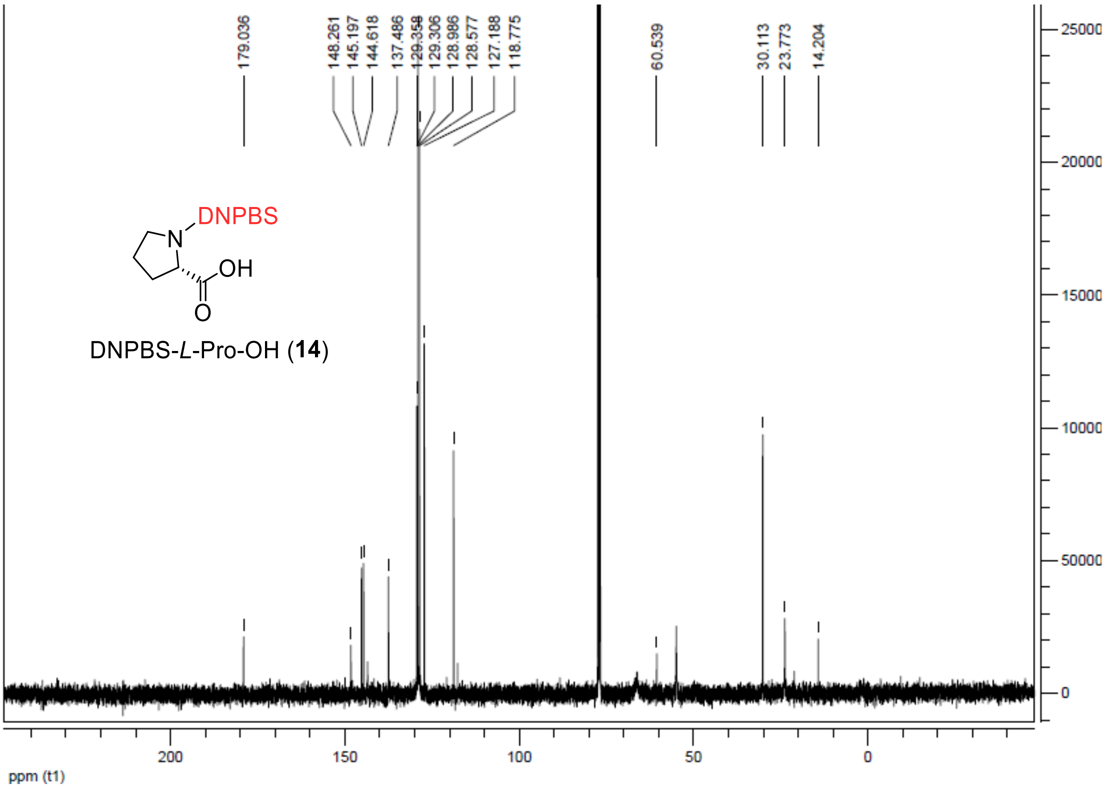

$^{1}H$ NMR spectrum of **15** (in $CDCl_3$):

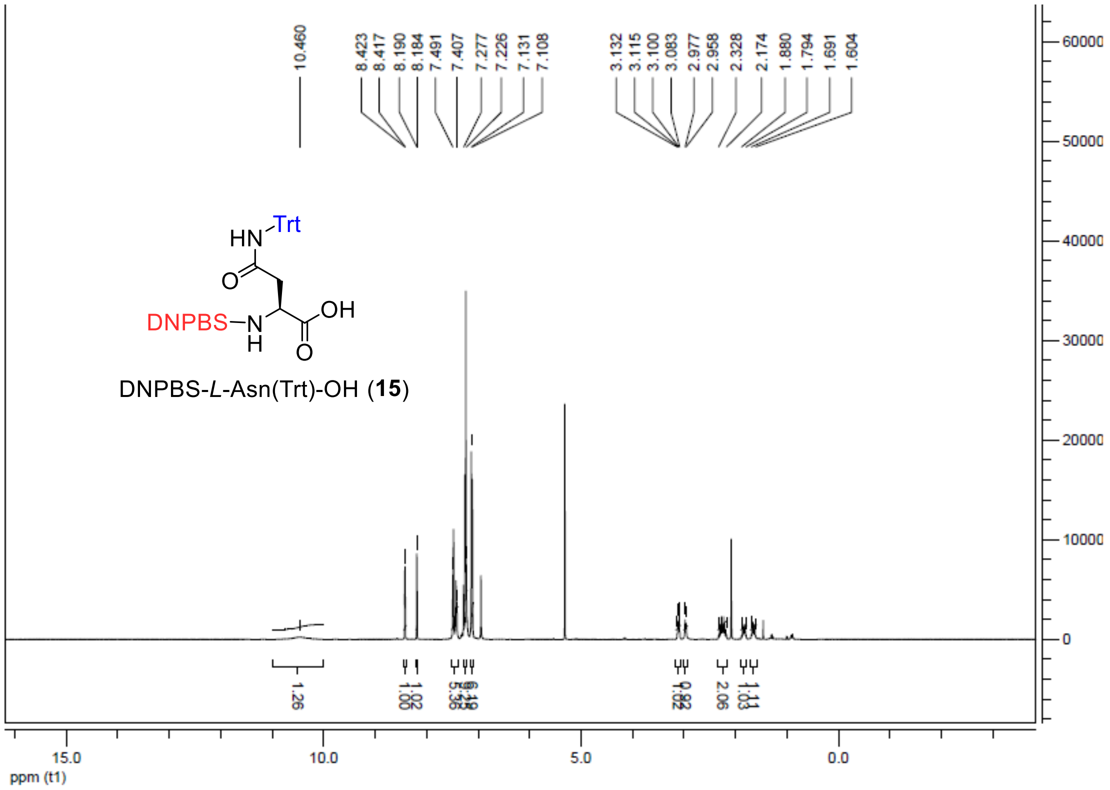


$^{13}C$ NMR spectrum of **15** (in $CDCl_3$):

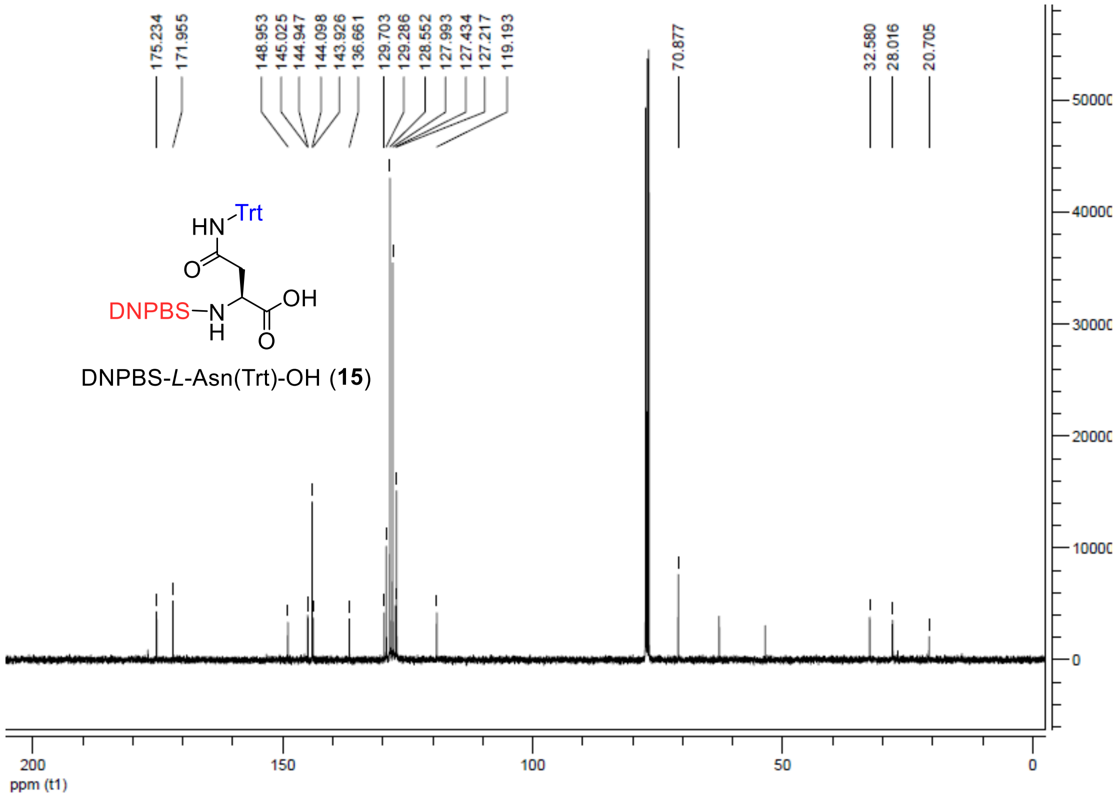

$^{1}$H NMR spectrum of **16** (in $CDCl_3$):

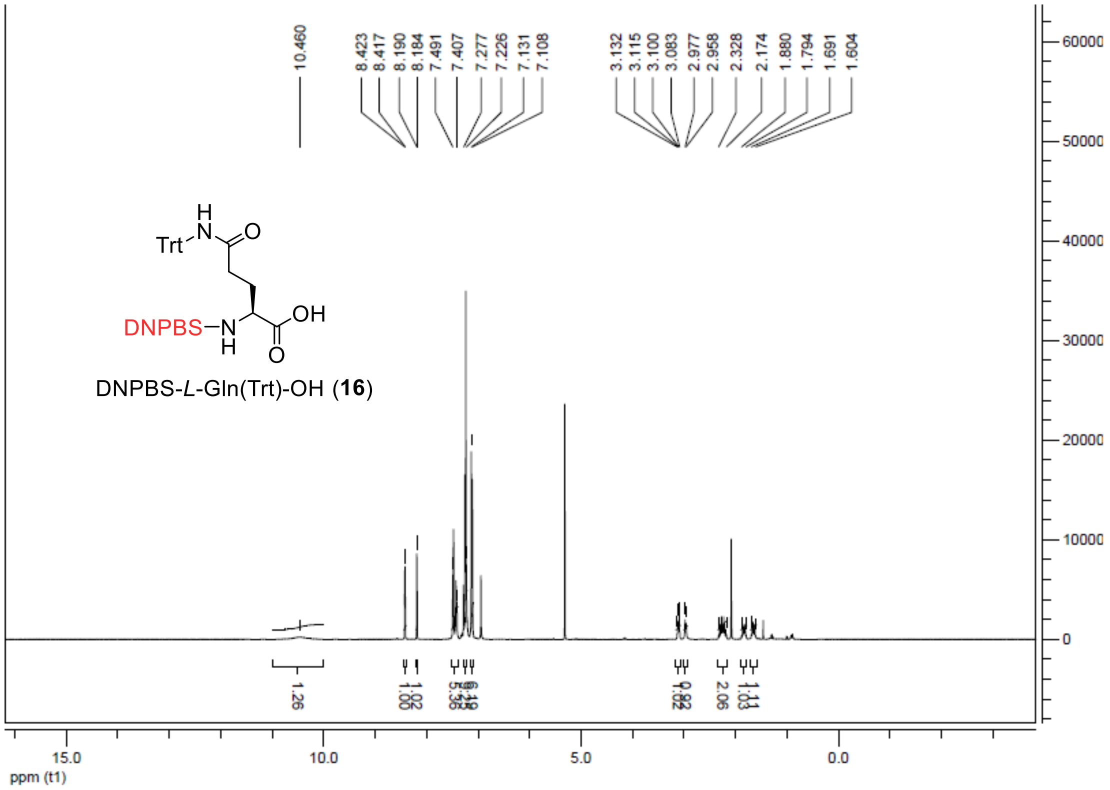


$^{13}$C NMR spectrum of **16** (in $CDCl_3$).

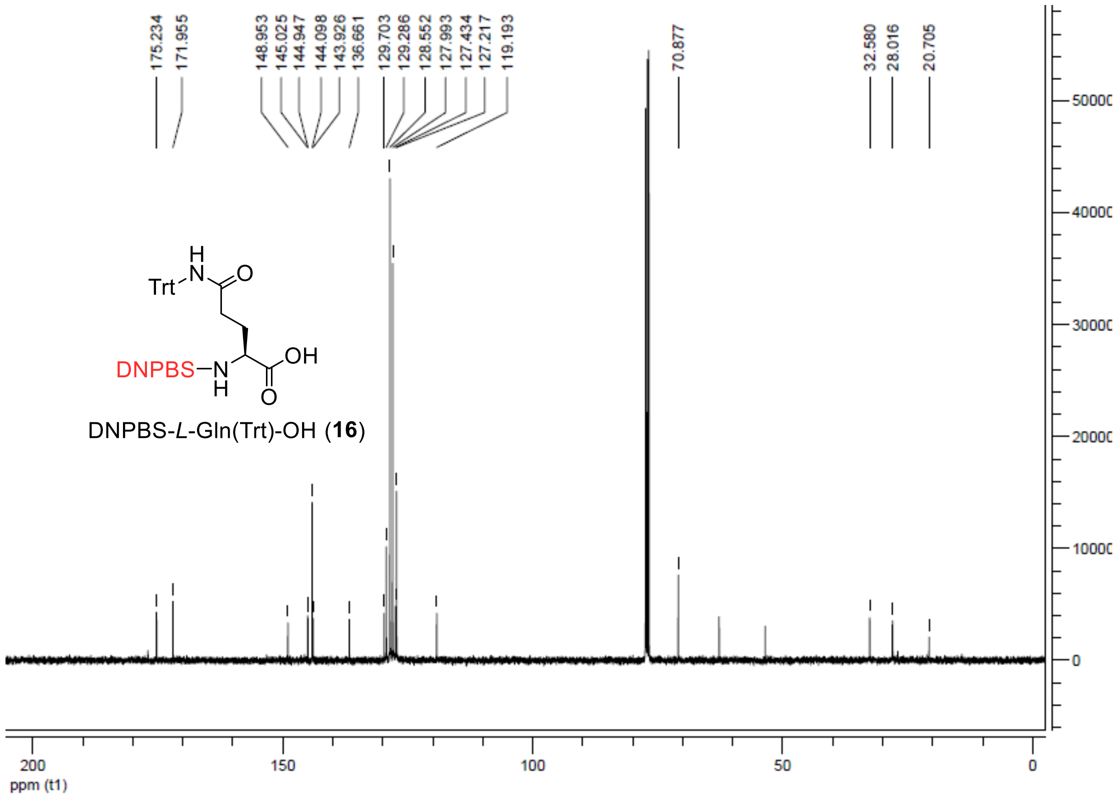

$^{1}H$ NMR spectrum of **17** (in $CDCl_3$):

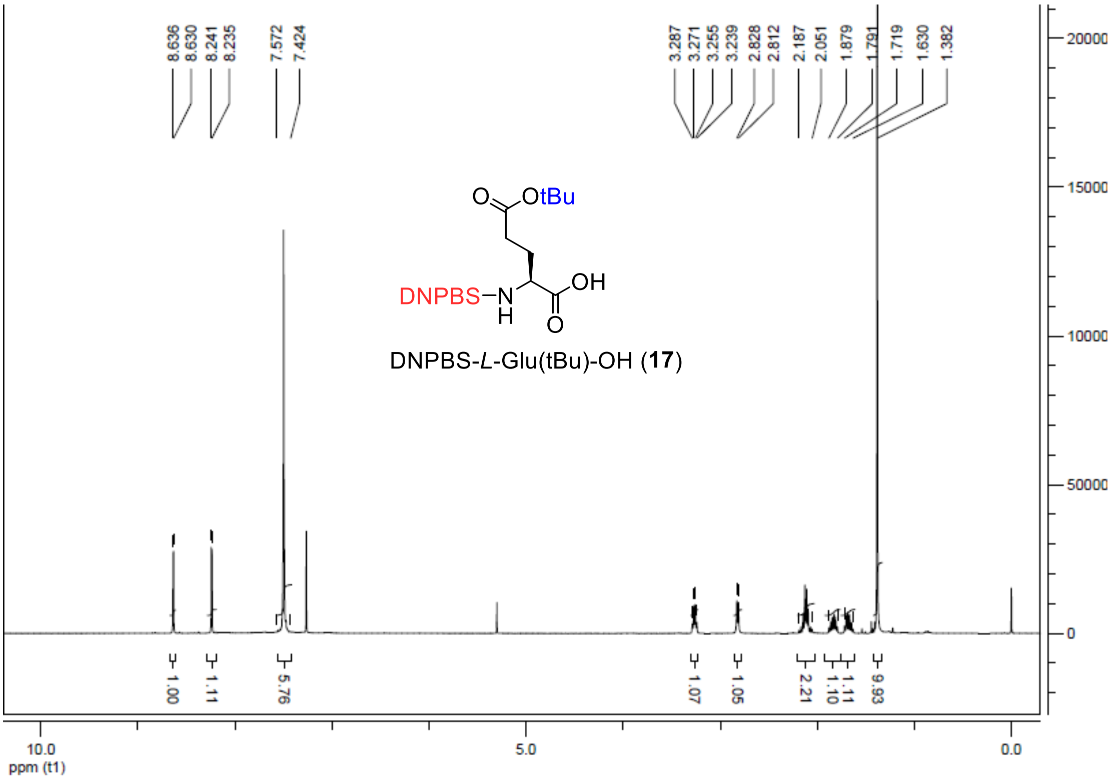


$^{13}C$ NMR spectrum of **17** (in $CDCl_3$):

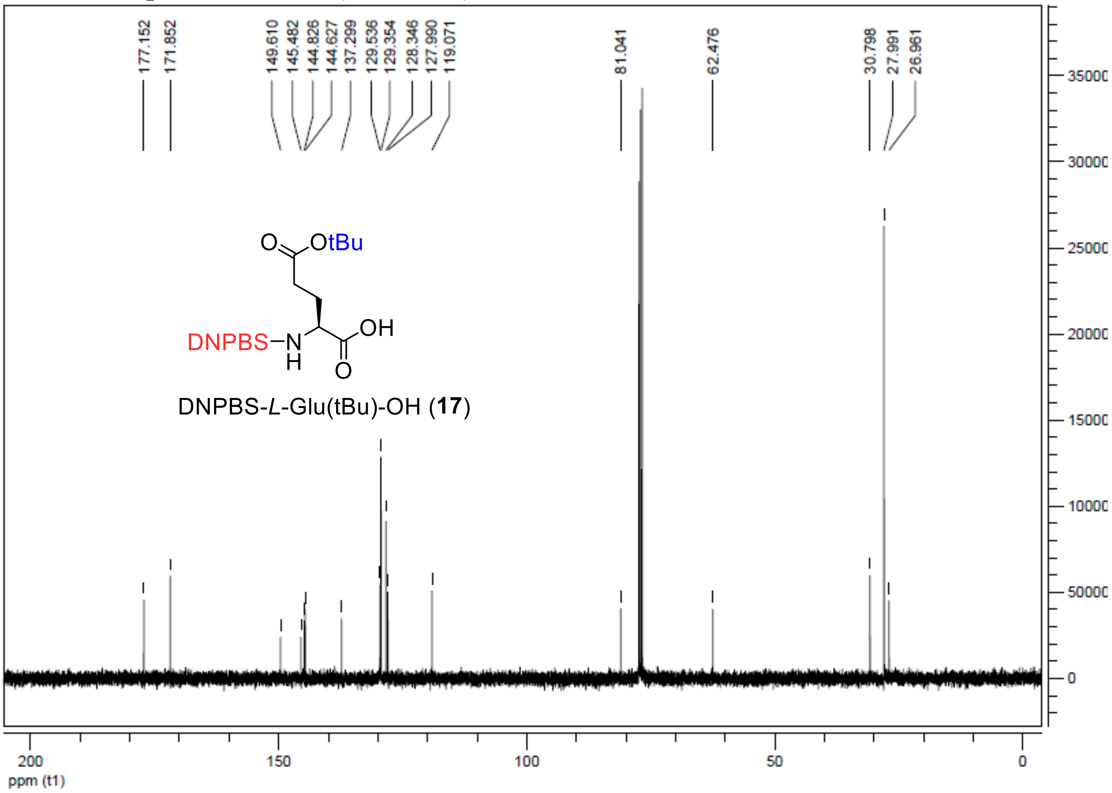

$^{1}H$ NMR spectrum of **18** (in $CDCl_3$):

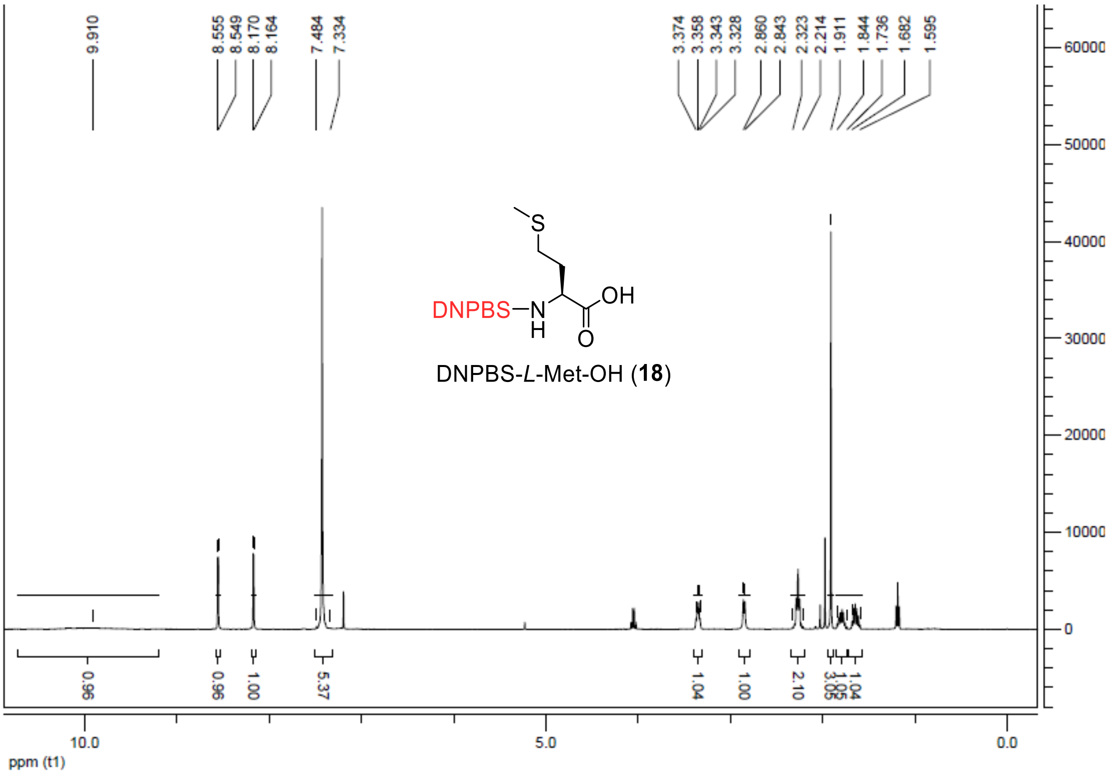


$^{13}C$ NMR spectrum of **18** (in $CDCl_3$).:

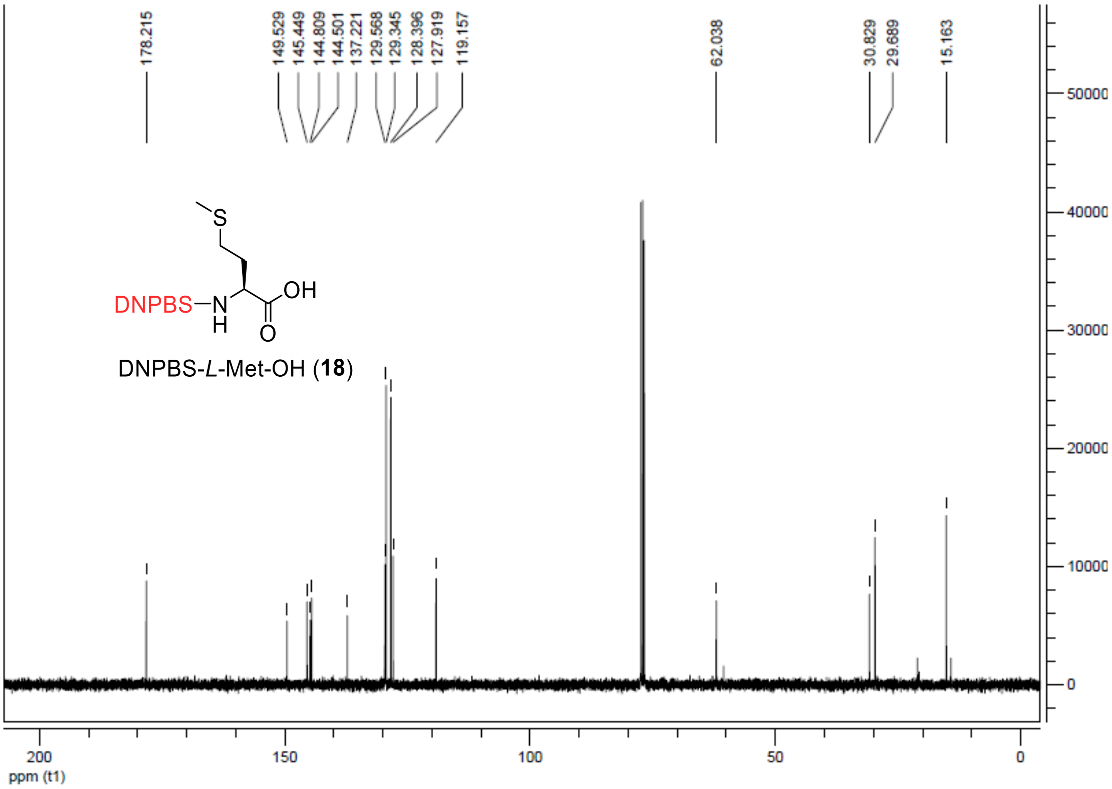

$^{1}$H NMR spectrum of **19** (in DMSO):

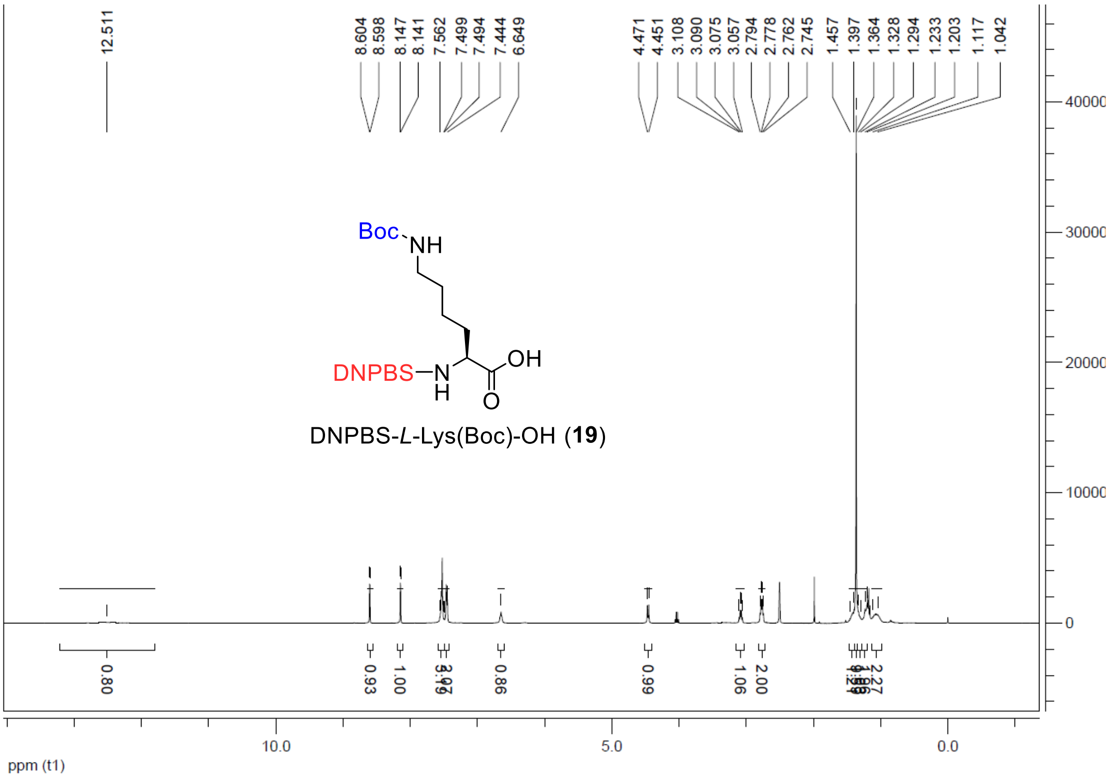


$^{13}$C NMR spectrum of **19** (in DMSO).

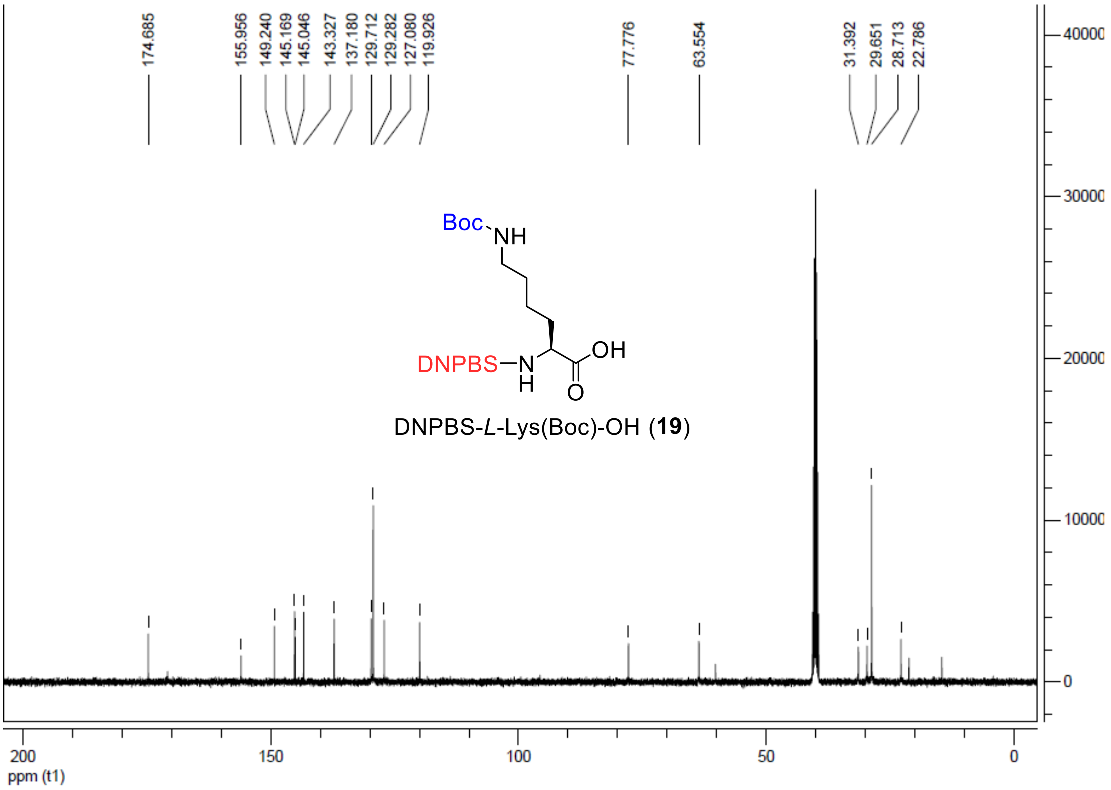

$^{1}$H NMR spectrum of **20** (in $CDCl_3$):

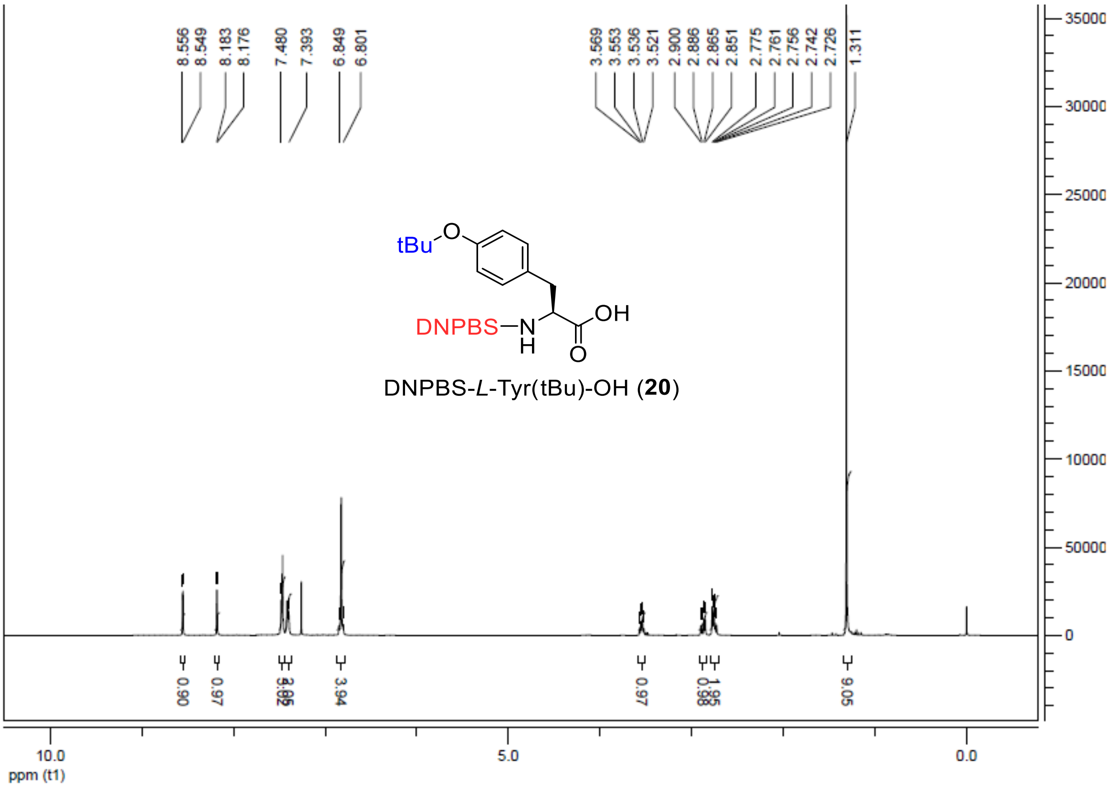


$^{13}$C NMR spectrum of **20** (in $CDCl_3$).

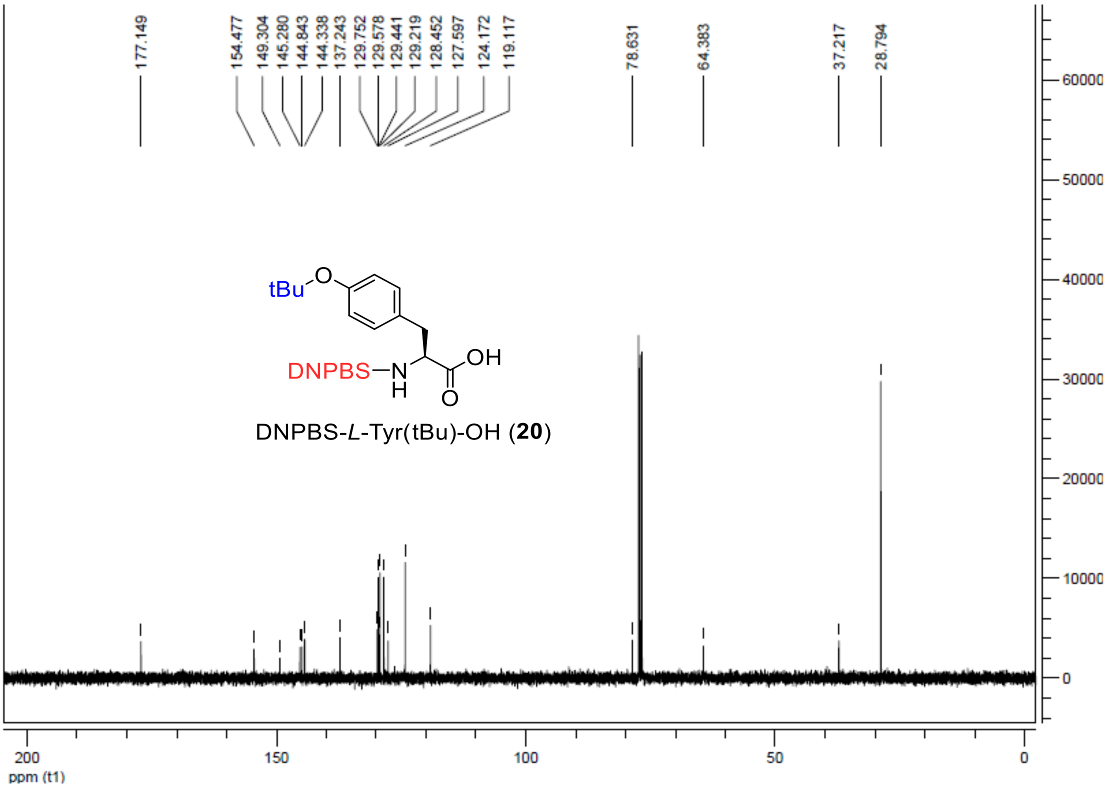

$^{1}$H NMR spectrum of **21** (in DMSO):

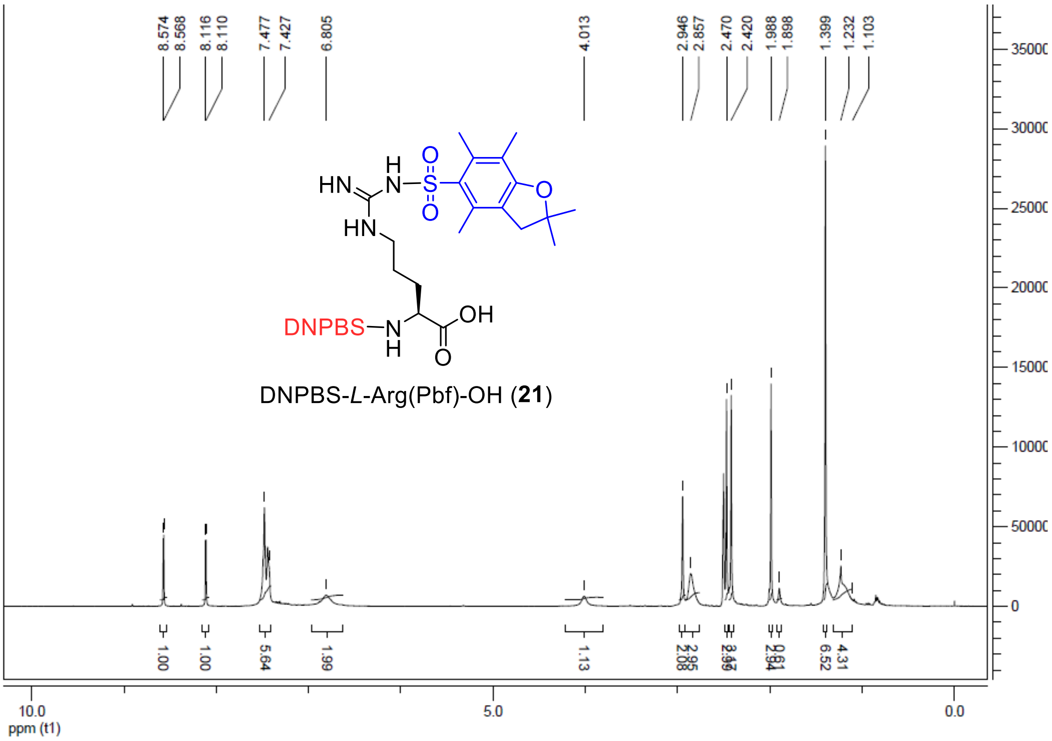


$^{13}$C NMR spectrum of **21** (in DMSO):

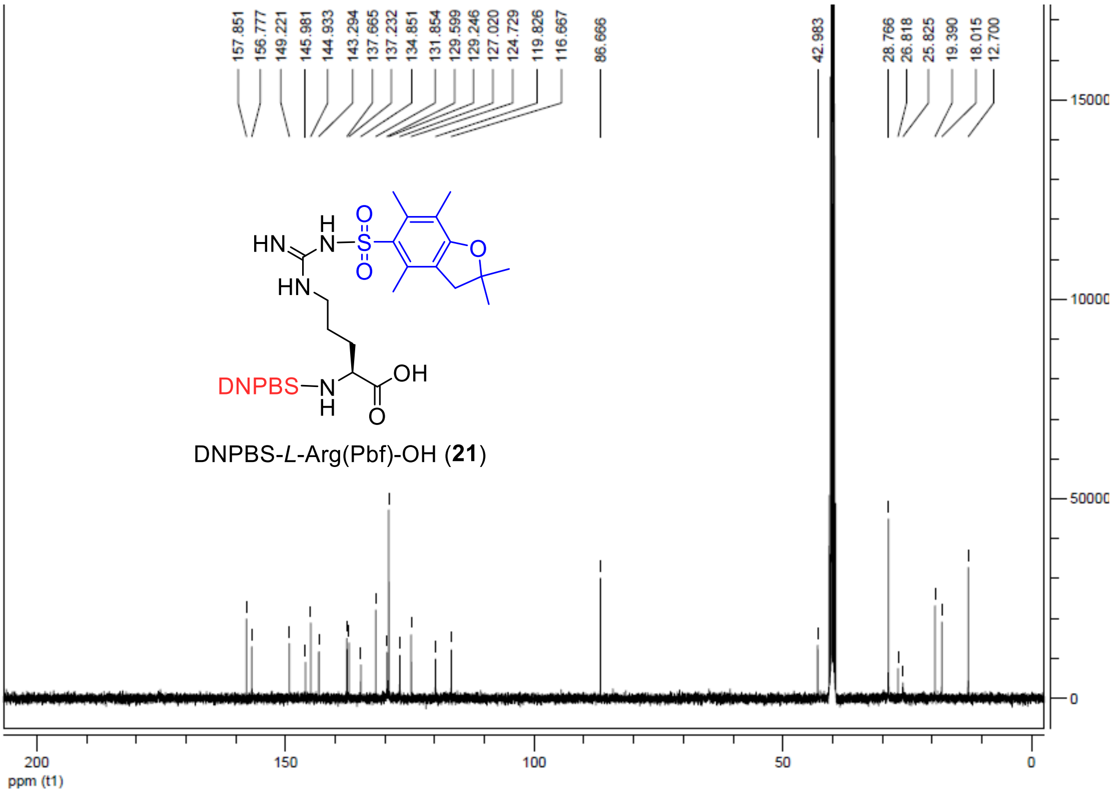

$^{1}$H NMR spectrum of **22** (in $CDCl_3$):

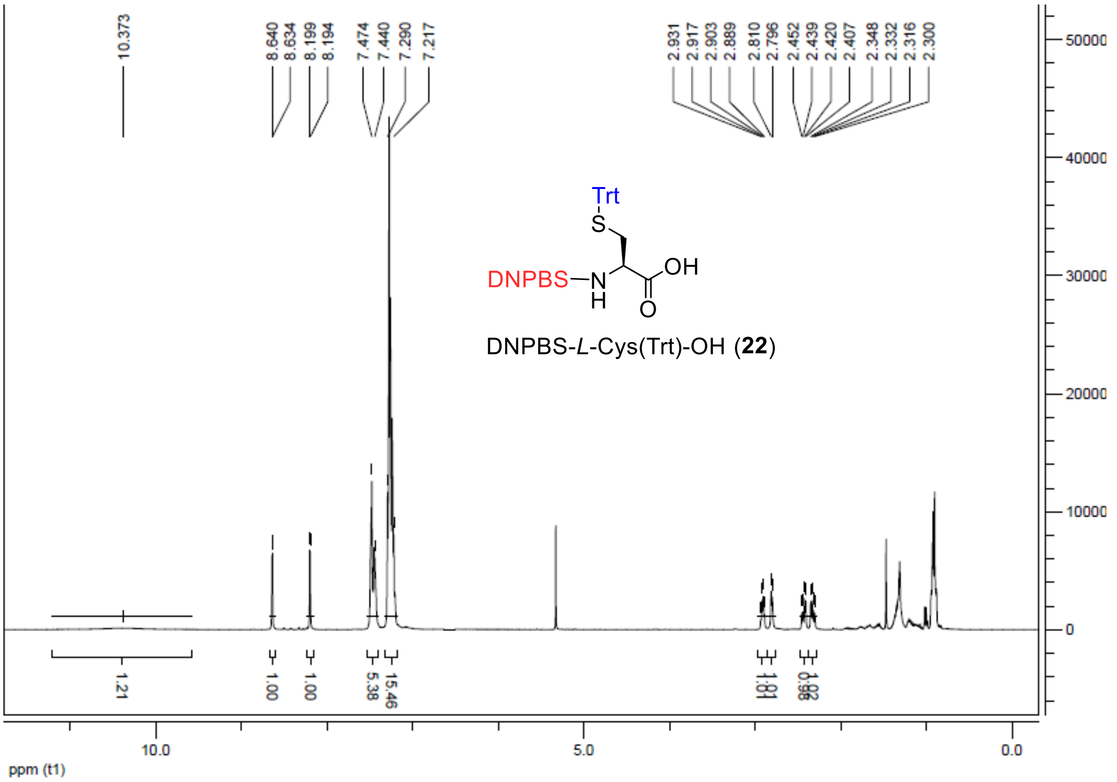


$^{13}$C NMR spectrum of **22** (in $CDCl_3$):

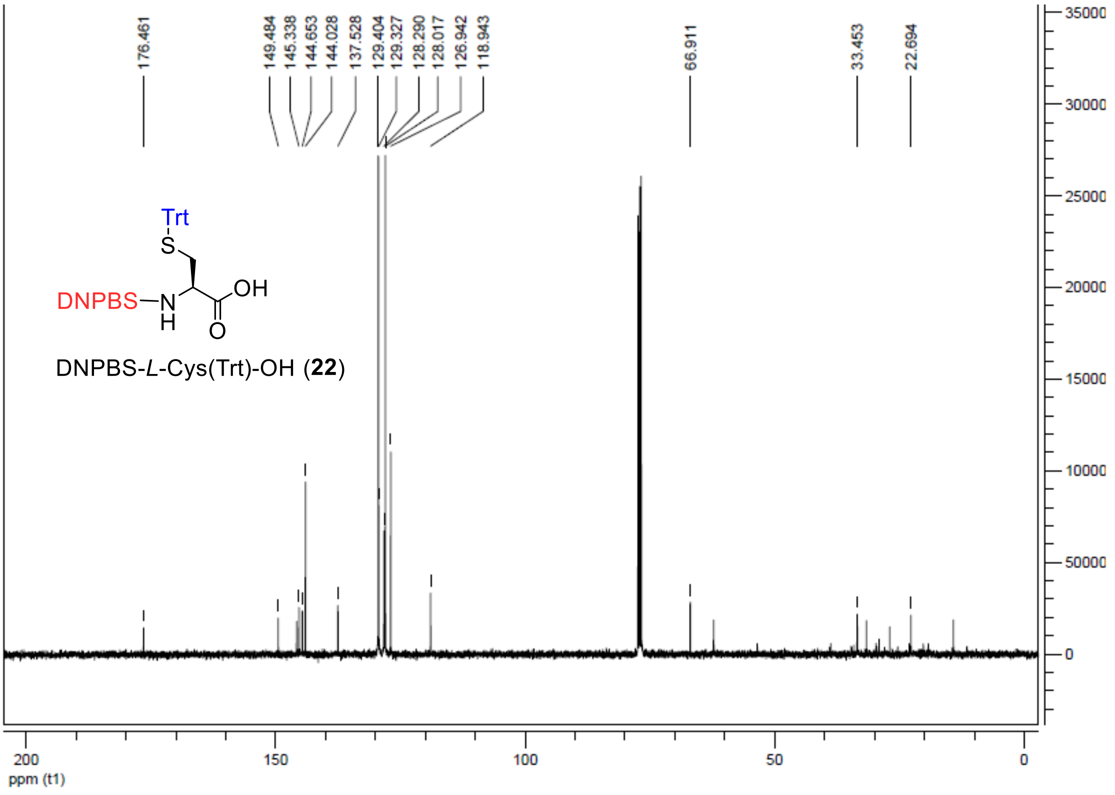

$^{1}$H NMR spectrum of **23** (in $CDCl_3$):

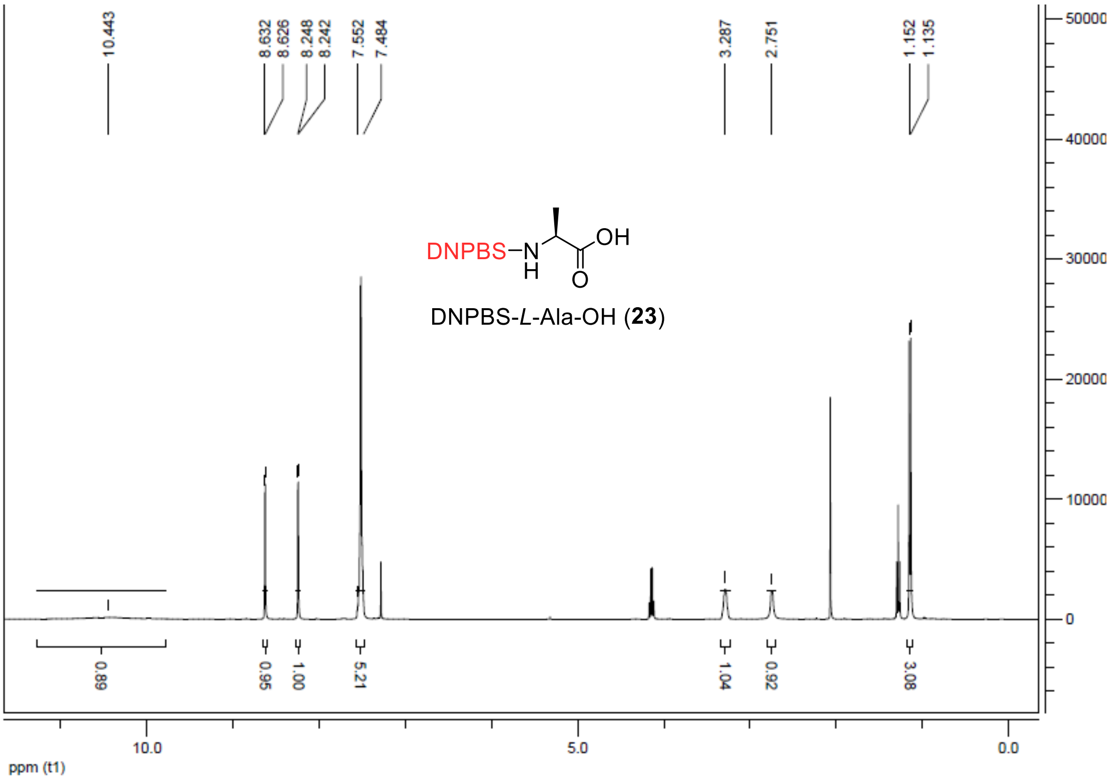


$^{13}$C NMR spectrum of **23** (in $CDCl_3$).

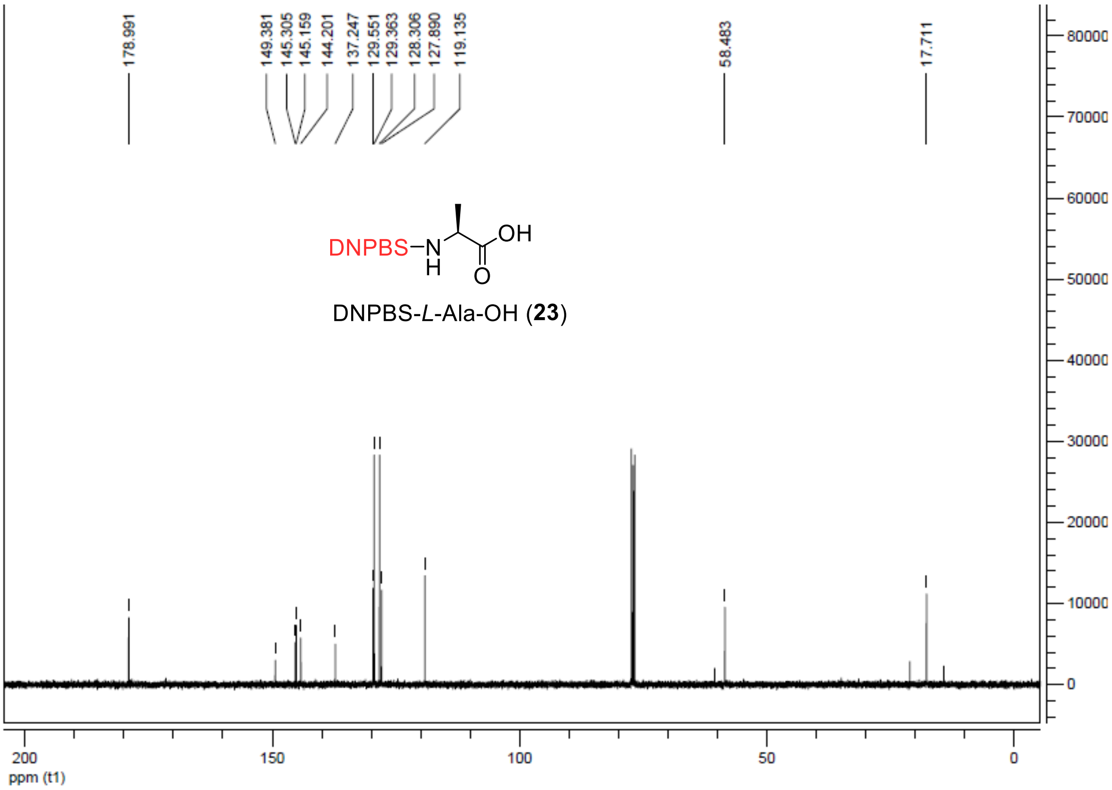

$^{1}$H NMR spectrum of **24** (in $CDCl_3$):

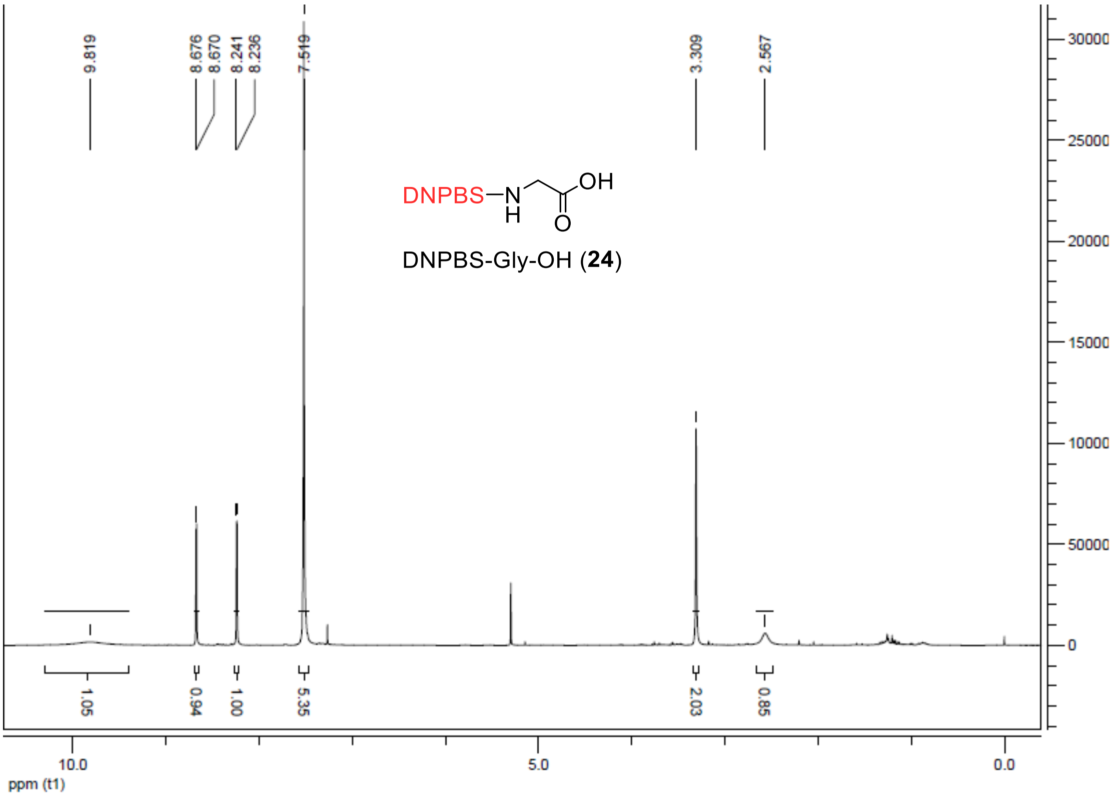


$^{13}$C NMR spectrum of **24** (in $CDCl_3$):

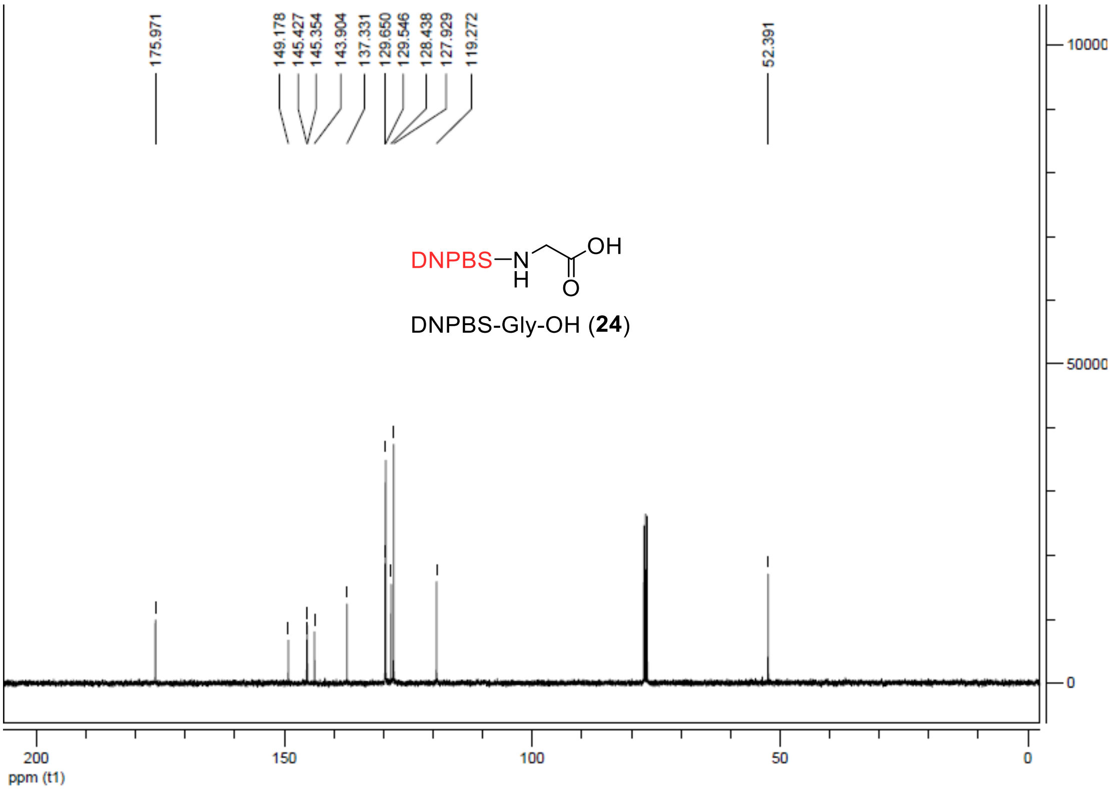

$^1$H NMR spectrum of **26** (in $CDCl_3$):

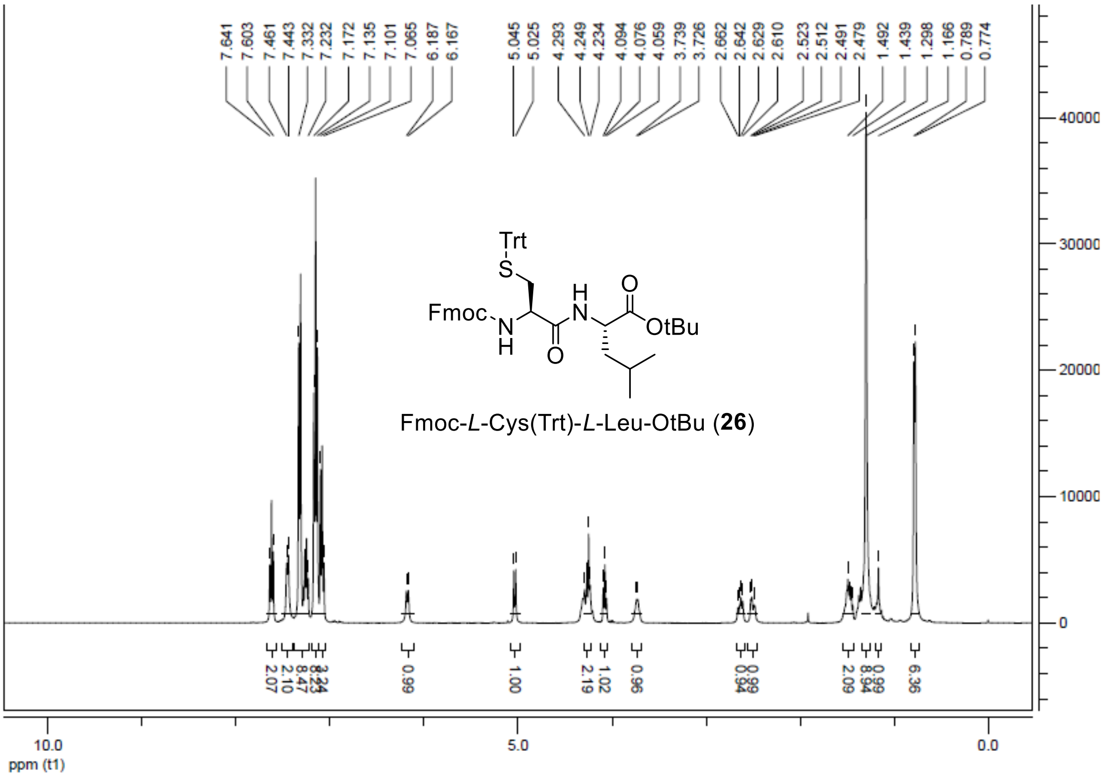


$^{13}$C NMR spectrum of **26** (in $CDCl_3$):

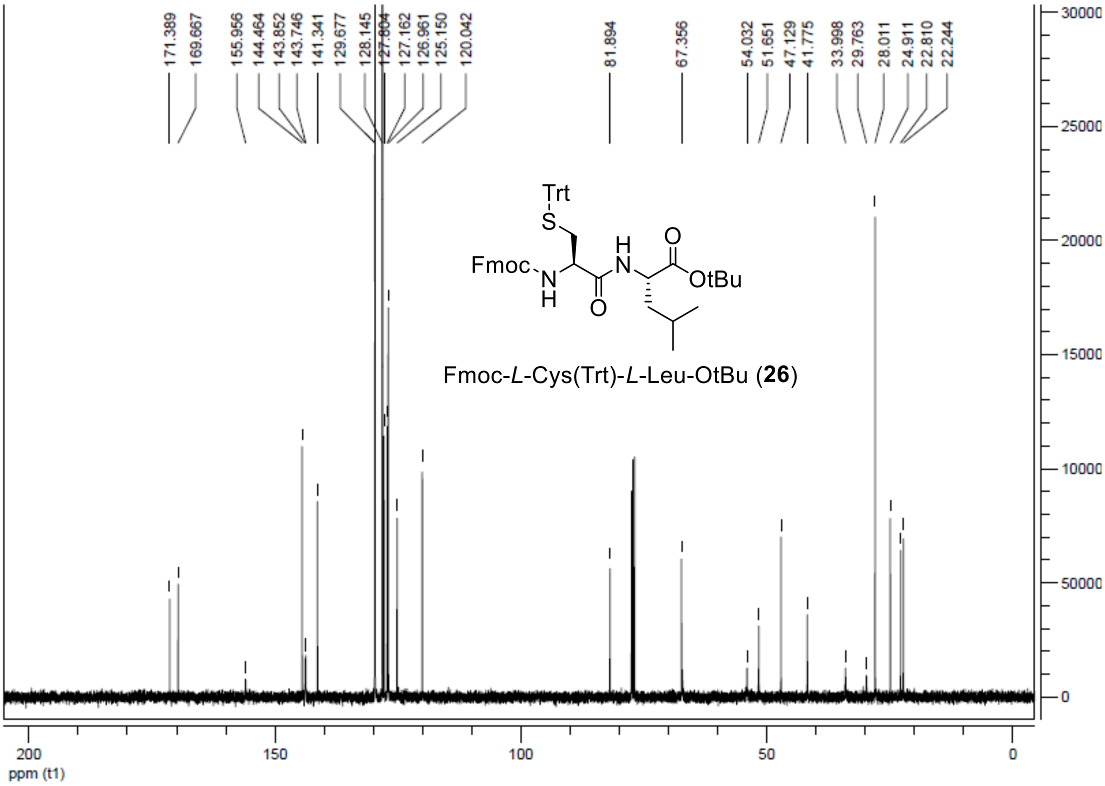

$^{1}$H NMR spectrum of **28** (in $CDCl_3$):

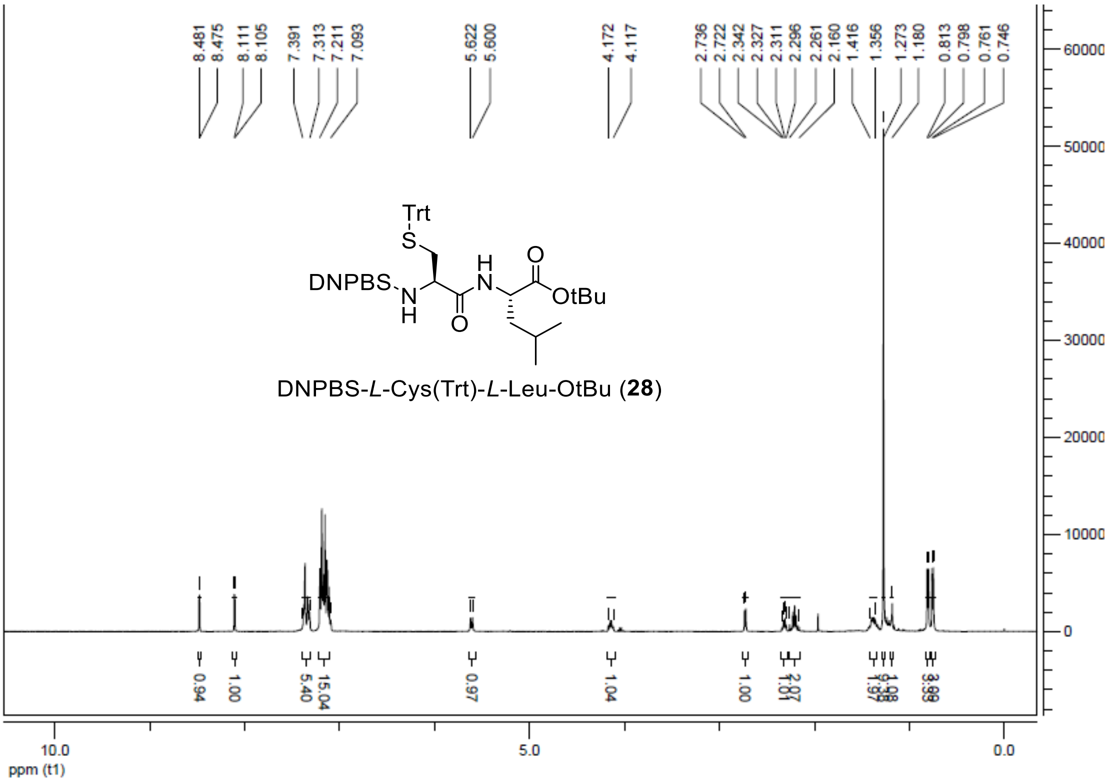


$^{13}$C NMR spectrum of **28** (in $CDCl_3$):

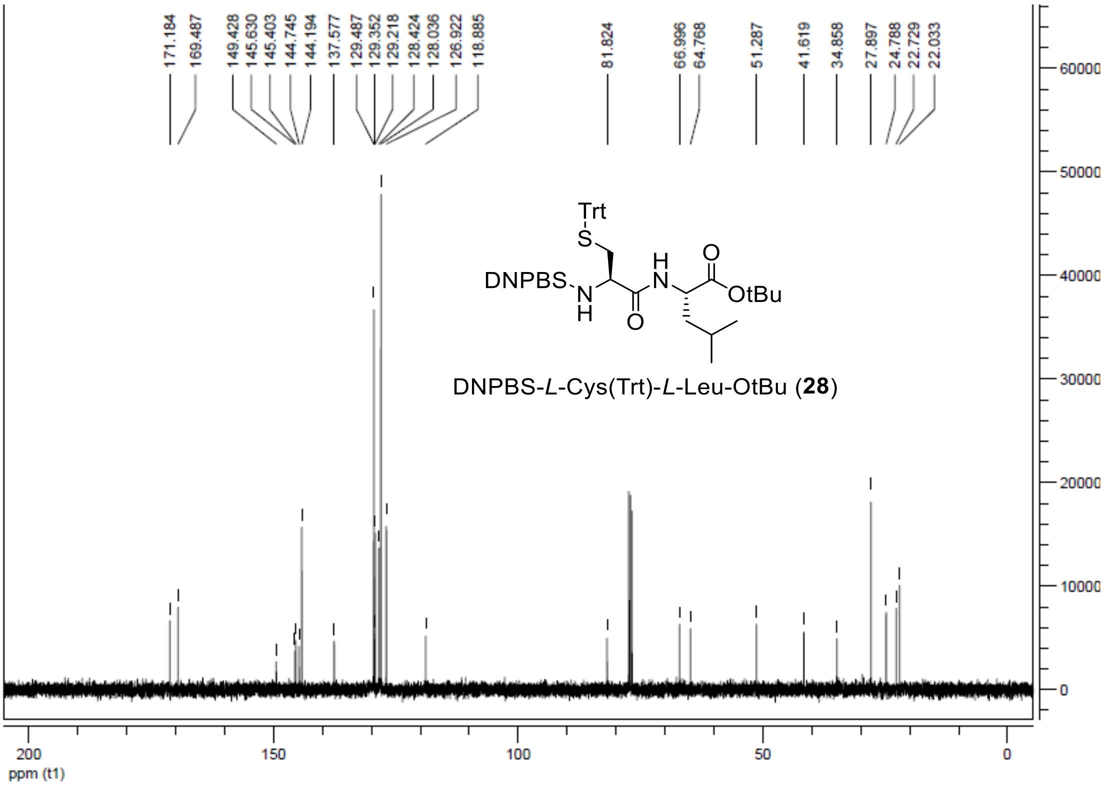

$^{1}$H NMR spectrum of **30** (in $CDCl_3$):

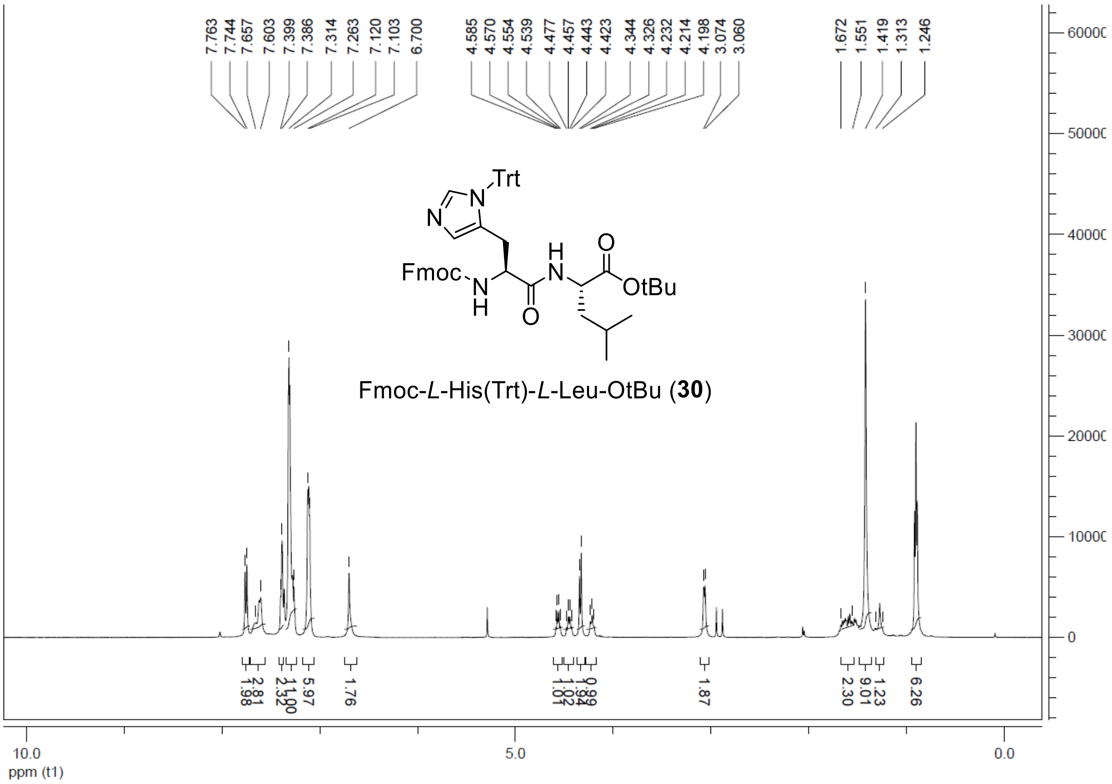


$^{13}$C NMR spectrum of **30** (in $CDCl_3$):

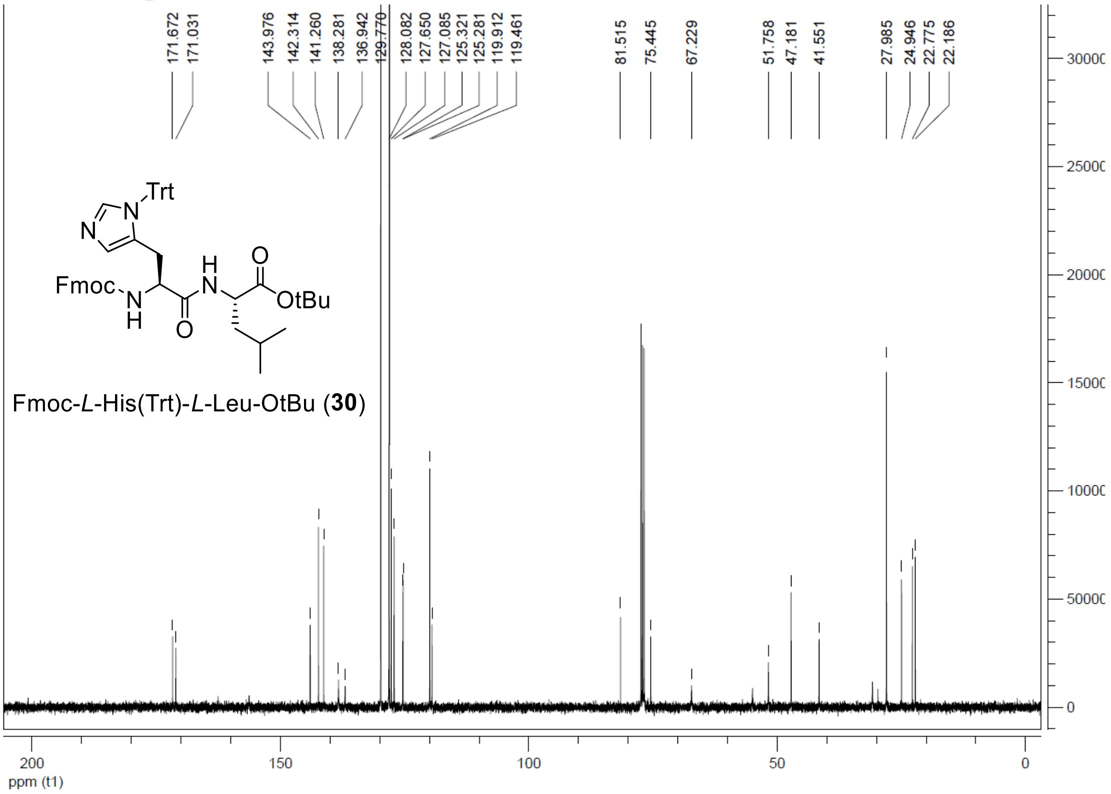

$^1H$ NMR spectrum of **32** (in $CDCl_3$):

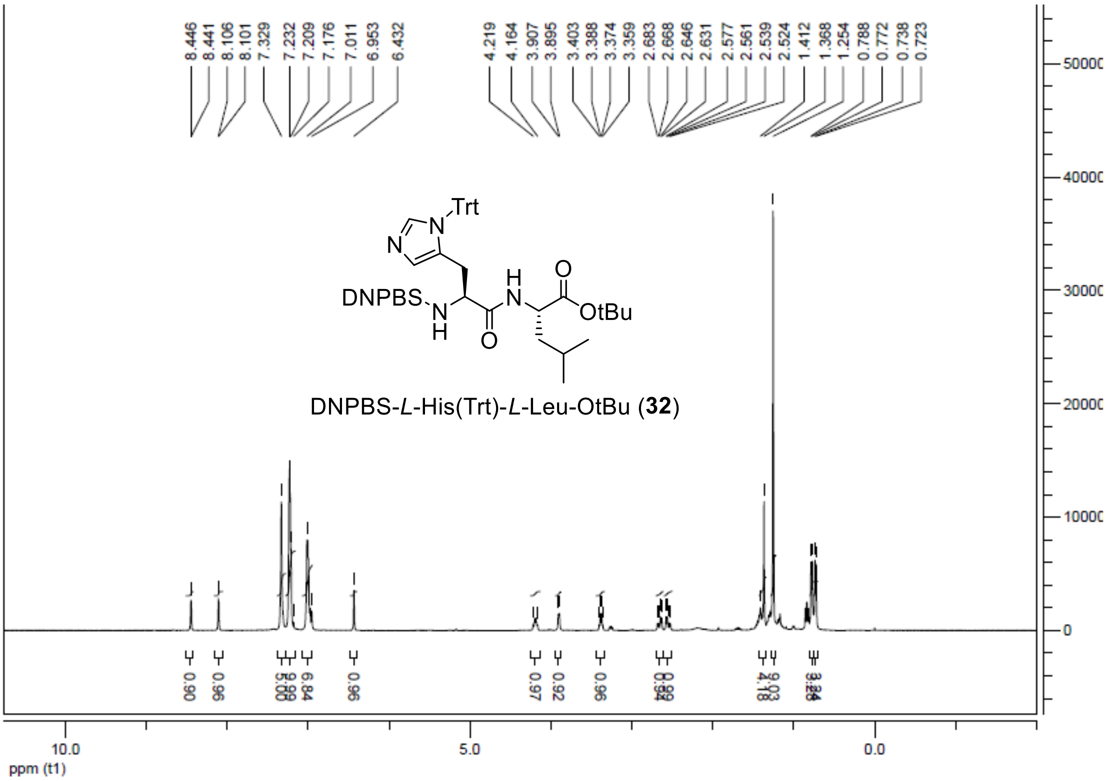


$^{13}C$ NMR spectrum of **32** (in $CDCl_3$):

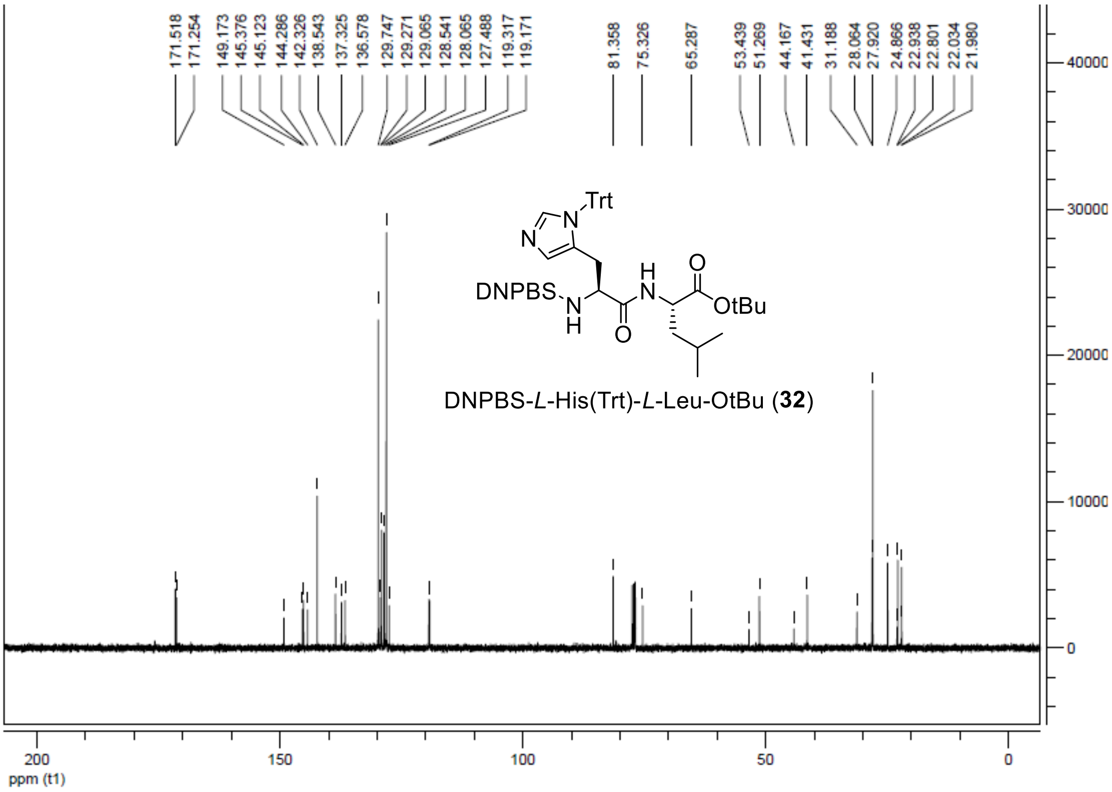

$^{1}H$ NMR spectrum of **34** (in $CDCl_3$):

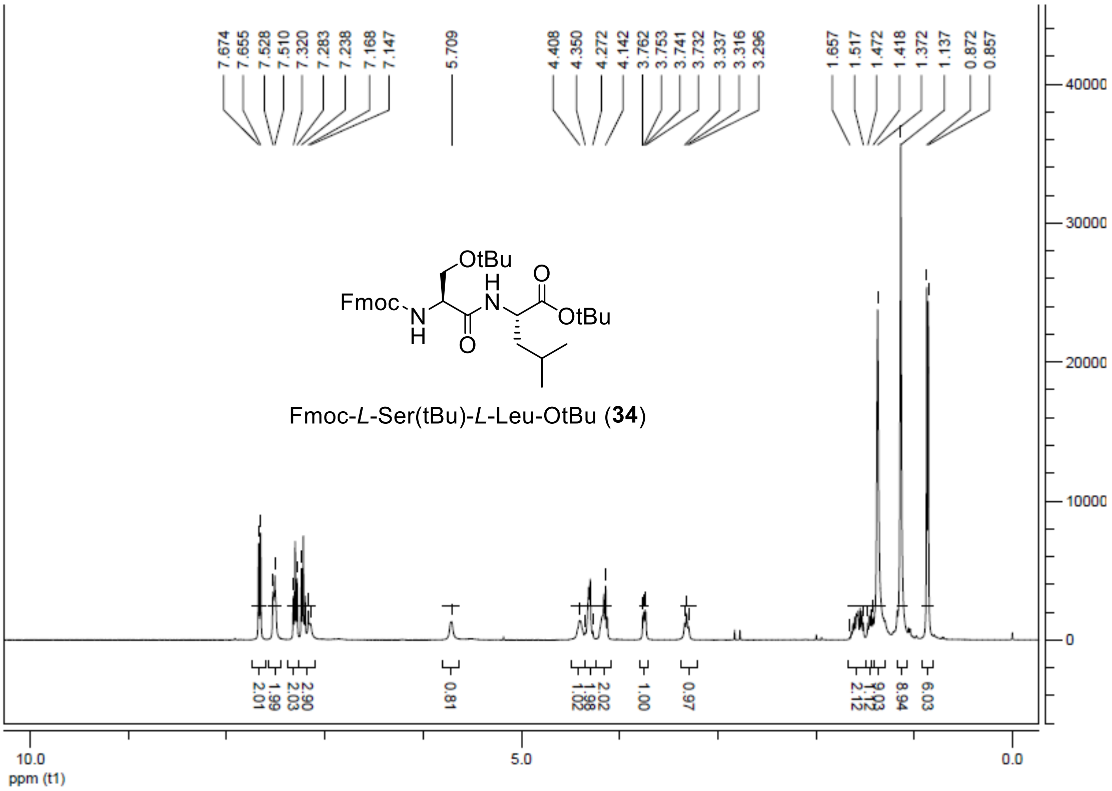


$^{13}C$ NMR spectrum of **34** (in $CDCl_3$):

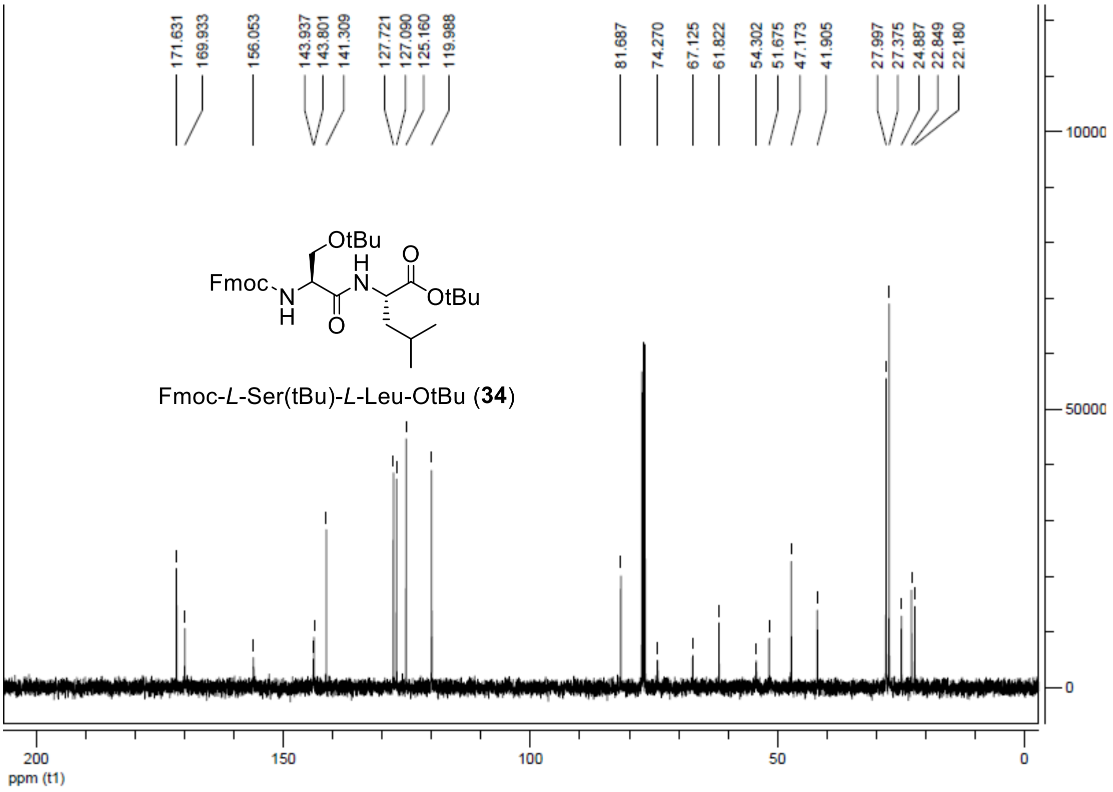

$^{1}$H NMR spectrum of **36** (in $CDCl_3$):

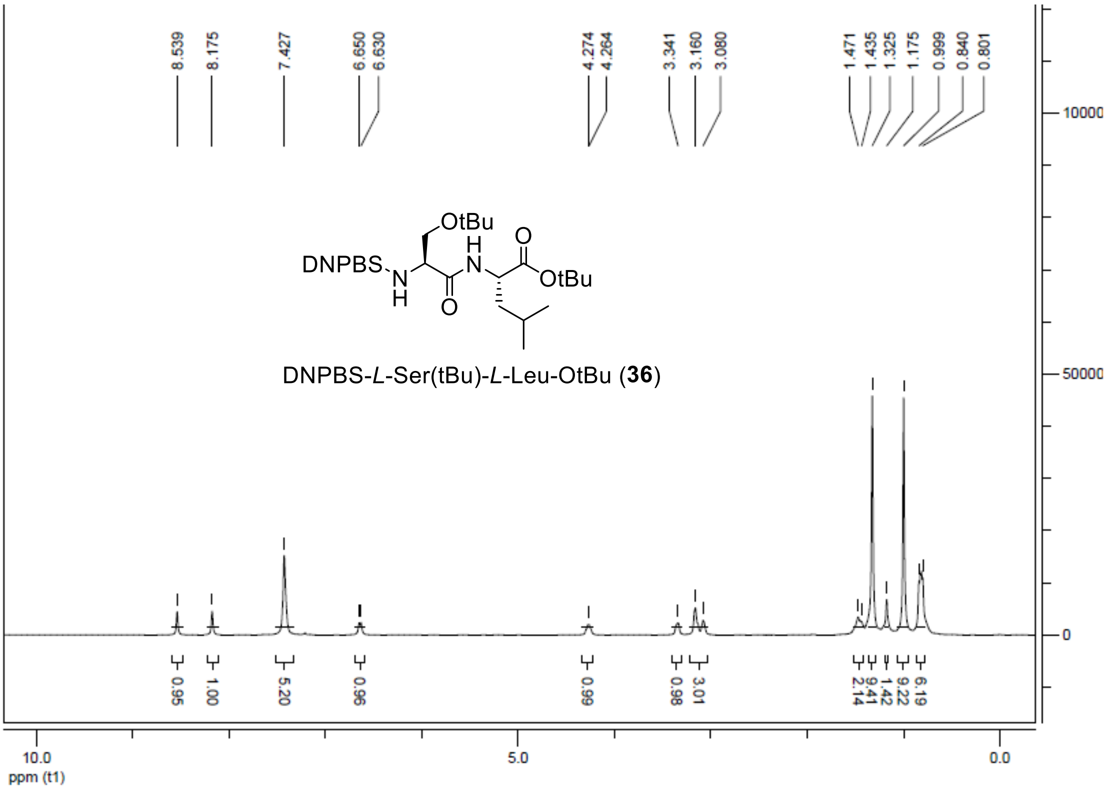


$^{13}$C NMR spectrum of **36** (in $CDCl_3$):

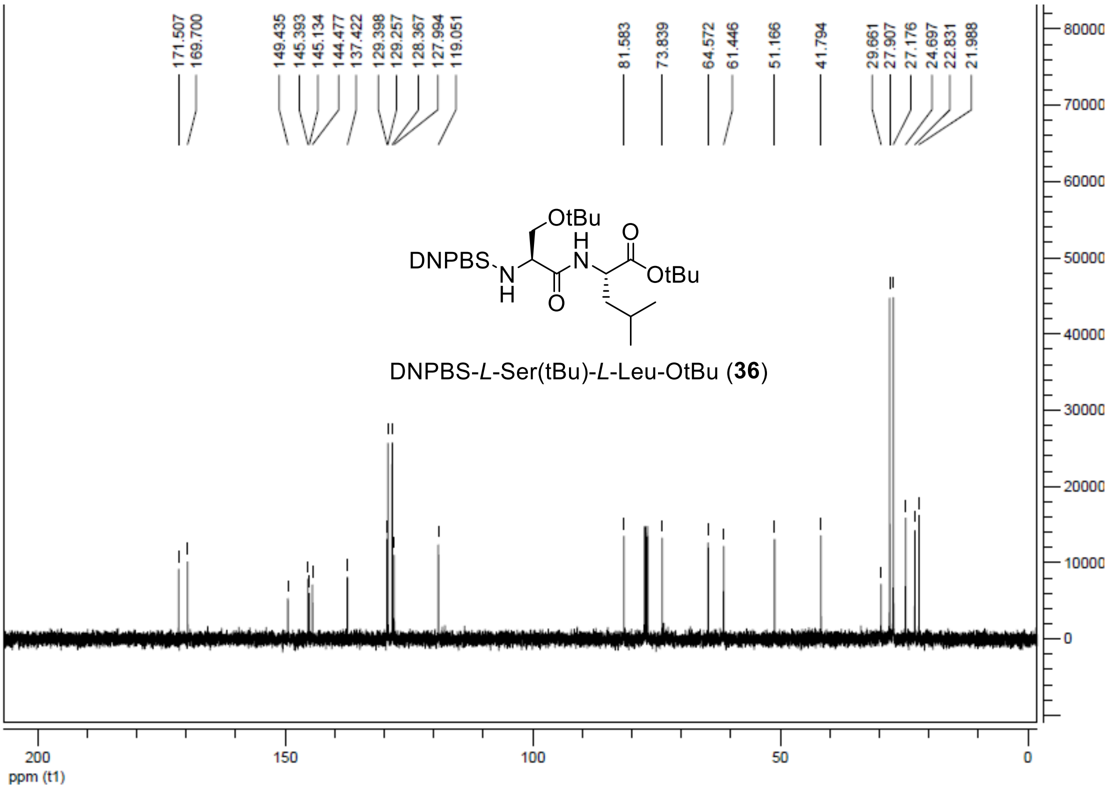

<sup></sup>

$^{1}$H NMR spectrum of **46** (in DMSO):

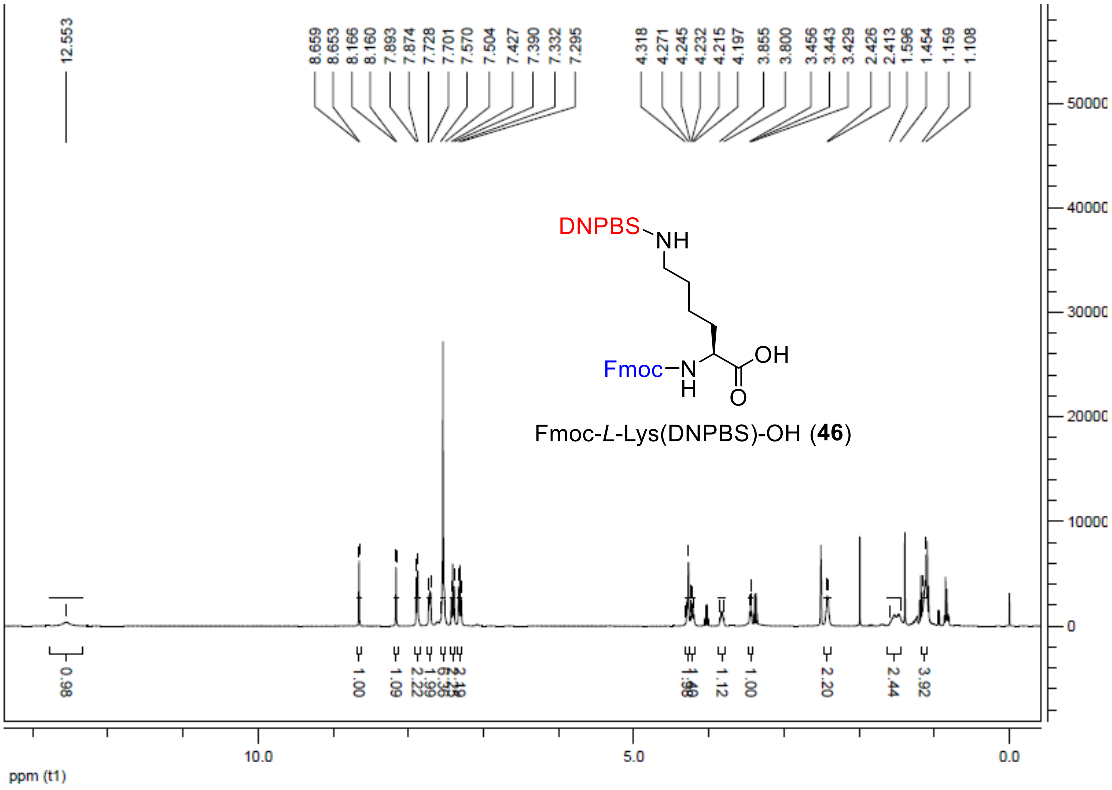


$^{13}$C NMR spectrum of **46** (in DMSO):

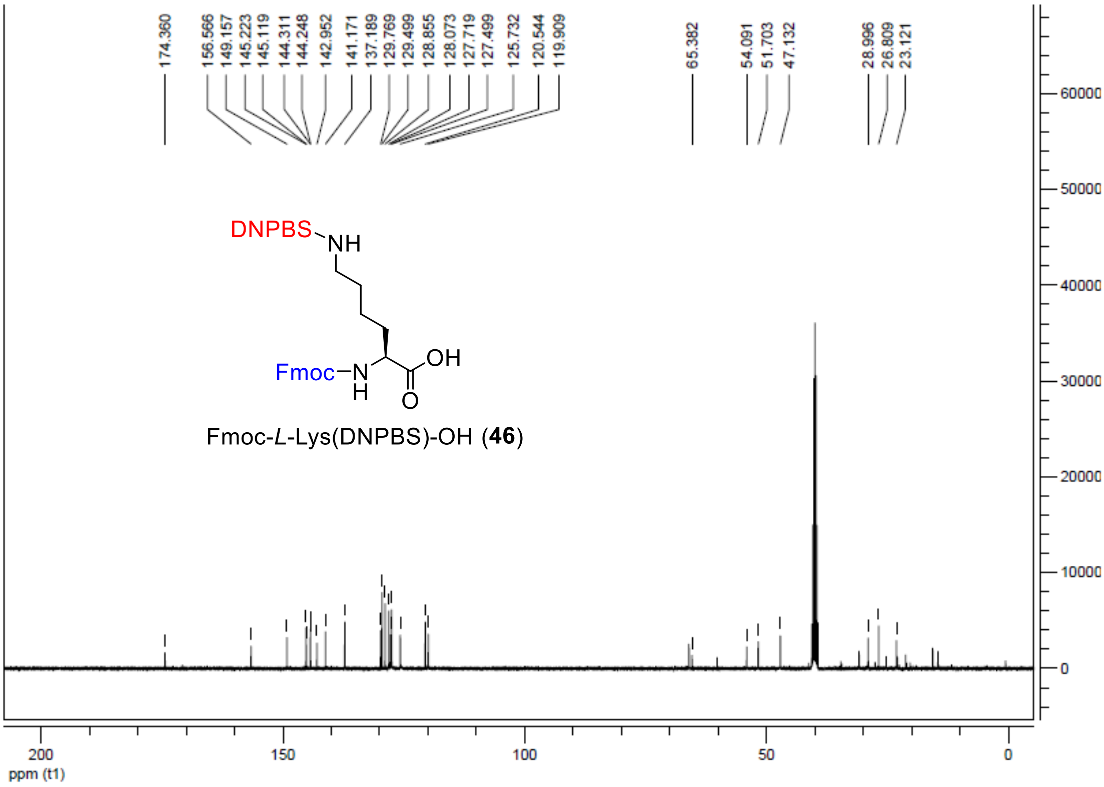

## High-resolution mass spectral (HRMS) of new compounds

## HRMS (ESI-Q-TOF) spectrum of 1:

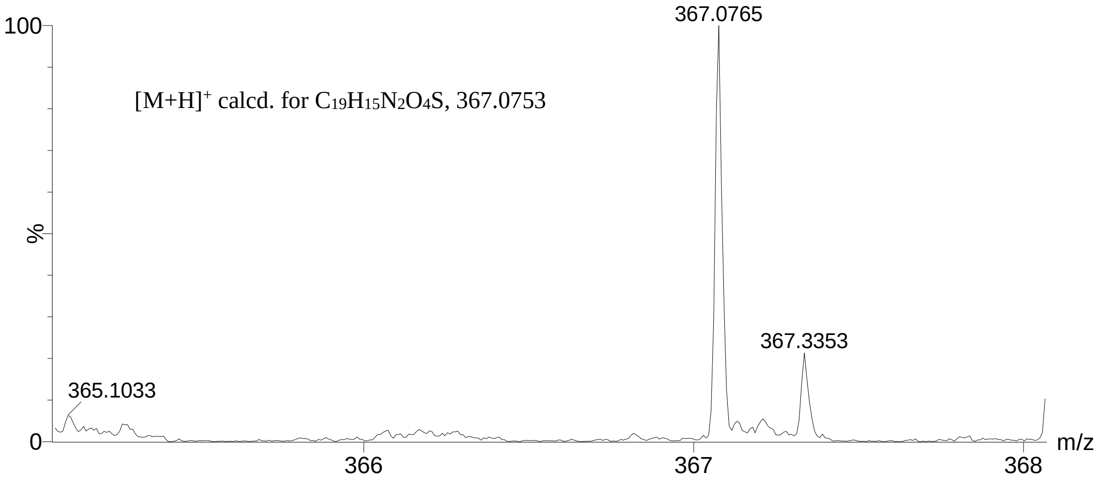


## HRMS (ESI-Q-TOF) spectrum of 3:

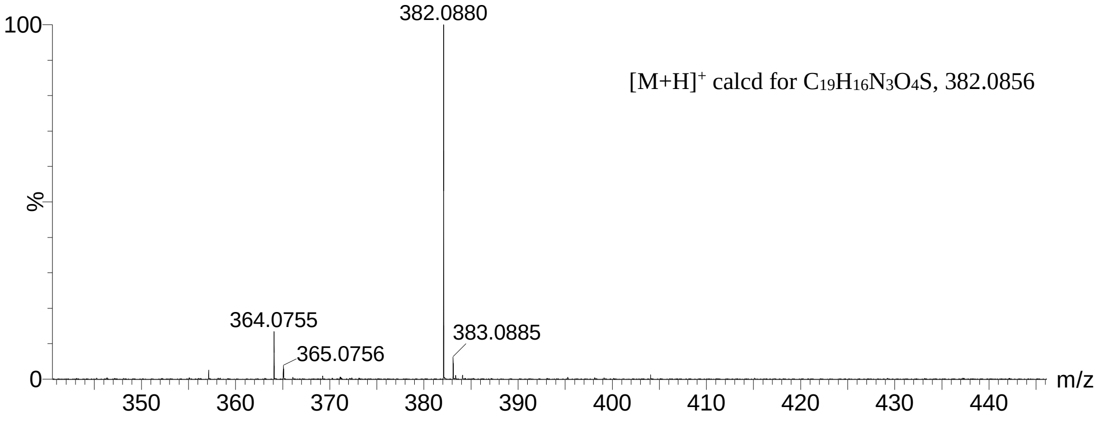


## HRMS (ESI-Q-TOF) spectrum of 4:

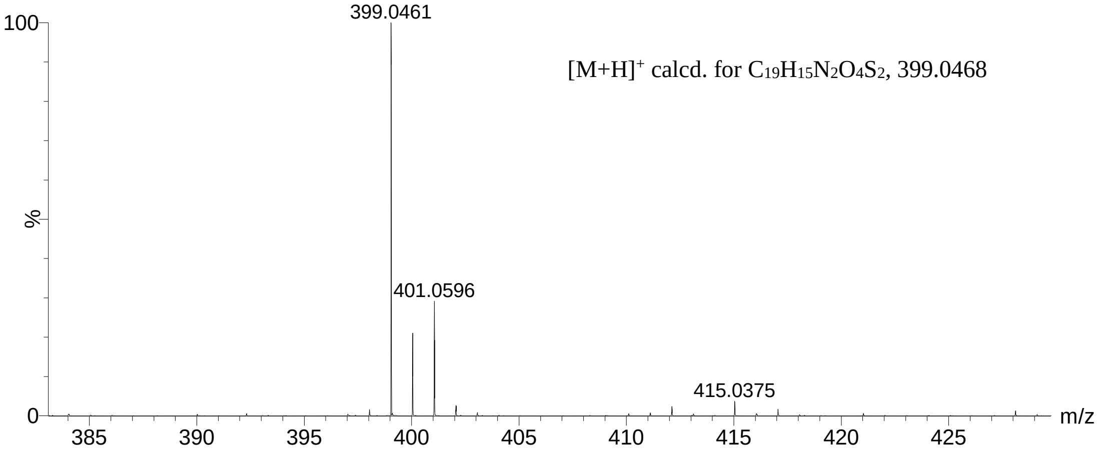

**HRMS (ESI-Q-TOF) spectrum of 5:**

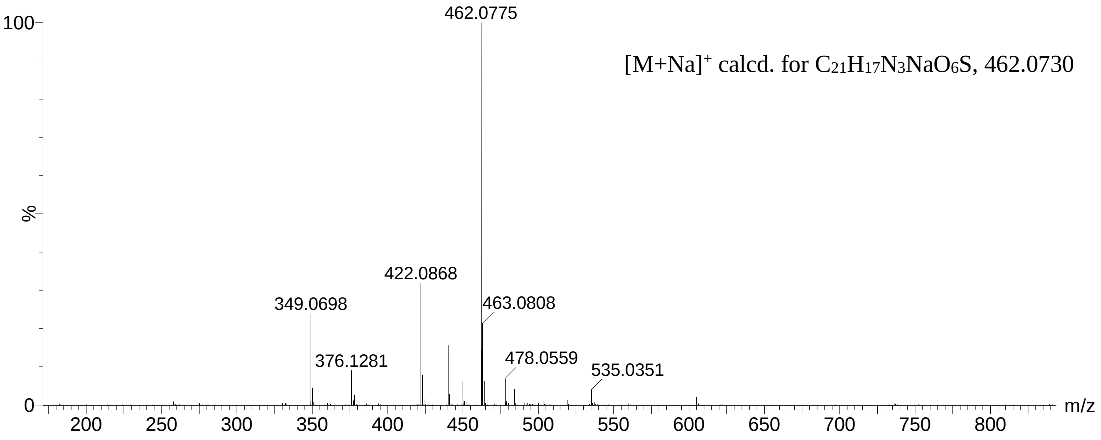


**HRMS (ESI-Q-TOF) spectrum of 6:**

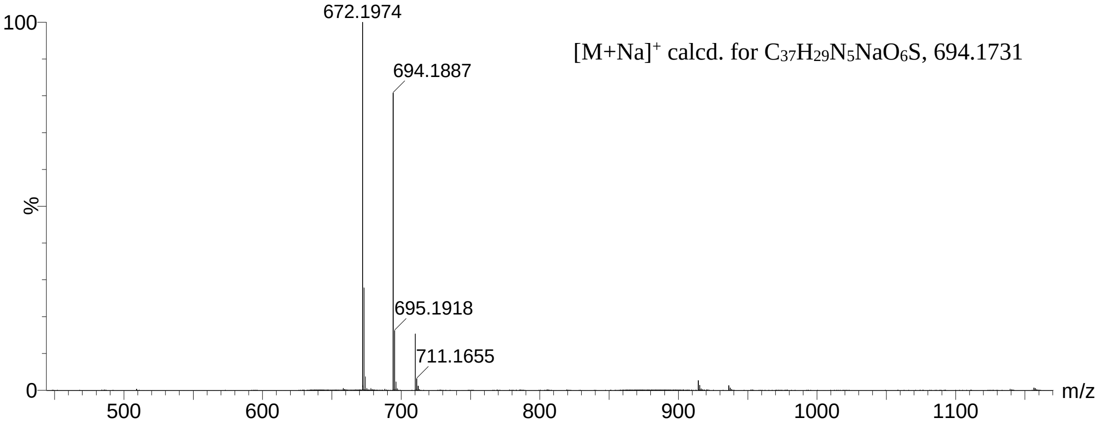


**HRMS (ESI-Q-TOF) spectrum of 7:**

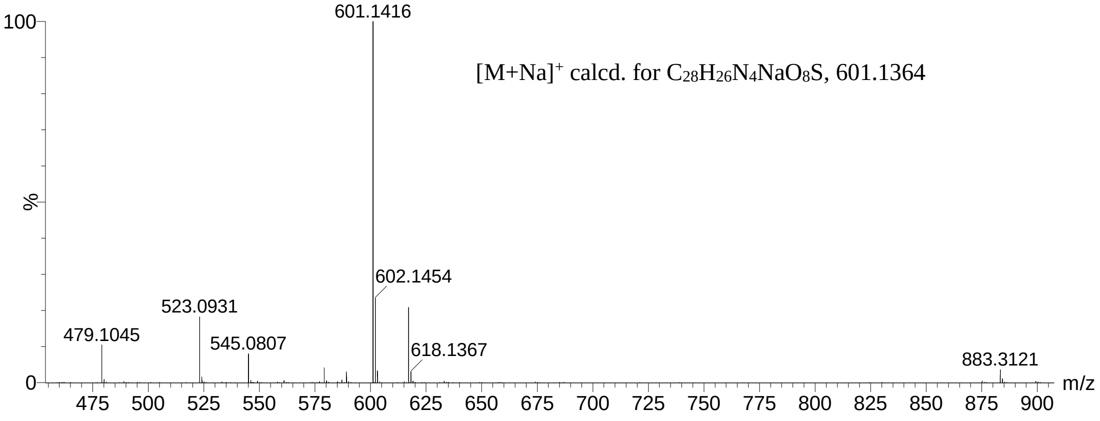

**HRMS (ESI-Q-TOF) spectrum of 8:**

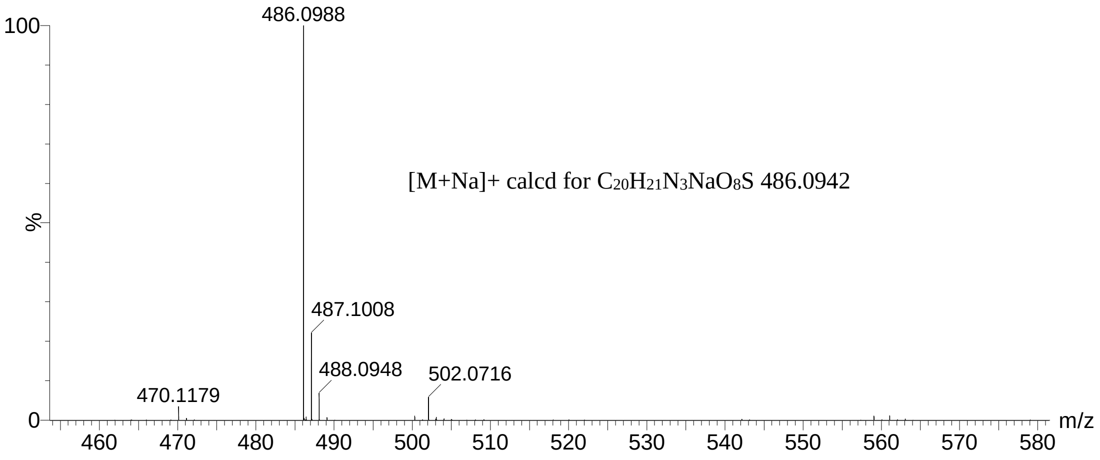


**HRMS (ESI-Q-TOF) spectrum of 9:**

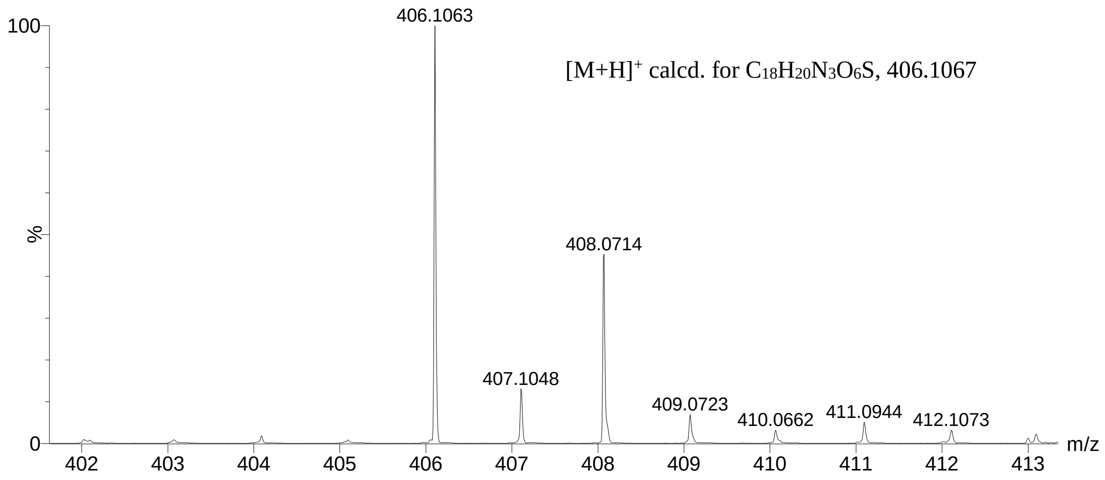


**HRMS (ESI-Q-TOF) spectrum of 10:**

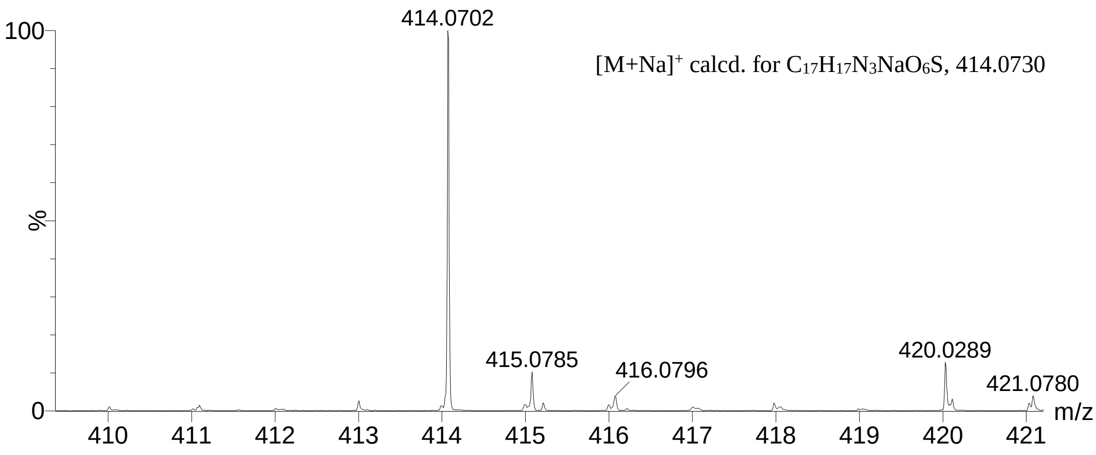

**HRMS (ESI-Q-TOF) spectrum of 11:**

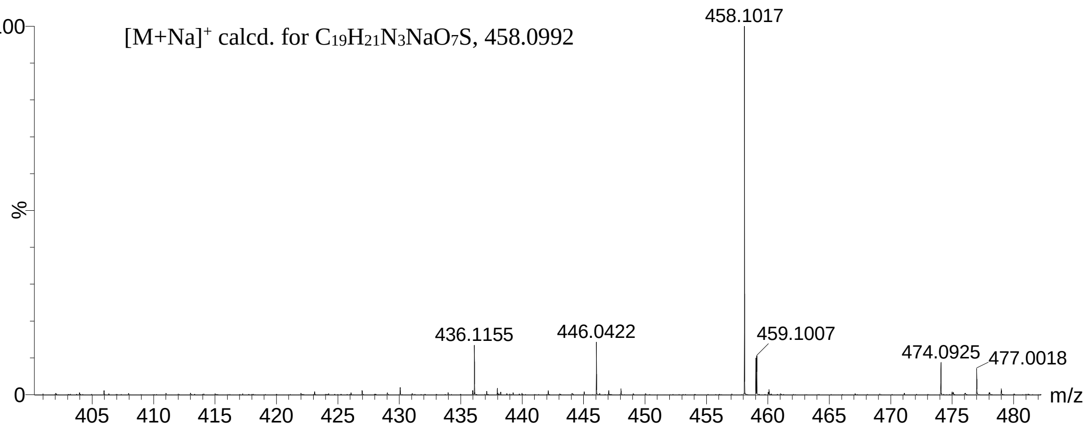


**HRMS (ESI-Q-TOF) spectrum of 12:**

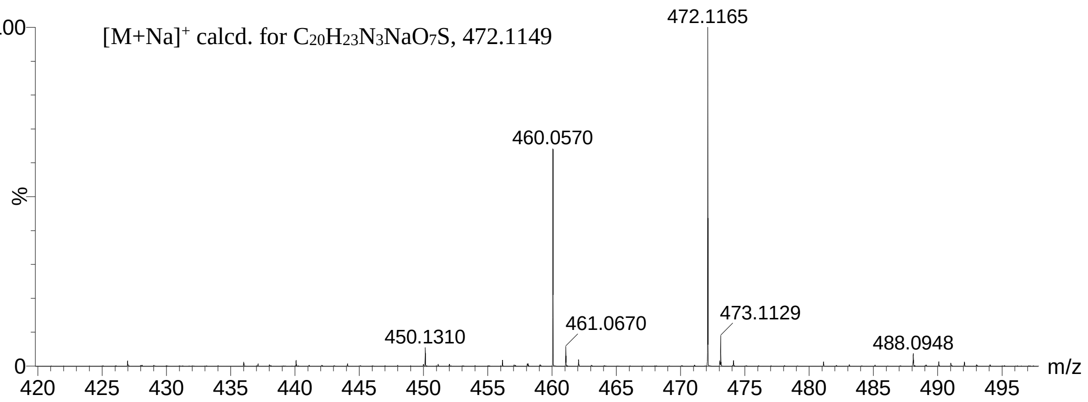


**HRMS (ESI-Q-TOF) spectrum of 13:**

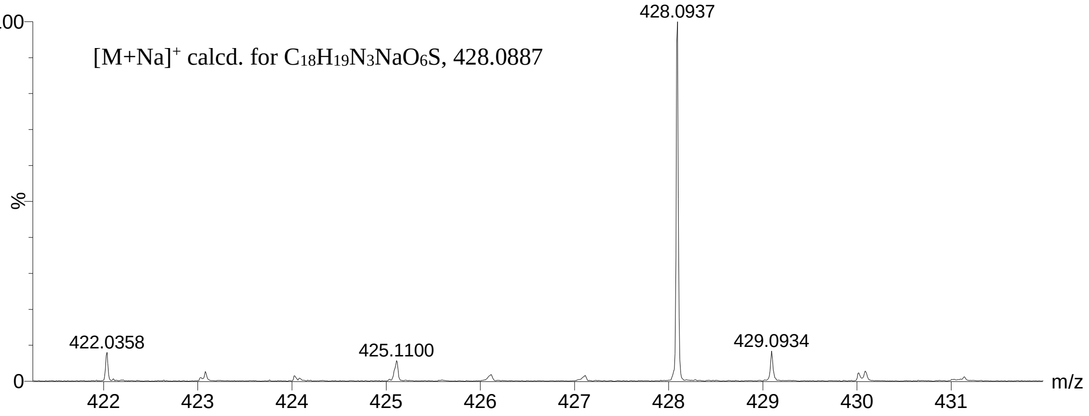

## HRMS (ESI-Q-TOF) spectrum of 14:

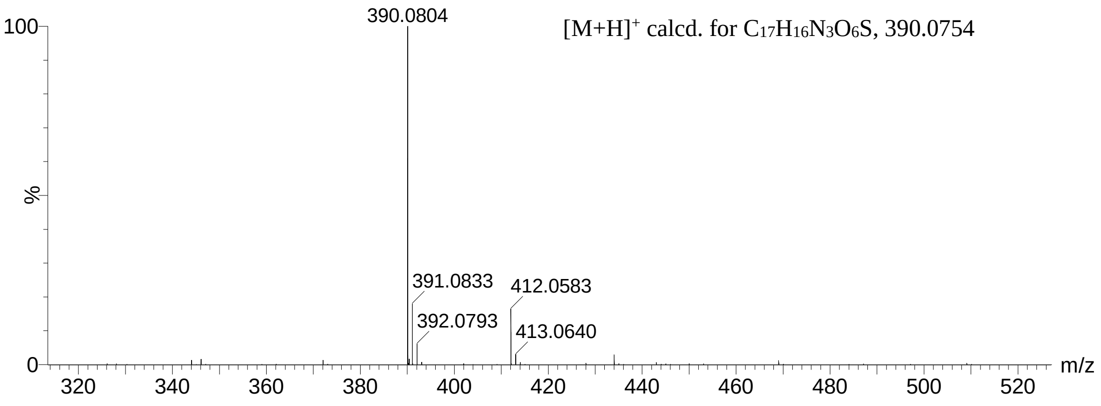


## HRMS (ESI-Q-TOF) spectrum of 15:

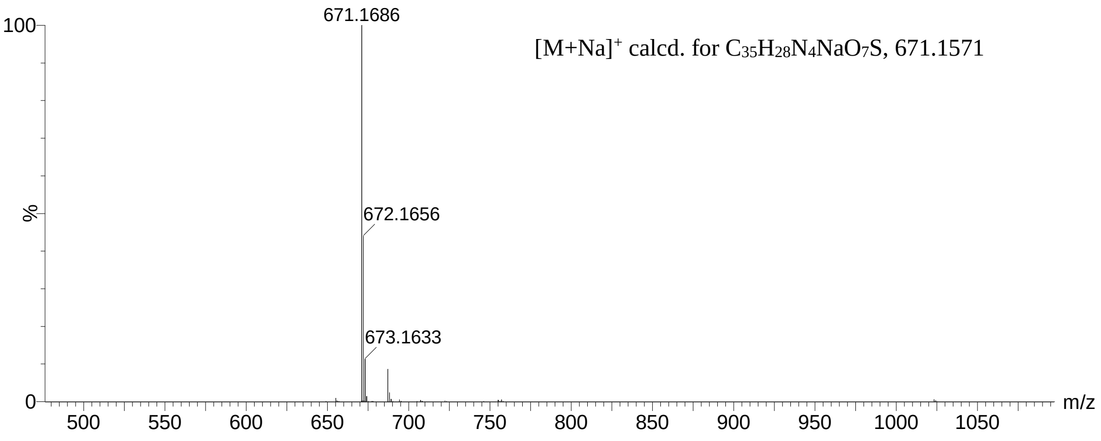


## HRMS (ESI-Q-TOF) spectrum of 16:

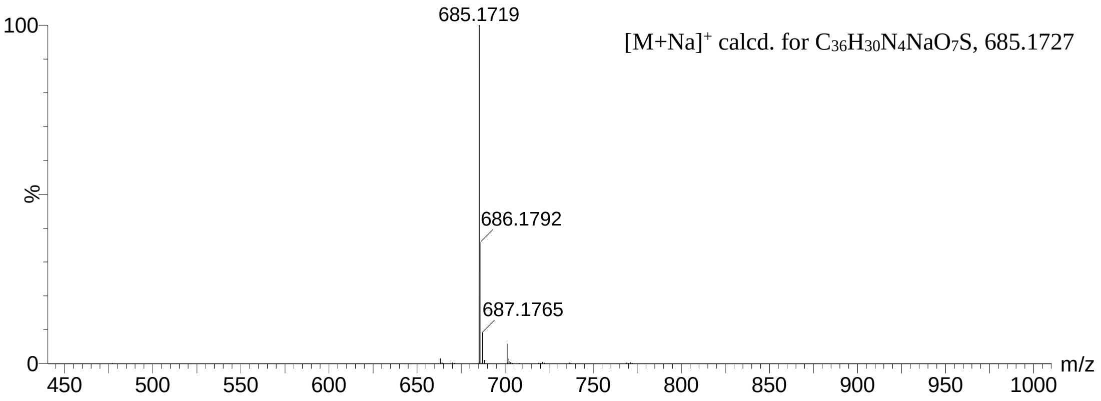

**HRMS (ESI-Q-TOF) spectrum of 17:**

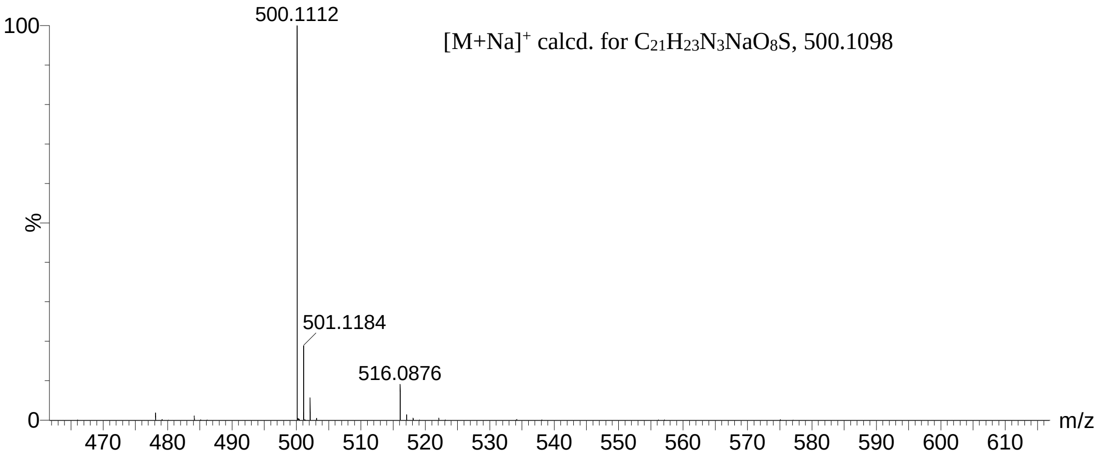


**HRMS (ESI-Q-TOF) spectrum of 18:**

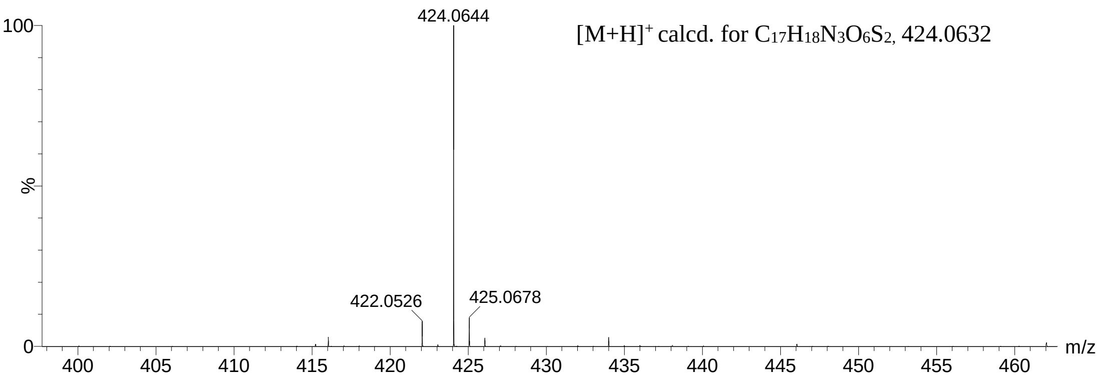


**HRMS (ESI-Q-TOF) spectrum of 19:**

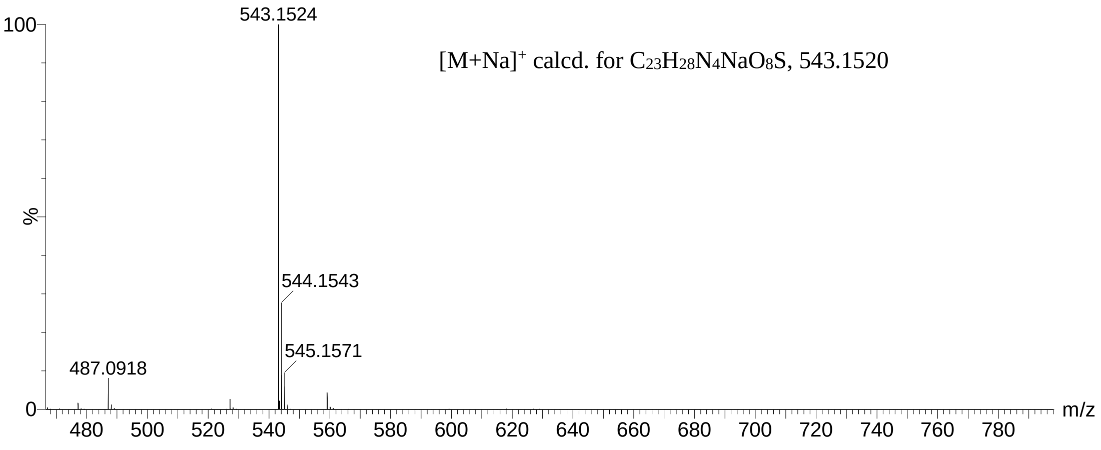

## HRMS (ESI-Q-TOF) spectrum of 20:

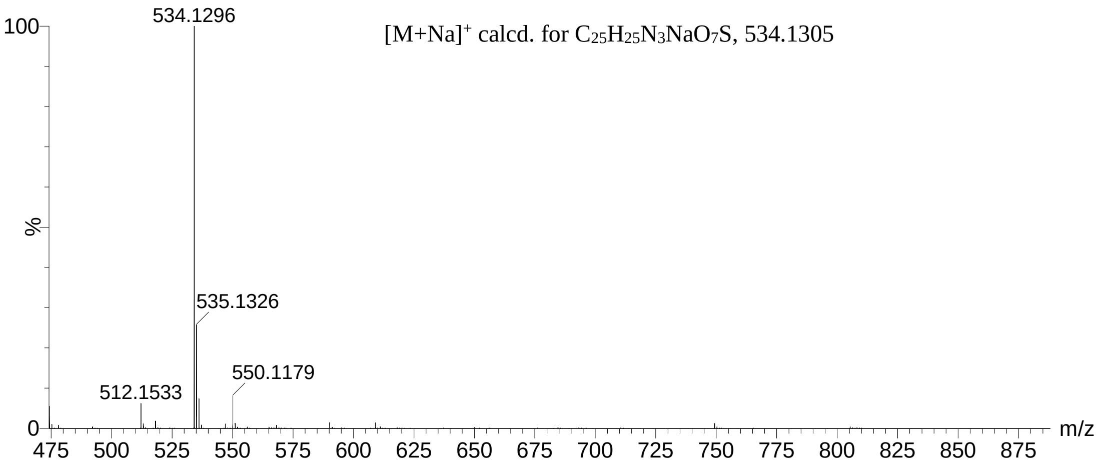


## HRMS (ESI-Q-TOF) spectrum of 21:

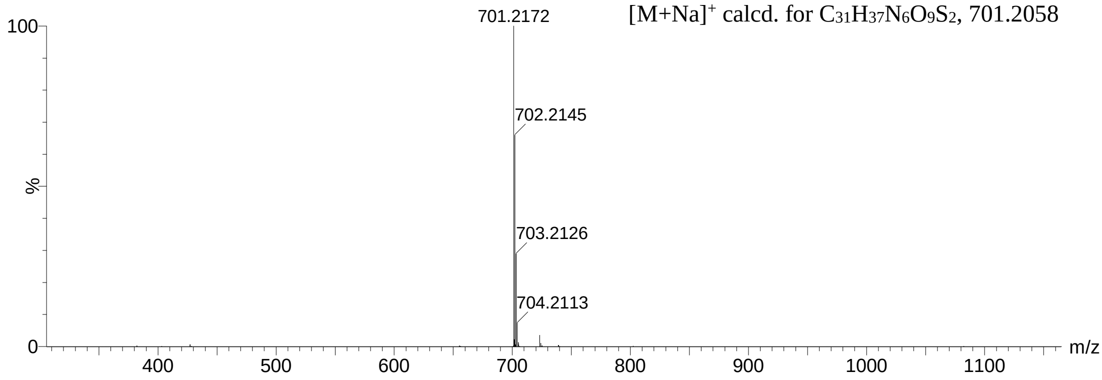


## HRMS (ESI-Q-TOF) spectrum of 22:

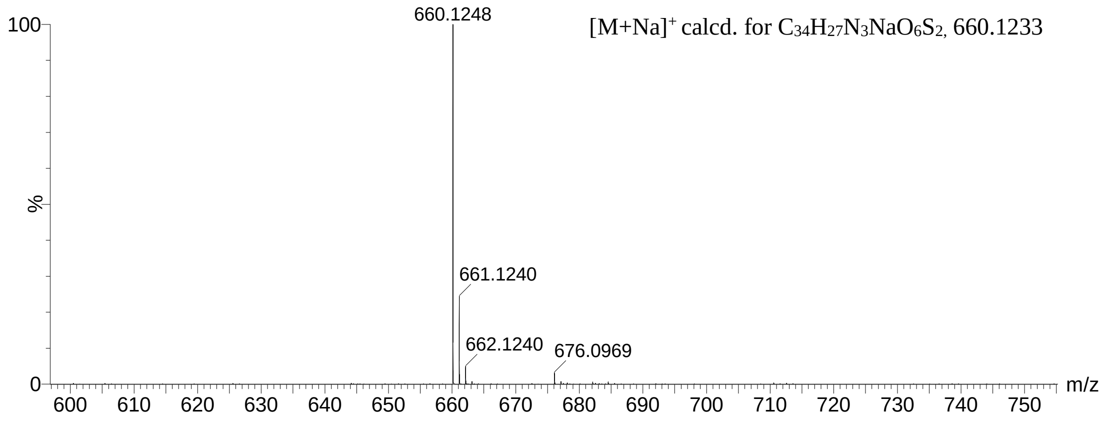

**HRMS (ESI-Q-TOF) spectrum of 23:**

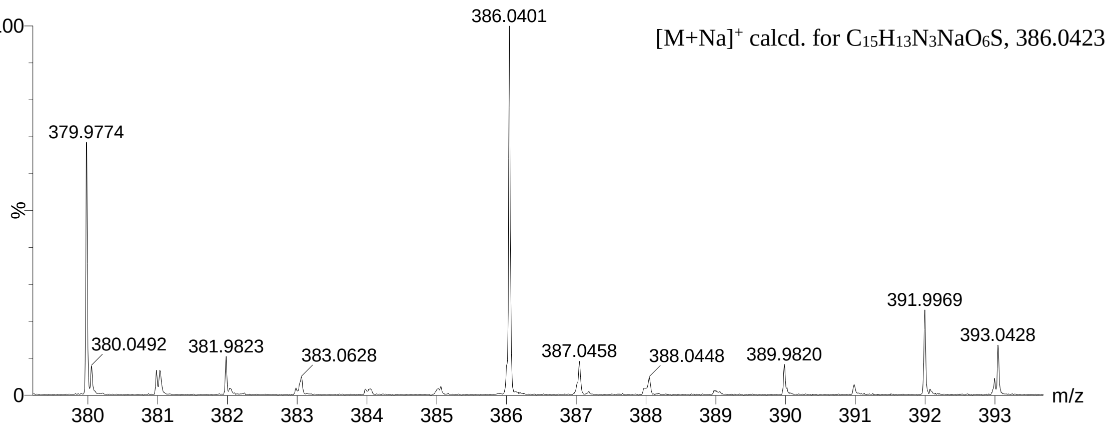


**HRMS (ESI-Q-TOF) spectrum of 24:**

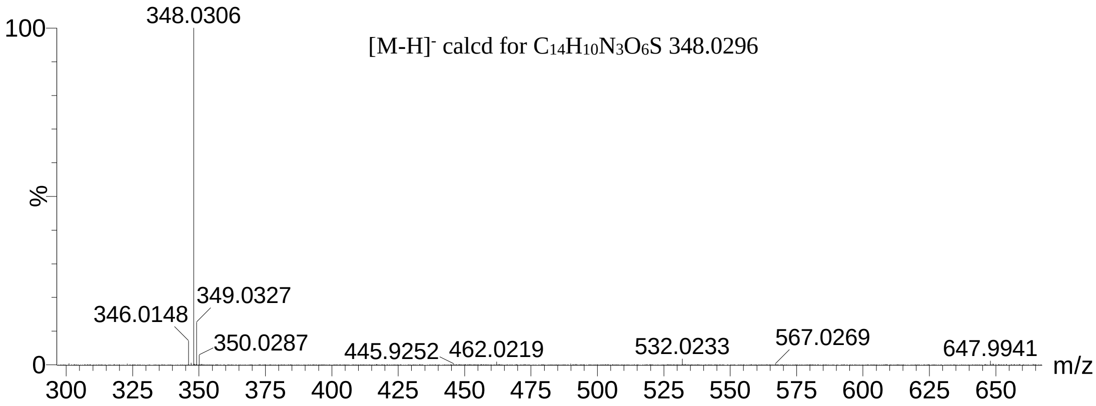


**HRMS (ESI-Q-TOF) spectrum of 26:**

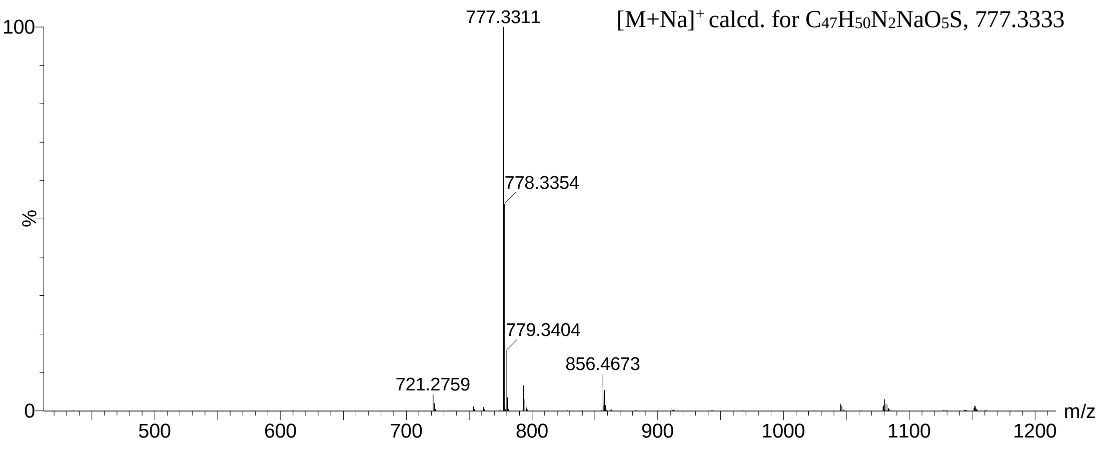

**HRMS (ESI-Q-TOF) spectrum of 28:**

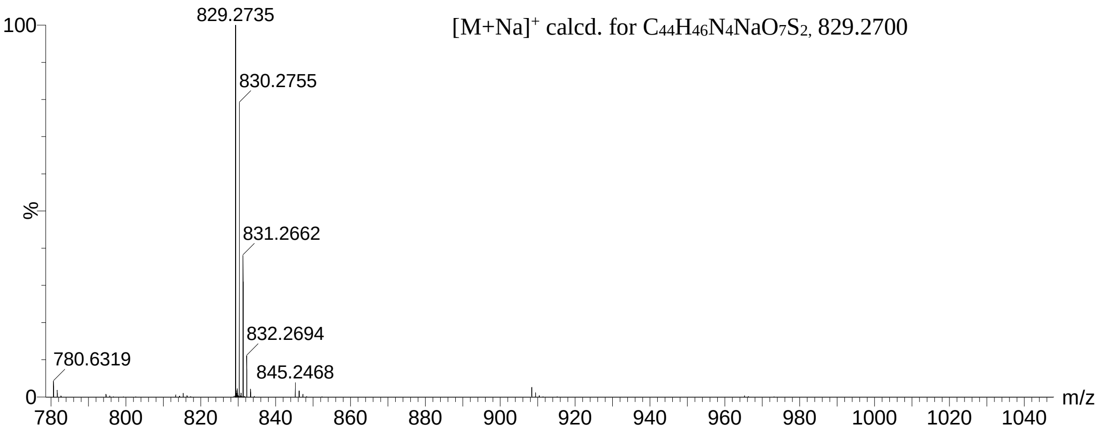


**HRMS (ESI-Q-TOF) spectrum of 30:**

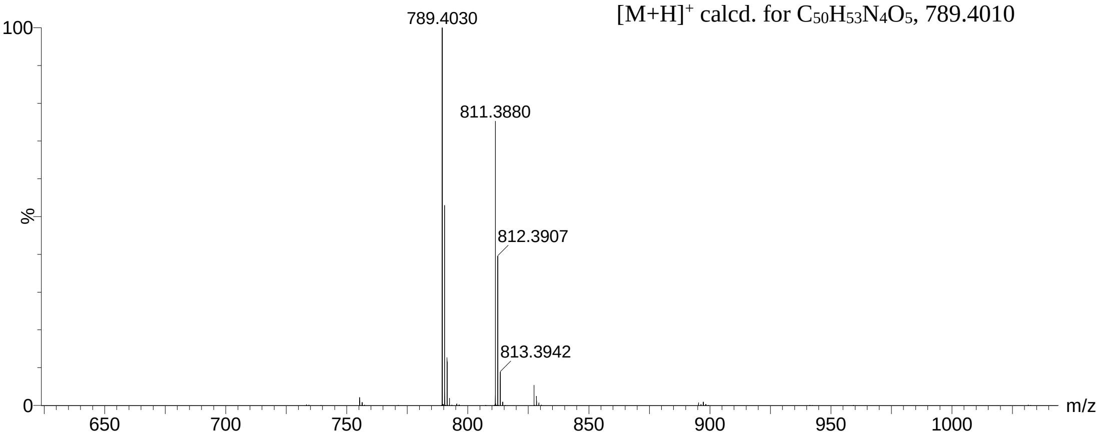


**HRMS (ESI-Q-TOF) spectrum of 32:**

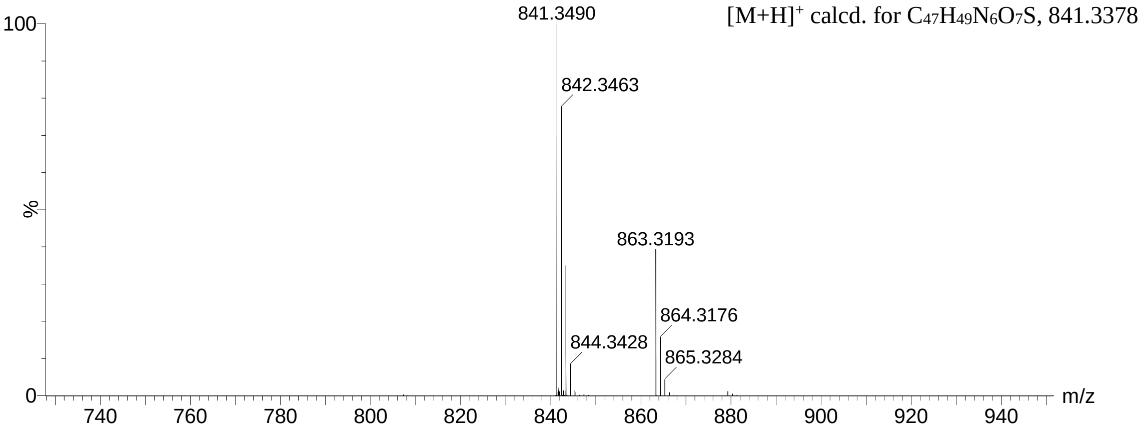

**HRMS (ESI-Q-TOF) spectrum of 34:**

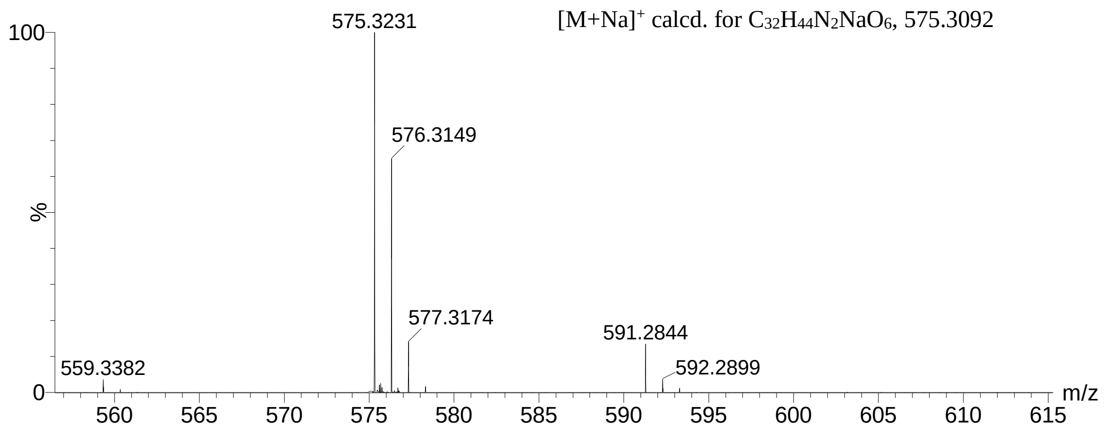


**HRMS (ESI-Q-TOF) spectrum of 36:**

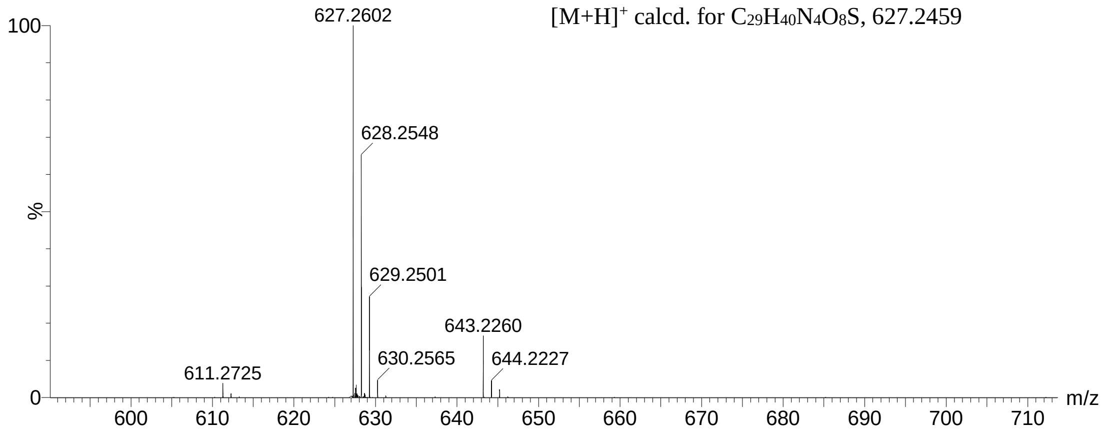


**HRMS (ESI-Q-TOF) spectrum of 38:**

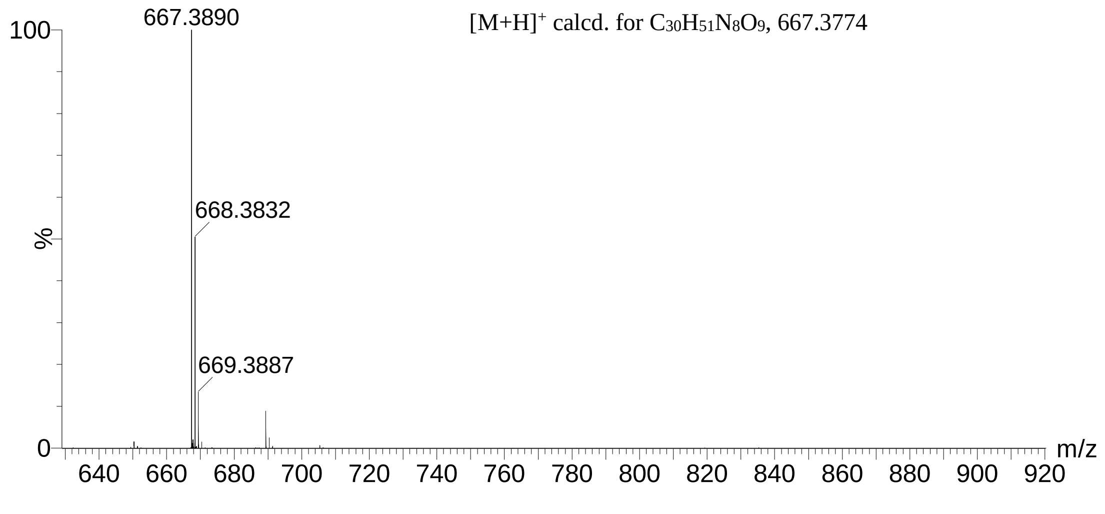

**HRMS (ESI-Q-TOF) spectrum of 39:**

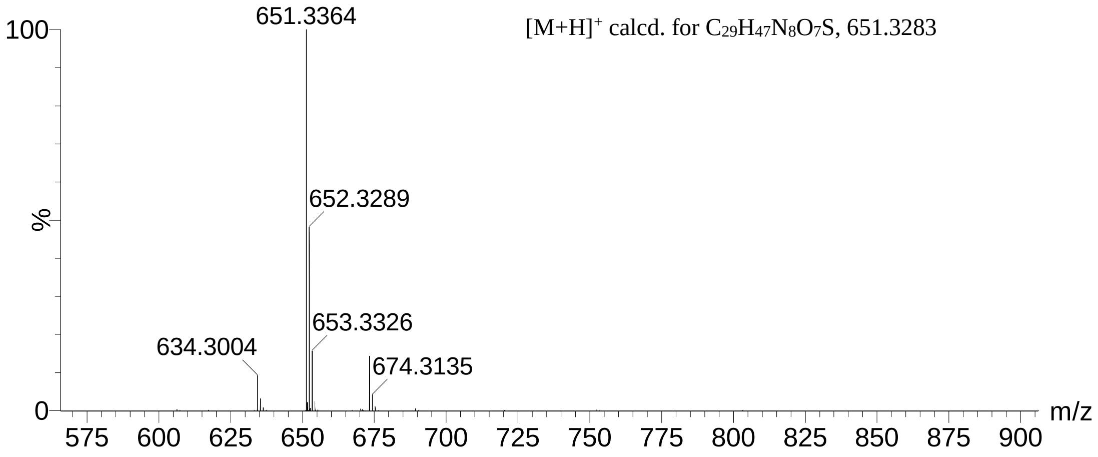


**HRMS (ESI-Q-TOF) spectrum of 40:**

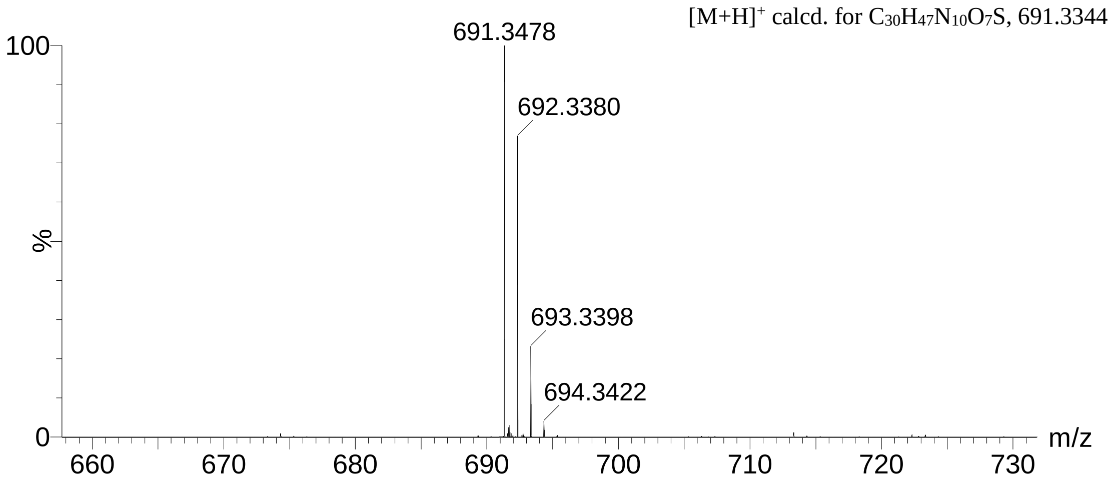


**HRMS (ESI-Q-TOF) spectrum of 41:**

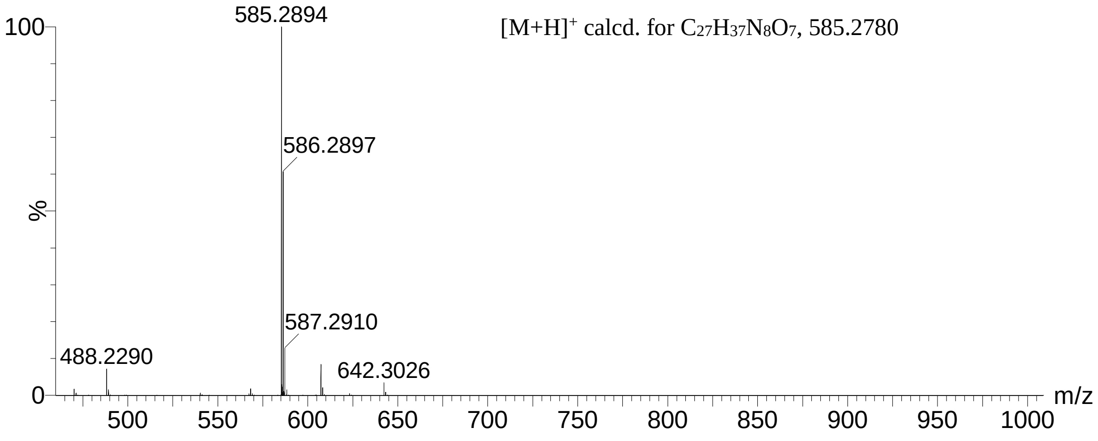

## HRMS (ESI-Q-TOF) spectrum of 42:

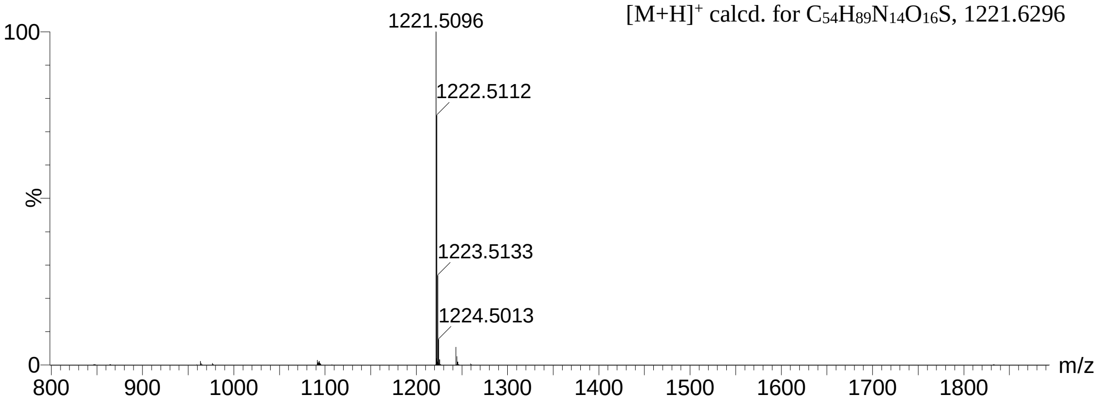


## HRMS (ESI-Q-TOF) spectrum of 43:

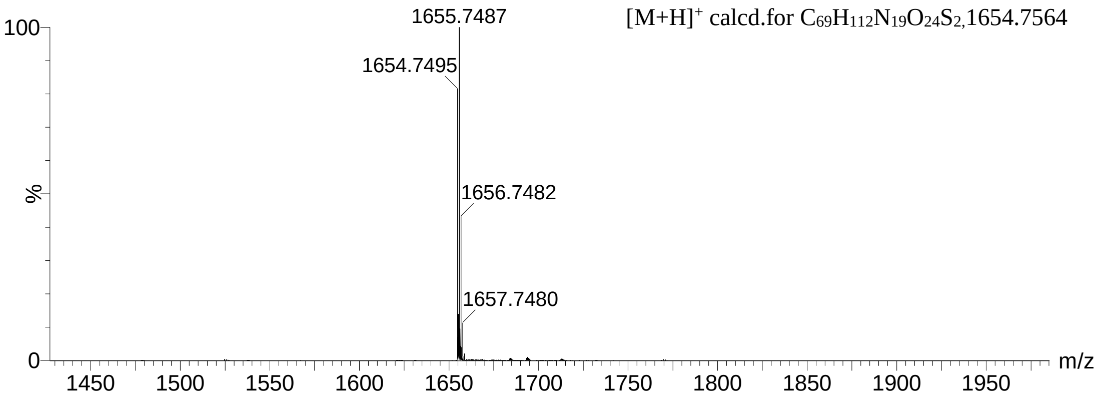


## HRMS (ESI-Q-TOF) spectrum of 44

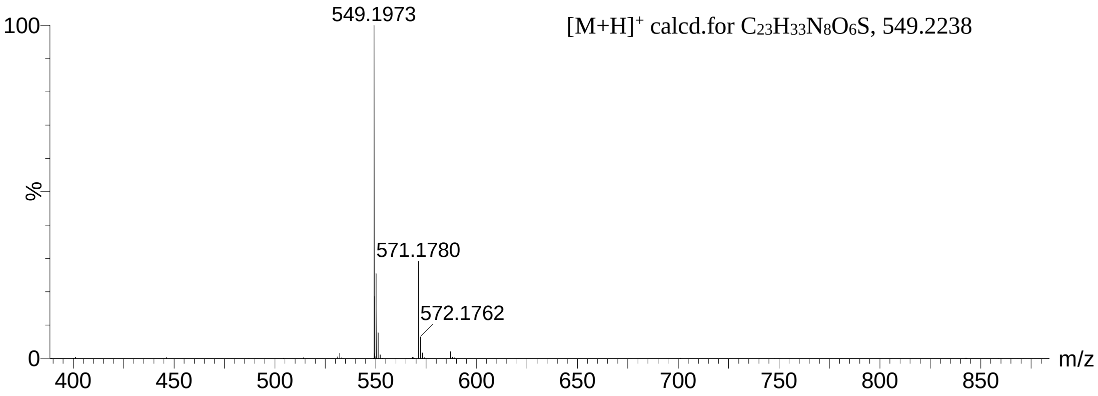

## HRMS (ESI-Q-TOF) spectrum of 45

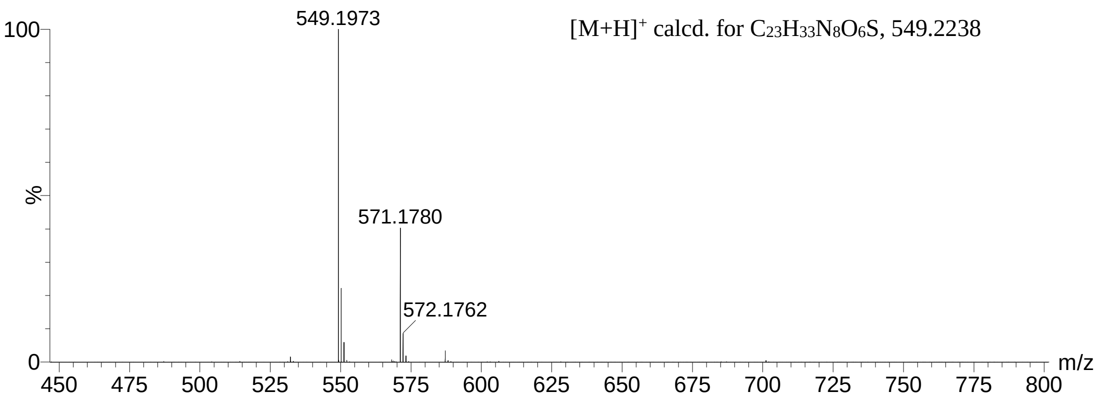


## HRMS (ESI-Q-TOF) spectrum of 46

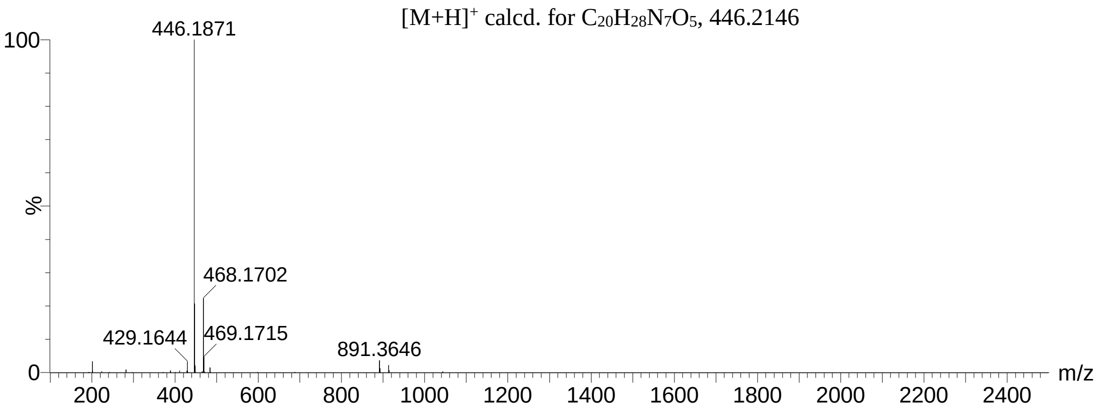


## HRMS (ESI-Q-TOF) spectrum of 47

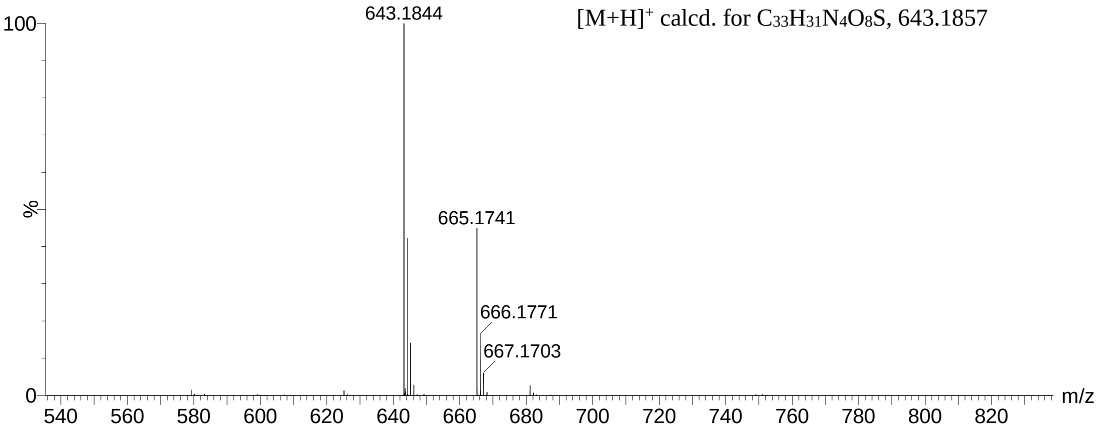

## HRMS (ESI-Q-TOF) spectrum of 51

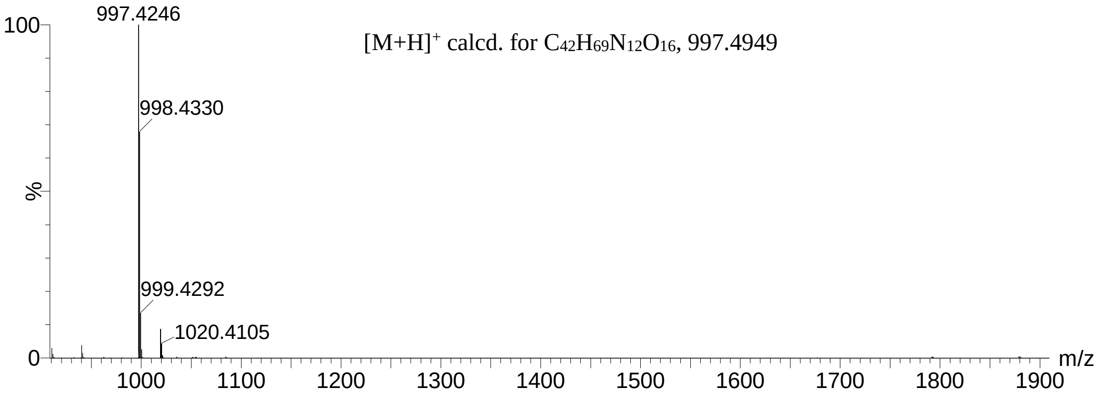


## HRMS (ESI-Q-TOF) spectrum of 52

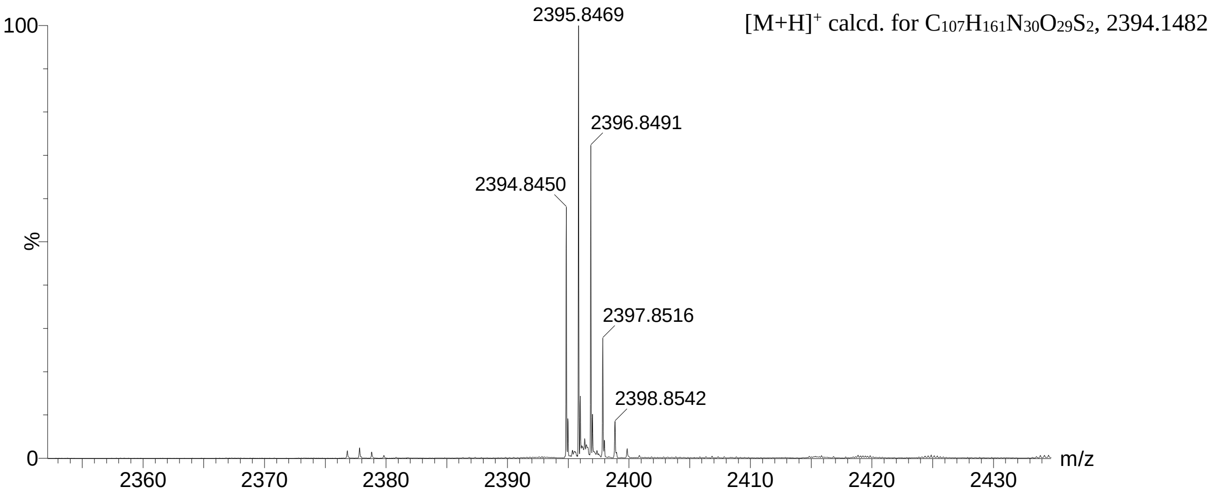

Supplement: Supplementary file 1 — Supplementary Information [file 41467_2023_41115_MOESM1_ESM.pdf]
