# Supplementary material for: Suppression of alpha-carbon racemization in peptide synthesis based on a thiol-labile amino protecting group
Source: Nat Commun. 2023 Sep 1;14:5324. doi: 10.1038/s41467-023-41115-x (PMC10474026; doi:10.1038/s41467-023-41115-x)
Supplement: Supplementary file 2 — Peer review file [file 41467_2023_41115_MOESM2_ESM.pdf]

nature portfolio

Peer Review File

Suppression of Alpha-Carbon Racemization in Peptide Synthesis Based on a Thiol-Labile Amino Protecting Group

Open Access This file is licensed under a Creative Commons Attribution 4.0 International License, which permits use, sharing, adaptation, distribution and reproduction in any medium or format, as long as you give appropriate credit to the original author(s) and the source, provide a link to the Creative Commons license, and indicate if changes were made. In the cases where the authors are anonymous, such as is the case for the reports of anonymous peer reviewers, author attribution should be to 'Anonymous Referee' followed by a clear attribution to the source work. The images or other third party material in this file are included in the article's Creative Commons license, unless indicated otherwise in a credit line to the material. If material is not included in the article's Creative Commons license and your intended use is not permitted by statutory regulation or exceeds the permitted use, you will need to obtain permission directly from the copyright holder. To view a copy of this license, visit http://creativecommons.org/licenses/by/4.0/.

REVIEWER COMMENTS

Reviewer #1 (Remarks to the Author):

The chemistry of solid phase peptide synthesis has continued to evolve since its development by Merrifield more than 60 years ago. Today, the fluorenylmethoxycarbonyl (Fmoc) group remains by far the most popular N-amino protecting group with its removal during synthesis being traditionally mediated by piperidine base cleavage. With the growing demand for more green methods of synthesis, much work is being done on identifying alternatives to not only the base itself, but also the solvent most commonly employed during the synthesis cycles (e.g. dimethlyformamide [DMF]) and, indeed, the Fmoc group. In this generally well-written manuscript, the authors report the development and use of the 2,4-dinitro-6-phenyl-benzene sulfenyl (DNPBS) group as a replacement for the Fmoc group for temporary N-protection of amino acids. Interestingly, the DNPBS group is thiol labile and cleanly removed by treatment with, for example, 1 M p-toluenethiol/pryridine). However, this is not the first thiol-labile protecting group to be reported with the dithiasuccinyl (Dts) group used in SPPS in the 1970s (Barany, G.; Merrifield, R. B. J Am Chem Soc 1977, 99,7363–7365) which the authors need to acknowledge. Detailed studies on the DNPBS group show that this protecting group can be applied to SPPS and that it avoids a number of side reactions, most notably amino acid acylation racemization. This constitutes a welcome addition to the increasing number of interesting recent developments in SPPS including improved reactant stirring, microwave heating, and ultra-fast continuous flow reactions.

Unfortunately, however, DNPBS SPPS clearly requires significant refinement and further other work for it to be a viable alternative to the current, well-established Fmoc-based protocols especially as the latter can be undertaken under controlled conditions to minimize many of the known side reactions. The chemical steps are notably slower to complete, in particular the N(alpha)-deprotection and, further and critically, synthesis size is limited to around 10 amino acid residues or less due to an increasing inability of the solid support to accommodate the alternative synthesis chemistry. It is clear that alternative solvents require examination as a means of keeping the resin sufficiently solvated and swollen, and perhaps alternative N(alpha)-deprotections conditions are required that are faster and more efficient. Despite the clear visual or UV-monitoring advantages afforded by the colour changes following N(alpha)-deprotection of the DNPBS group, these overall limitations necessitate that this work undergo further improvement before publication can be considered. For now, a specialist journal such as Organic Letters is a better forum for these preliminary results.

1. Are the BNPBS-amino acids soluble in water or aqueous solvent mixtures?
2. It is not clear from the text if resin shrinkage (p. 12) or excess swelling (p. 18) is an issue with DNPBS SPPS. I am assuming the former.

Typos/edits:
p. 3, line 2: 'R. Bruce ...', not 'Bruce R. ...'
p. 10, line 2: 'Rink', not 'rink'.

Reviewer #2 (Remarks to the Author):

Key results
The authors introduced a new N-terminal protecting group (2,4-dinitro-6-phenyl-benzene sulfenyl group) that is linked via a S–N bond. Removal is performed via 1 M p-toluenethiol/pyridine und mild basic conditions. During their work, the authors introduced the new protecting group to the 20 canonical amino acids and made coupling experiments to determine the racemization of His, Ser and Cys followed by some solid phase peptide synthesis of model peptides with a comparison of the classical Fmoc-SPPS side reactions (racemization, aspartimide formation). In addition, they showed

orthogonality with the Fmoc-group that allows to use the DNPBS group for a branched synthesis or targeted side chain modifications.

Validity
The authors introduced an interesting new protecting group for amine functions in peptide synthesis that merits publication. Its orthogonality to the Fmoc group allows for specific side chain modifications during peptide synthesis. The authors claim that the new strategy is also of value in terms of sustainability (green chemistry) It can be doubted that this is indeed the case.The option to replace DMF with THF or DCM is interesting, but in terms of greener solvents definitely no improvement. Based on the fundamental ETH Zurich publication Capello C, Fischer U, Hungerbühler K (2007) Green Chem 9:927–934) the two tiered assessment of environmental, health and safety (EHS) and energy demand (which can be regarded as a quick LCA type calculation) for THF is even worse than DMF. The authors were very honest and pointed out the limitations of the protection group itself. The limitation to 10 amino acids peptide length is a major limitation for peptide synthesis. I am not sure if the limitation is caused by the used resin (resins with PEG Linkers or PEG based are commercially available to test this possibility), as the problem occurs after the growing peptide chains reaches a certain peptide I would advise to check if it is caused by limited solubility of the peptide chain in THF.

Significance
The publication offers a new route for peptide synthesis that has in some aspects advantages over current synthesis strategies. The paper therefore merits publication. The claim that it contributes to green chemistry as already pointed out above does not hold and should be corrected and the method should also be discussed with reference to current literature on sustainable peptide synthesis. There are many actual publications that point out the current problems in this field (e.g. ACS Green Chemistry Institute Pharmaceutical Roundtable publication J. Org. Chem. 2019, 84, 8, 4615–4628). Many actual publications in this field regarding the use of sustainable solvents (organic solvents or water based) have not been considered (e.g. water based: Angew. Chem. Int. Ed. 2020, 59, 12984; Chem. Pharm. Bull. 2004, 52, 422-427; Tetrahed. Lett. 2004, 45, 9293; J. Pawlas, J. H. Rasmussen, ChemSusChem 2021, 14, 3231; Jad et al. used 2-MeTHF γ-Valero-lactone N-formylmorpholine (NFM); Lawrenson et al. used propylene carbonate (PC)).
The limitation of the peptide length to 10 amino acids is a major limitation for this method. There might be an application in LPPS as the authors' stated. The usage of the protecting group is via an orthogonal strategy with Fmoc is interesting regarding the modification of Lys side chains. The reduced racemisation of His using the protecting group and deprotection mechanism could be useful, but it needs to be shown whether it would work if His is coupled with the DNPBS group during an Fmoc synthesis or if it fails after 10 amino acids peptide length.

Data and methodology
The authors have attached all synthesis routes, the specific NMR data and further analytical data. In view of the work, all necessary data is shown.

Suggested improvements
The authors should validate the potential influence of the used resin on the peptide length be switching to a resin for more polar solvents with PEG linkers like Tentagel resins or totally PEG based resin like ChemMatrix. Testing different greener solvents is highly advised if the point of sustainability should still be included in the publication: Switching the solvents would also be an option to check whether the limitation of 10 amino acids is related to the solvent (would be possible with DMF as well).

Clarity and context
The test is well readable and presented in an understandable way. The analytical data in the paper and the supplement underline the work of the authors and most of the conclusions. Some additional work in the field of green solvents, sustainable chemistry with focus on peptide synthesis should be included (see above and references).

References
Actual publications in the field of (sustainable) peptide synthesis regarding alternative organic solvents or water approaches based have not been considered (e.g. water based: Chem. Pharm. Bull. 2004, 52, 422-427; Tetrahed. Lett. 2004, 45, 9293; Angew. Chem. Int. Ed. 2020, 59, 12984; Organic: ChemSusChem 2021, 14, 3231; Jad et al. used 2-MeTHF γ-Valero-lactone N-formylmorpholine (NFM); Lawrenson et al. used propylene carbonate (PC)) and some should be added if the if the claim to sustainability in the paper should be maintained.

Reviewer #3 (Remarks to the Author):

This work presents a new amino group protection/deprotection strategy for peptide synthesis that yields peptides in which alpha-carbon racemization and aspartimide formation is suppressed efficiently. The latter two reactions are typical side reactions found in peptide synthesis using the broadly applied Fmoc or Boc protection group strategies. The side products are usually hard to remove from the desired product due to the high similarity (i.e. retention time in RP-HPLC), and a solution to this problem would thus be of great benefit. The authors produced all natural amino acids with the DNPBS protecting group and applied them for peptides synthesis. They nicely demonstrate that side products could be suppressed efficiently, which was most evident with amino acids such as His and Cys where racemization happens more frequently. The authors show that the method is suited for solid phase peptide synthesis, though not for longer peptides due to problems with protection group stability or too early removal of the protection group. These latter problems are not so well characterized as described in the major point 1 below. Another attractive application of the new protecting group is the generation of branched peptides, as demonstrated by the authors.

The work and results are appealing to me overall for the two main reasons: i) the work addresses a central problem (racemization of alpha carbons and aspartimide formation) and ii) the strategy is new and different than established amino-protecting chemistries. A major limitation is that it appears to work not as robust as Fmoc or Boc chemistry, as seen with side products and the problems with longer peptides. I recommend to characterize the exact problems in more detail (see major point 1 below). To benefit from the method already, one option may be to make longer peptides using Fmoc chemistry and to use DNPBS building blocks for amino acids that are prone to racemization (His, Cys). I recommend to test such a "hybrid" option, in order to complement the work with an appealing application for a broad audience (see major point 2 below).

Major:
1. Problems of new protecting group and side products:
The new strategy for amino group protection/deprotection appears to be not suited for longer peptides, as transparently stated/concluded by the authors. Some data points to the chemical basis of the problems (e.g. figure 3a), by providing examples of side products, but all this is not much detailed. I recommend that the authors perform a detailed side-by-side comparison of the new "DNPBS" method and Fmoc synthesis in terms of coupling efficiency on solid phase. E.g. they could do a similar comparison as in Figure 2b, for different amino acids (but testing the coupling on solid phase, as this is relevant).

2. Problems with longer peptides:
The new approach is obviously not suited for longer peptides (as transparently indicated by the authors), which will limit much the application of the DNPBS protecting group. The authors indicate that it may be used selectively for some amino acids where razemization on the alpha carbon is particularly a problem (e.g. Fmoc-His, Fmoc Cys), wherein other couplings are done with Fmoc amino acids. I find this an interesting idea and recommend to assess if this works. E.g. the authors could apply the hybrid method to 2-3 longer peptides (e.g. well-known biologically active peptide) that

suffered from racemization in Fmoc synthesis, and they could assess if the new "DNPBS" method offers an advantage if applied in combination with Fmoc chemistry.

Minor:

- Discussion: the authors repeat the advantages of the new method by indicating "4 merits", starting with the ability to monitor the deprotection due to absorbance, followed by suppression of alpha-carbon racemization etc. I recommend to list the merits in the order of importance (i.e. starting with the racemization suppression).

-Title: the authors used the term "cleaner" which I think is not ideal as the coupling efficiency is less good than in Fmoc synthesis (and the method thus actually less clean). I recommend to indicate the true advantages (e.g. racemization-free …)

Reviewer #1 (Remarks to the Author):

The chemistry of solid phase peptide synthesis has continued to evolve since its development by Merrifield more than 60 years ago. Today, the fluorenylmethoxycarbonyl (Fmoc) group remains by far the most popular N-amino protecting group with its removal during synthesis being traditionally mediated by piperidine base cleavage. With the growing demand for more green methods of synthesis, much work is being done on identifying alternatives to not only the base itself, but also the solvent most commonly employed during the synthesis cycles (e.g. dimethlyformamide [DMF]) and, indeed, the Fmoc group. In this generally well-written manuscript, the authors report the development and use of the 2,4-dinitro-6-phenyl-benzene sulfenyl (DNPBS) group as a replacement for the Fmoc group for temporary N-protection of amino acids. Interestingly, the DNPBS group is thiol labile and cleanly removed by treatment with, for example, 1 M p-toluenethiol/pryridine). However, this is not the first thiol-labile protecting group to be reported with the dithiasuccinyl (Dts) group used in SPPS in the 1970s (Barany, G.; Merrifield, R. B. J Am Chem Soc 1977, 99,7363–7365) which the authors need to acknowledge. Detailed studies on the DNPBS group show that this protecting group can be applied to SPPS and that it avoids a number of side reactions, most notably amino acid acylation racemization. This constitutes a welcome addition to the increasing number of interesting recent developments in SPPS including improved reactant stirring, microwave heating, and ultra-fast continuous flow reactions.

Response: We thank the reviewer for pointing out the reference omission. We have now included this reference in the revised manuscript. Please see Ref. 14.

Unfortunately, however, DNPBS SPPS clearly requires significant refinement and further other work for it to be a viable alternative to the current, well-established Fmoc-based protocols especially as the latter can be undertaken under controlled conditions to minimize many of the known side reactions. The chemical steps are notably slower to complete, in particular the N(alpha)-deprotection and, further and critically, synthesis size is limited to around 10 amino acid residues or less due to an increasing inability of the solid support to accommodate the alternative synthesis chemistry. It is clear that alternative solvents require examination as a means of keeping the resin sufficiently solvated and swollen, and perhaps alternative N(alpha)-deprotections conditions are required that are faster and more efficient. Despite the clear visual or UV-monitoring advantages afforded by the colour changes following N(alpha)-deprotection of the DNPBS group, these overall limitations necessitate that this work undergo further improvement before publication can be considered. For now, a specialist journal such as Organic Letters is a better forum for these preliminary results.

Response: We have now refined the DNPBS SPPS. We tried alternative solvents and resins and found that DNPBS SPPS can be used for synthesis of longer peptides based on ChemMatrix resin. Although the coupling efficiency of DNPBS SPPS is not comparable to the that of standard Fmoc SPPS especially for long peptides, the orthogonality of the DNPBS SPPS and Fmoc SPPS allows these two methodologies to be combined to synthesize peptides that would be difficult to prepare by either method

alone.

1. Are the DNPBS-amino acids soluble in water or aqueous solvent mixtures?

Response: The DNPBS-amino acids are insoluble in water or aqueous mixtures. We have pointed this out in the revised manuscript. Please see Page 6, the last sentence.

2. It is not clear from the text if resin shrinkage (p. 12) or excess swelling (p. 18) is an issue with DNPBS SPPS. I am assuming the former.

Response: We observed the resin shrinkage as the peptide chain length increased. We have now deleted the sentence "In addition, the lower efficiency of DNPBS SPPS for long oligopeptide synthesis was due mainly to severe swelling of the resin" in the revised manuscript.

Typos/edits:
p. 3, line 2: 'R. Bruce ...', not 'Bruce R. ...'
Response: "Bruce R. Merrifield" has been changed to "R. Bruce Merrifield".

p. 10, line 2: 'Rink', not 'rink'.
Response: "rink amide" has been changed to "Rink amide" through the whole manuscript.

Reviewer #2 (Remarks to the Author):

Key results
The authors introduced a new N-terminal protecting group (2,4-dinitro-6-phenyl-benzene sulfenyl group) that is linked via a S–N bond. Removal is performed via 1 M p-toluenethiol/pyridine und mild basic conditions. During their work, the authors introduced the new protecting group to the 20 canonical amino acids and made coupling experiments to determine the racemization of His, Ser and Cys followed by some solid phase peptide synthesis of model peptides with a comparison of the classical Fmoc-SPPS side reactions (racemization, aspartimide formation). In addition, they showed orthogonality with the Fmoc-group that allows to use the DNPBS group for a branched synthesis or targeted side chain modifications.
Response: Thank you for the overall very positive comments about this manuscript.

Validity
The authors introduced an interesting new protecting group for amine functions in peptide synthesis that merits publication. Its orthogonality to the Fmoc group allows for specific side chain modifications during peptide synthesis. The authors claim that the new strategy is also of value in terms of sustainability (green chemistry) It can be doubted that this is indeed the case. The option to replace DMF with THF or DCM is interesting, but in terms of greener solvents definitely no improvement. Based on the fundamental ETH Zurich publication Capello C, Fischer U, Hungerbühler K (2007)

Green Chem 9:927–934) the two tiered assessment of environmental, health and safety (EHS) and energy demand (which can be regarded as a quick LCA type calculation) for THF is even worse than DMF. The authors were very honest and pointed out the limitations of the protection group itself. The limitation to 10 amino acids peptide length is a major limitation for peptide synthesis. I am not sure if the limitation is caused by the used resin (resins with PEG Linkers or PEG based are commercially available to test this possibility), as the problem occurs after the growing peptide chains reaches a certain peptide I would advise to check if it is caused by limited solubility of the peptide chain in THF.
Response: We thank the reviewer for the very good suggestion. We have tried alternative solvents and resins and found that DNPBS SPPS can be used for synthesis of peptides longer than 10 residues based on ChemMatrix resin.

Significance
The publication offers a new route for peptide synthesis that has in some aspects advantages over current synthesis strategies. The paper therefore merits publication. The claim that it contributes to green chemistry as already pointed out above does not hold and should be corrected and the method should also be discussed with reference to current literature on sustainable peptide synthesis. There are many actual publications that point out the current problems in this field (e.g. ACS Green Chemistry Institute Pharmaceutical Roundtable publication J. Org. Chem. 2019, 84, 8, 4615–4628). Many actual publications in this field regarding the use of sustainable solvents (organic solvents or water based) have not been considered (e.g. water based: Angew. Chem. Int. Ed. 2020, 59, 12984; Chem. Pharm. Bull. 2004, 52, 422-427; Tetrahed. Lett. 2004, 45, 9293; J. Pawlas, J. H. Rasmussen, ChemSusChem 2021, 14, 3231; Jad et al. used 2-MeTHF γ-Valero-lactone N-formylmorpholine (NFM); Lawrenson et al. used propylene carbonate (PC)).
Response: We are grateful to the reviewer for pointing out our misunderstanding regarding green solvents. We have now included the relevant references in the revised manuscript. Please see Ref. 18-23.

According to the reviewer's suggestion, we have carried out addition experiments and demonstrated that DNPBS SPPS was compatible with greener solvent such as γ-Valero-lactone and *N*-formylmorpholine. We have now included the results in the revised manuscript. Please see Page 12-13, and Figure 3e.

The limitation of the peptide length to 10 amino acids is a major limitation for this method. There might be an application in LPPS as the authors' stated. The usage of the protecting group is via an orthogonal strategy with Fmoc is interesting regarding the modification of Lys side chains. The reduced racemisation of His using the protecting group and deprotection mechanism could be useful, but it needs to be shown whether it would work if His is coupled with the DNPBS group during an Fmoc synthesis or if it fails after 10 amino acids peptide length.
Response: We have now provided evidence that DNPBS-His(Trt)-OH and DNPBS-Cys(Trt)-OH can be used during Fmoc SPPS. The orthogonality of the DNPBS SPPS

and Fmoc SPPS allows the two methodologies to be combined for the synthesis of long peptides with improved purity. Please see Figure 5d.

Data and methodology

The authors have attached all synthesis routes, the specific NMR data and further analytical data. In view of the work, all necessary data is shown.

Suggested improvements

The authors should validate the potential influence of the used resin on the peptide length be switching to a resin for more polar solvents with PEG linkers like Tentagel resins or totally PEG based resin like ChemMatrix. Testing different greener solvents is highly advised if the point of sustainability should still be included in the publication: Switching the solvents would also be an option to check whether the limitation of 10 amino acids is related to the solvent (would be possible with DMF as well).

Response: We thank the reviewer for the invaluable suggestions. We have now tried alternative solvents/resin and found that DNPBS SPPS can be used for synthesis of longer peptides based on Chemmatrix resin. These additional results are shown in Fig. 3e, 3f and 5d.

Clarity and context

The test is well readable and presented in an understandable way. The analytical data in the paper and the supplement underline the work of the authors and most of the conclusions. Some additional work in the field of green solvents, sustainable chemistry with focus on peptide synthesis should be included (see above and references).

References

Actual publications in the field of (sustainable) peptide synthesis regarding alternative organic solvents or water approaches based have not been considered (e.g. water based: Chem. Pharm. Bull. 2004, 52, 422-427; Tetrahed. Lett. 2004, 45, 9293; Angew. Chem. Int. Ed. 2020, 59, 12984; Organic: ChemSusChem 2021, 14, 3231; Jad et al. used 2-MeTHF γ-Valero-lactone N-formylmorpholine (NFM); Lawrenson et al. used propylene carbonate (PC)) and some should be added if the if the claim to sustainability in the paper should be maintained.

Response: These references have now been included in the revised manuscript. Please see Ref. 18-23.

Reviewer #3 (Remarks to the Author):

This work presents a new amino group protection/deprotection strategy for peptide synthesis that yields peptides in which alpha-carbon racemization and aspartimide formation is suppressed efficiently. The latter two reactions are typical side reactions found in peptide synthesis using the broadly applied Fmoc or Boc protection group strategies. The side products are usually hard to remove from the desired product due

to the high similarity (i.e. retention time in RP-HPLC), and a solution to this problem would thus be of great benefit. The authors produced all natural amino acids with the DNPBS protecting group and applied them for peptides synthesis. They nicely demonstrate that side products could be suppressed efficiently, which was most evident with amino acids such as His and Cys where racemization happens more frequently. The authors show that the method is suited for solid phase peptide synthesis, though not for longer peptides due to problems with protection group stability or too early removal of the protection group. These latter problems are not so well characterized as described in the major point 1 below. Another attractive application of the new protecting group is the generation of branched peptides, as demonstrated by the authors.

The work and results are appealing to me overall for the two main reasons: i) the work addresses a central problem (racemization of alpha carbons and aspartimide formation) and ii) the strategy is new and different than established amino-protecting chemistries. A major limitation is that it appears to work not as robust as Fmoc or Boc chemistry, as seen with side products and the problems with longer peptides. I recommend to characterize the exact problems in more detail (see major point 1 below). To benefit from the method already, one option may be to make longer peptides using Fmoc chemistry and to use DNPBS building blocks for amino acids that are prone to racemization (His, Cys). I recommend to test such a "hybrid" option, in order to complement the work with an appealing application for a broad audience (see major point 2 below).

Major:

1. Problems of new protecting group and side products:

The new strategy for amino group protection/deprotection appears to be not suited for longer peptides, as transparently stated/concluded by the authors. Some data points to the chemical basis of the problems (e.g. figure 3a), by providing examples of side products, but all this is not much detailed. I recommend that the authors perform a detailed side-by-side comparison of the new "DNPBS" method and Fmoc synthesis in terms of coupling efficiency on solid phase. E.g. they could do a similar comparison as in Figure 2b, for different amino acids (but testing the coupling on solid phase, as this is relevant).

Response: We understand the reviewer’s concern. The side products of DNPBS SPPS had been characterized based on the synthesis of short peptides **38-41** (Figure 3c). The major side products were ascribed to sequence deletion. Redundant Met and His incorporation had also been observed. In addition, the coupling yield decreased as the peptide chain length increased. These results suggest there are two major problems for the DNPBS SPPS: (1) The DNPBS protected Met and His are not stable enough during the coupling step. We have shown that minimizing the coupling time can effectively avoid the redundant incorporation. (2) The coupling yield of DNPBS SPPS seems not good as that of Fmoc SPPS. Based on their chemical structures and solubility, DNPBS protected amino acids are more hydrophobic than Fmoc protected ones. The hydrophobic interaction of DNPBS protected amino acids with polystyrene resin may

account for the decreased coupling efficiency. The problem becomes more serious when the resin shrinkage occurs as the peptide chain length increased, which preclude the synthesis of peptide longer than 10 residues.

According to the reviewer 2' suggestion, we have tried alternative solvents/resin and found that DNPBS SPPS can be used for synthesis of longer peptides based on ChemMatrix resin. We compared the synthetic efficiency of DNPBS SPPS and Fmoc SPPS by synthesizing peptide **43** (15 residues in length) on ChemMatrix resin and found that DNPBS SPPS was slightly less efficient than Fmoc SPPS (Figure 3f). Thus, the unsatisfied coupling yield is a weakness of DNPBS SPPS and remains to be improved in the future.

2. Problems with longer peptides:
The new approach is obviously not suited for longer peptides (as transparently indicated by the authors), which will limit much the application of the DNPBS protecting group. The authors indicate that it may be used selectively for some amino acids where razemization on the alpha carbon is particularly a problem (e.g. Fmoc-His, Fmoc Cys), wherein other couplings are done with Fmoc amino acids. I find this an interesting idea and recommend to assess if this works. E.g. the authors could apply the hybrid method to 2-3 longer peptides (e.g. well-known biologically active peptide) that suffered from racemization in Fmoc synthesis, and they could assess if the new "DNPBS" method offers an advantage if applied in combination with Fmoc chemistry.
Response: We thank the reviewer for the good suggestion. We have synthesized a model 20 mer oligopeptide containing all 20 natural amino acids on ChemMatrix resin by the combining of DNPBS and Fmoc SPPS. The histidine and cysteine residues in the sequence were incorporated by means of DNPBS SPPS, and all the other residues were incorporated by means of standard Fmoc SPPS. UPLC analysis of the crude product showed that the combined strategy afforded the target product in 42% yield (Fig. 5d). Of note, there were no impurities that co-elute with the main product. In contrast, synthesis of the same sequence based on Fmoc SPPS produced the aim product together with some co-eluting impurities (Fig. 5d), making it hard to obtain pure peptide by routine purification methods. Therefore, the combined strategy can afford cleaner final products than Fmoc SPPS. In addition, we had demonstrated that a combination of DNPBS SPPS and Fmoc SPPS could be employed for the synthesis of branched peptide.

Thus, although the coupling efficiency of DNPBS SPPS is not comparable to the that of standard Fmoc SPPS especially for long oligopeptides, the orthogonality of the DNPBS SPPS and Fmoc SPPS allows these two methodologies to be combined to synthesize peptides that would be difficult to prepare by either method alone.

Minor:
- Discussion: the authors repeat the advantages of the new method by indicating "4 merits", starting with the ability to monitor the deprotection due to absorbance, followed by suppression of alpha-carbon racemization etc. I recommend to list the merits in the order of importance (i.e. starting with the racemization suppression).

Response: We thank the reviewer for the good suggestion. In the revised manuscript, we have re-organized the order of the merits of DNPBS SPPS based on importance. Please see the Discussion section.

-Title: the authors used the term "cleaner" which I think is not ideal as the coupling efficiency is less good than in Fmoc synthesis (and the method thus actually less clean). I recommend to indicate the true advantages (e.g. racemization-free …)

Response: We understand the reviewer's concern. α-C racemization is an inherent drawback of conventional SPPS. α-C racemic products are hard to remove by routine purification methods and thus contaminate the final product.

DNPBS SPPS effectively avoids the generation of α-C racemic products. Although the coupling efficiency of DNPBS SPPS is not comparable to that of standard Fmoc SPPS, the side products of DNPBS SPPS arising from sequence deletion and redundant incorporation can be easily removed by routine purification methods. In this regard, we can claim that DNPBS SPPS affords cleaner final products than conventional SPPS. Thus, we would like to keep the current article title unchanged.

REVIEWERS' COMMENTS

Reviewer #1 (Remarks to the Author):

The authors have noted and responded to this reviewers concerns and requests for edits and additions. Overall, it is an improved manuscript compared to the original version and the authors are to be commended for making the necessary significant effort. That said, I remain convinced that the results are preliminary given that the overall synthetic yields remain modest inferior to established protocols and that the length of peptide production is short. Therefore, the revised manuscript remains better suited to a specialist journal. I shall leave it to the handling editor to make the final judgement.

Reviewer #2 (Remarks to the Author):

The authors introduced a new N-terminal protecting group (2,4-dinitro-6-phenyl-benzene sulfenyl group). This group is attached via an S-N bond. Removal is by M p-toluenethiol/pyridine and mild alkaline conditions. During their work, the authors introduced the new protecting group to the 20 canonical amino acids and carried out coupling experiments to determine the racemization of His, Ser and Cys. This was followed by some solid-phase peptide synthesis of model peptides with a comparison of the classical Fmoc-SPPS side reactions (racemisation, aspartimide formation). They also demonstrated orthogonality with the Fmoc group. This allows the DNPBS group to be used for branched synthesis or targeted side-chain modifications.
For their rework, they have added alternative solvents as well as different types of resins and tried to synthesize longer peptides. They pointed out that DNPBS SPPS can be used for the synthesis of longer peptides based on ChemMatrix resin. However, it is less efficient than Fmoc SPPS. A weakness of DNPBS SPPS that needs to be improved in the future is the reduced coupling yield. For the time being, the main purpose of the DNPBS protective group would be to reduce the racemization of His/Cys during Fmoc-SPPS. This would improve the yield of peptides that would be difficult to produce by either method alone.
Since the authors' own data show that this is not valid, the point "The use of DNPBS greatly suppresses the main side reactions observed in conventional SPPS, affording purer final products" should be removed.
In terms of sustainability (green chemistry), the authors still claim that their new strategy is valuable. The authors' additional experiments showed that the use of a greener coupling solvent such as 2-methyltetrahydrofuran, γ-valerolactone or N-formylmorpholine did not improve the synthetic yield. The best results were obtained using a DCM/MeCN system. Therefore, the sustainability claim should be removed.
Overall, the revised paper offers a new route for peptide synthesis that has some advantages over current synthesis strategies. This is achieved by combining the Fmoc and DNPBS strategies for some peptides. The paper therefore merits publication.

Reviewer #3 (Remarks to the Author):

The authors have addressed all major concerns. Specifically, they have synthesized a longer peptide and compared their new approach with standard Fmoc chemistry, and they combined the two methods.

They did not follow the suggestion of changing the title: I find the "Cleaner" still misleading as the new method is not cleaner but give less clean products (that may more easily be cleaned by HPLC). A good alternative title could be "Suppression of Alpha-Carbon Racemization in Peptide Synthesis Based on a Thiol-Labile Amino Protecting Group", or similar. I leave this to the editor though.

Given that all major points were addressed, I can recommend publication.

Reviewer #2 (Remarks to the Author):

Since the authors' own data show that this is not valid, the point "The use of DNPBS greatly suppresses the main side reactions observed in conventional SPPS, affording purer final products" should be removed.

Response: Statements such as "Affording purer final products", “Affording cleaner peptides” have been removed from the revised manuscript. Please see the Abstract, the last paragraph of Introduction, and the Discussion.

In terms of sustainability (green chemistry), the authors still claim that their new strategy is valuable. The authors' additional experiments showed that the use of a greener coupling solvent such as 2-methyltetrahydrofuran, γ-valerolactone or N-formylmorpholine did not improve the synthetic yield. The best results were obtained using a DCM/MeCN system. Therefore, the sustainability claim should be removed.

Response: All sustainability claims have been removed from the revised manuscript. However, we think the good solubility of DNPBS-protected amino acids in a broad range of organic solvents might make the use of DNPBS more attractive for liquid-phase peptide synthesis (LPPS) than for SPPS. Thus, we have retained this discussion in the Discussion section. Please see the last sentence of the Discussion.

Reviewer #3 (Remarks to the Author):

They did not follow the suggestion of changing the title: I find the “Cleaner” still misleading as the new method is not cleaner but give less clean products (that may more easily be cleaned by HPLC). A good alternative title could be “Suppression of Alpha-Carbon Racemization in Peptide Synthesis Based on a Thiol-Labile Amino Protecting Group”, or similar. I leave this to the editor though.

Response: Thank you for the good suggestion. We have changed the title to “Suppression of Alpha-Carbon Racemization in Peptide Synthesis Based on a Thiol-Labile Amino Protecting Group”.
